# Simplifying and Characterizing DAGs and Phylogenetic Networks via Least Common Ancestor Constraints

**DOI:** 10.1007/s11538-025-01419-z

**Published:** 2025-02-12

**Authors:** Anna Lindeberg, Marc Hellmuth

**Affiliations:** Department of Mathematics, Stockholm University, Stockholm, Sweden

**Keywords:** Phylogenetic networks, Reticulate evolution, Regular DAGs, Hasse diagram, Cluster, Transformation, NP-completeness, Algorithms

## Abstract

Rooted phylogenetic networks, or more generally, directed acyclic graphs (DAGs), are widely used to model species or gene relationships that traditional rooted trees cannot fully capture, especially in the presence of reticulate processes or horizontal gene transfers. Such networks or DAGs are typically inferred from observable data (e.g., genomic sequences of extant species), providing only an estimate of the true evolutionary history. However, these inferred DAGs are often complex and difficult to interpret. In particular, many contain vertices that do not serve as least common ancestors (LCAs) for any subset of the underlying genes or species, thus may lack direct support from the observable data. In contrast, LCA vertices are witnessed by historical traces justifying their existence and thus represent ancestral states substantiated by the data. To reduce unnecessary complexity and eliminate unsupported vertices, we aim to simplify a DAG to retain only LCA vertices while preserving essential evolutionary information. In this paper, we characterize $\textrm{LCA}$-relevant and $\textrm{lca}$-relevant DAGs, defined as those in which every vertex serves as an LCA (or unique LCA) for some subset of taxa. We introduce methods to identify LCAs in DAGs and efficiently transform any DAG into an $\textrm{LCA}$-relevant or $\textrm{lca}$-relevant one while preserving key structural properties of the original DAG or network. This transformation is achieved using a simple operator “$\ominus$” that mimics vertex suppression.

## Introduction

Rooted networks and, more generally, directed acyclic graphs (DAGs), are essential in mathematical phylogenetics for modeling complex evolutionary relationships that traditional rooted trees cannot fully represent (Huber et al. 2022; Huson et al. 2011; Huson and Scornavacca 2011). In a DAG *G*, the leaf set *L*(*G*) represents extant taxa, such as genes or species, while internal vertices $v \in V(G)\setminus L(G)$ correspond to ancestral states and are associated with sets ${{\,\mathrm{\texttt{C}}\,}}_G(v)$ of descendant leaves known as “hardwired clusters” (Huson and Scornavacca 2011; van Iersel et al. 2010; Huson and Rupp 2008), or *clusters* (Hellmuth et al. 2023) for short. Typically, only the leaf set *L*(*G*) is available and a primary task is to reconstruct the evolutionary history—i.e., phylogenetic networks or DAGs—using information provided solely by the taxa in *L*(*G*). This information, often derived from genomic sequence data and sequence similarities, can reveal clusters within the unknown DAG *G* that are used to reconstruct *G* (van Iersel et al. 2010; Huson and Rupp 2008; Huson and Scornavacca 2011; Gambette et al. 2017).

However, DAGs and networks inferred from genomic data can be highly complex and tangled, often containing redundant information (Dagan and Martin 2009; Francis et al. 2021). In particular, unlike phylogenetic trees, the number of vertices in a DAG *G* is in general not asymptotically bounded by a function depending solely on the number of leaves. As a result, various methods have been developed to simplify DAGs while preserving their most significant features (Heiss et al. 2025; Francis et al. 2021; Huber et al. 2016). Our research builds on this line of work and focuses on eliminating vertices from a DAG *G* that are “less relevant” in the sense that they are not least common ancestors of certain subsets of *L*(*G*).

A least common ancestor (LCA) of a subset $A \subseteq L(G)$ is a vertex *v* that is an ancestor of all $x \in A$ and that has no descendant which also satisfies this property. LCAs are essential for understanding and interpreting evolutionary relationships in phylogenetics (Schaller et al. 2021; Hellmuth et al. 2015; Lafond et al. 2016; Nøjgaard et al. 2018). In evolutionary biology, there is a general consensus that inferred networks and DAGs should be phylogenetic, that is, they should not contain vertices with in- and out-degree one. The reason is simple: such vertices cannot be observed from any biological data since there is no historical trace left justifying their existence (Hellmuth 2017). By similar reasoning, LCA vertices should represent ancestral relationships evidenced by a clear phylogenetic signal in the observable data, i.e., the leaves in *G*. Vertices that are not LCAs of any subset of taxa of the underlying data may lack direct relevance to the observed ancestral relationships.

**Fig. 1:**
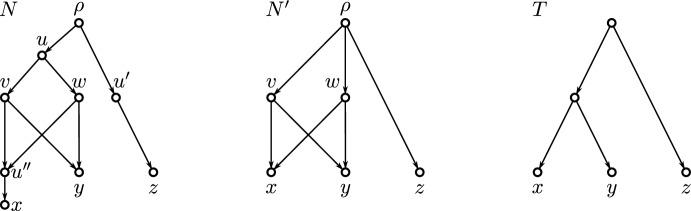
Shown are three networks *N*, $N'$ and *T*. All have the same clustering system $\mathfrak {C} = \{\{x\},\{y\},\{z\},\{x,y\},\{x,y,z\}\}$ and leaf set $X = \{x,y,z\}$. Here, only $N'$ and *T* are phylogenetic. The network *N* is not phylogenetic, since *N* contains the vertex $u'$ with in- and out-degree one. Moreover, vertices *u*, $u'$ and $u''$ in *N* are not LCAs of any subset of leaves. “Removing” *u*, $u'$ and $u''$ from *N* via the “$\ominus$”-operator—as explained in detail in Sect. 5—yields the simplified network $N' = N\ominus \{u,u',u''\}$ in which all vertices are LCAs of some subset of *X*. Hence, $N'$ is $\operatorname {LCA}$-Rel. In particular, $N'$ is precisely the simplification $\varphi _{\operatorname {LCA}}(N)$ as explained in Sect. 6. However, $N'$ is not $\operatorname {lca}$-Rel as the vertices *v* and *w* are not unique LCAs in $N'$ for any subset of *X*. If desired, $N'$ can now be further simplified by “removing” one of *v* or *w* and resulting shortcuts which yields the phylogenetic and $\operatorname {lca}$-Rel tree *T*. The tree *T* is the unique phylogenetic tree whose clustering system $\mathfrak {C}$ is identical to those in *N* and $N'$

In the network *N* shown in Fig. 1, the three vertices *u*, $u'$, and $u''$ do not serve as the LCA for any subset of $L(N) = \{x, y, z\}$. Removing or suppressing these vertices using the “$\ominus$-operator” defined in Sect. 5 yields the simplified network $N'$. While the term “simplification” may be subjective and influenced by specific interests, the process described here satisfies fundamental axioms for network simplification as proposed by Heiss et al. (2025) (see Sect. 6 for more details). All vertices in $N'$ serve as an LCA for some subset of leaves and can thus be considered to be supported by historical traces observable in the data $L(N) = X$, a property not satisfied by the vertices *u*, $u'$, and $u''$ in *N*. For instance, the vertex $u'$ in *N* has in- and out-degree one, with no justification for its existence provided by the set of leaves. Similarly, since *u* and $u''$ are not LCAs for any subset of *X*, they can be considered less supported by the data. We are aware that *u* and $u''$ are “binary” vertices, i.e., they have in-degree one and out-degree two, and in-degree two and out-degree one, respectively. However, binary (fully resolved) networks often result from artificially resolving non-binary vertices and, thus, may impose assumptions about evolutionary history that extend beyond what is provided by the data (Hellmuth et al. 2015; van Iersel et al. 2010). This provides justification for removing some binary vertices as well. Again, the primary reason to suppress vertices *u* and $u''$ here is that they do not serve as the LCA of any subset of leaves. Note that $u''$ can also be considered redundant because the reticulation event leading to taxa *x* it represents is still captured in $N'$ after suppressing $u''$. If one is additionally interested in networks that include only vertices representing unique LCAs of subsets of leaves, one can further simplify $N'$ into the phylogenetic tree *T* by removing one of *v* or *w*. Notably, *T* is the unique phylogenetic tree whose clustering system is identical to those in *N* and $N'$. In general, the networks $N'$ and *T* can always be considered as simplifications of *N* showing a “trend of evolution”.

As argued above, non-LCA vertices may lack clear interpretation and could be considered less significant or redundant in an evolutionary context. Therefore, simplifying a DAG by “removal” of non-LCA vertices resulting in a DAG in which each vertex is a (unique) LCA of at least some leaves is a natural next step. We demonstrate that this transformation can be performed efficiently while preserving the structural integrity of the original DAG. The central questions considered here are as follows:

1. Is a given vertex a (unique) LCA of a specific, known subset $A \subseteq L(G)$?
2. Is a given vertex a (unique) LCA of some unknown subset $A \subseteq L(G)$, possibly with a prescribed size |*A*|?
3. Can one characterize and recognize DAGs *G* in which every vertex is a (unique) LCA of some subset of *L*(*G*)?
4. Is it possible to efficiently remove all vertices from a DAG *G* that do not satisfy (1) or (2) and thus, to simplify *G* to a DAG in which each vertex is a (unique) LCA of some subset of *L*(*G*) while preserving as many structural features of *G* as possible?

We will address these problems from different perspectives. Numerous results have been established for Question 1 and 2 for the case $|A|=2$ (Kowaluk and Lingas 2007; Nykänen and Ukkonen 1994; Bender et al. 2005; Mathialagan et al. 2022; Czumaj et al. 2007; Grandoni et al. 2021; Bender et al. 2001; Kowaluk and Lingas 2005; Harel and Tarjan 1984), or when assuming that $A = {{\,\mathrm{\texttt{C}}\,}}_G(v)$ for a given vertex *v* (Nakhleh and Wang 2005).

This paper is organized as follows. We start with introducing the basic definitions needed in Sect. 2. In Sect. 3, we define the notions of $\operatorname {lca}$-Rel and $\operatorname {LCA}$-Rel DAGs, as well as *k*-$\operatorname {lca}$ and *k*-$\operatorname {LCA}$ vertices. In short, a vertex *v* is a *k*-$\operatorname {LCA}$ (resp., *k*-$\operatorname {lca}$) in *G* if there exists a subset $A\subseteq L(G)$ of size $|A|=k$ such that *v* is a LCA (resp., unique LCA) of the vertices in *A*. As we will see, a vertex *v* is a *k*-$\operatorname {LCA}$ vertex (resp., *k*-$\operatorname {lca}$ vertex) for some *k* precisely if *v* is a LCA (resp., unique LCA) of the vertices in ${{\,\mathrm{\texttt{C}}\,}}_G(v)$ (cf. Corollary 3.6). A DAG is $\operatorname {LCA}$-Rel (resp., $\operatorname {lca}$-Rel) if each of its vertices is a LCA (resp., unique LCA) for some subset *A*. We then show that the set of least common ancestors of a set $A \subseteq L(G)$ can be determined in linear time when $|A| \in O(1)$ is constant. Additionally, we demonstrate that recognizing $\operatorname {lca}$-Rel and $\operatorname {LCA}$-Rel DAGs can be done in polynomial time.

In Sect. 4, we continue by characterizing $\operatorname {lca}$-Rel and $\operatorname {LCA}$-Rel DAGs. As shown in Theorem 4.4, $\operatorname {LCA}$-Rel DAGs are precisely those DAGs that do not contain adjacent vertices *u* and *v* with the same cluster, i.e., ${{\,\mathrm{\texttt{C}}\,}}_G(v)={{\,\mathrm{\texttt{C}}\,}}_G(u)$. We then provide several characterizations of $\operatorname {lca}$-Rel DAGs in Theorem 4.5. Among other results, $\operatorname {lca}$-Rel DAGs are exactly those $\operatorname {LCA}$-Rel DAGs with the path-cluster-comparability (PCC) property, meaning there is a directed path between two vertices *v* and *u* if and only if their clusters are comparable with respect to inclusion. Moreover, we show a close connection between $\operatorname {lca}$-Rel DAGs and so-called regular DAGs where the latter, loosely speaking, are DAGs that are completely determined by their set of clusters. In particular, $\operatorname {lca}$-Rel DAGs with all shortcuts removed are regular (cf. Corollary 4.9). Novel characterizations of regular DAGs are presented in Theorem 4.10. Similar to phylogenetic trees, the number of vertices in $\operatorname {lca}$-Rel DAGs *G* is asymptotically bounded above by a function on the number of leaves, see Lemma 4.12.

Not all DAGs are $\operatorname {lca}$-Rel or $\operatorname {LCA}$-Rel. Hence, the question arises whether one can transform a given DAG *G* into an $\operatorname {lca}$-Rel or $\operatorname {LCA}$-Rel DAG *H* while preserving as much of the structure of *G* as possible. In Sect. 5, we provide an axiomatic framework for the phrase “*preserving as much structure of G as possible*” resulting in five structural properties (S1)–(S5). These properties are informally stated here and will be defined more precisely in Sect. 5.

*(S1)*: *H* remains a DAG with leaf set *L*(*G*).
*(S2)*: $V(H) \subseteq V(G)$, meaning no new vertices are introduced.
*(S3)*: *H* preserves the ancestor relationships defined by *G* among the vertices in *H*.
*(S4 &S5)*: The set of (unique) least common ancestors for (un)specified subsets $A\subseteq L(G)$ in *G* and *H* coincide.

This in turn implies an additional condition *(S0)*: no new clusters in *H* are introduced.

To transform a given DAG into an $\operatorname {lca}$-Rel or $\operatorname {LCA}$-Rel one, we introduce a simple operator $\ominus$ that acts on the vertices and edges of *G* (Shanavas et al. 2024). Specifically, we denote with $G\ominus v$ the DAG obtained from *G* by removing vertex *v* and its incident edges and connecting each parent of *v* with each child of *v*. Using this method, vertices *v* can be removed stepwise from *G*, resulting in an $\operatorname {lca}$-Rel or $\operatorname {LCA}$-Rel DAG *H* that satisfies the properties (S0)–(S5). In particular, we provide conditions under which the set *W* of vertices such that $G \ominus W$ is $\operatorname {lca}$-Rel or $\operatorname {LCA}$-Rel is uniquely determined and of minimum size. Furthermore, polynomial-time algorithms are given to transform any DAG into an $\operatorname {lca}$-Rel or $\operatorname {LCA}$-Rel one. The established algorithms are implemented in the Python package SimpliDAG (Lindeberg and Hellmuth 2024).

Following (Heiss et al. 2025), we discuss in Sect. 6 a general framework for any transformation $\varphi (G)$ that “simplifies” a DAG *G*, formalized through three axioms (P1)–(P3). Given a suitable notion of restriction of DAGs, we show that the $\ominus$-operator can be used to derive simplifications $\varphi (G)$ that satisfy axioms (P1)–(P3). We exemplify different types of simplification steps on a biological network with reticulation events that is based on a study of the *Viola* genus from Marcussen et al. (2014).

While we have provided polynomial-time algorithms to verify whether a given DAG *G* is $\operatorname {lca}$-Rel or $\operatorname {LCA}$-Rel and to transform *G* into an $\operatorname {lca}$-Rel or $\operatorname {LCA}$-Rel DAG if it is not, an open question remained so-far: can it be decided in polynomial-time if a vertex *v* is a *k*-$\operatorname {lca}$ or *k*-$\operatorname {LCA}$ vertex for a given *k*? Although the answer is affirmative for $k=1$ or $k=2$ (cf. e.g. (Nowak (2009), Thm. 4.19) or Observation 7.6), we show in Sect. 7 that this problem is NP-complete in general. However, it becomes polynomial-time solvable for DAGs with the (N3O) property, i.e., DAGs that do not contain three pairwise overlapping clusters. Such DAGs are of particular interest, as they include important subclasses of phylogenetic networks, such as rooted phylogenetic trees and galled-trees.

We close this paper with Sect. 8, where we summarize the main results and provide open problems for future work.

## Basics

***Sets and Set Systems.*** All sets considered here are assumed to be finite. Here, $2^X$ denotes the powerset of a set *X*. For $\mathscr {I}\subseteq \{1,\dots , |X|\}$, we write $X(\mathscr {I}) \subseteq 2^X$ for the set of all subsets *A* of *X* with $|A|\in \mathscr {I}$. Two sets *M* and $M'$ *overlap* if $M \cap M' \notin \{\emptyset , M, M'\}$.

A *set system *$\mathfrak {C}$ (*on* *X*) is a subset $\mathfrak {C} \subseteq 2^X$. A set system $\mathfrak {C}$ on *X* is *grounded* if $\{x\}\in \mathfrak {C}$ for all $x\in X$ and $\emptyset \notin \mathfrak {C}$, while $\mathfrak {C}$ is a *clustering system* if it is grounded and satisfies $X\in \mathfrak {C}$. Furthermore, a set system *satisfies (N3O)* if it does not contain three distinct pairwise overlapping clusters.

***Directed Graphs, DAGs and Networks.*** A *directed graph* $G=(V,E)$ is an ordered pair consisting of a nonempty set $V(G):=V$ of *vertices* and a set $E(G):=E \subseteq \left( V\times V\right) {\setminus }\{(v,v) \mid v\in V\}$ of *edges*. For directed graphs $G=(V_G,E_G)$ and $H=(V_H, E_H)$, an *isomorphism between G and H* is a bijective map $\varphi :V_G\rightarrow V_H$ such that $(u,v)\in E_G$ if and only if $(\varphi (u),\varphi (v))\in E_H$. If such a map exist, then *G* and *H* are *isomorphic*, in symbols $G\simeq H$.

A $v_1v_n$-*path* $P = (V,E)$ has an ordered vertex set $V = \{v_1,v_2,\ldots ,v_n\}$ and the edges in *E* are precisely of one of the form $(v_i,v_{i+1})$ or $(v_{i+1},v_i)$, $i=1,2,\ldots , n-1$. The *length* of *P* is the number $|E|=n-1$ of its edges. A directed graph *G* is *connected* if there exists an *xy*-path between any pair of vertices *x* and *y*. If all edges in *P* are precisely of the form $(v_i,v_{i+1})$ for each $i=1,2,\ldots , n-1$, then *P* is called *directed*.

For a directed graph $G=(V,E)$, we define ${{\,\textrm{indeg}\,}}_G(v):=\left| \left\{ u\in V :(u,v)\in E\right\} \right|$ and ${{\,\textrm{outdeg}\,}}_G(v):=\left| \left\{ u \in V:(v,u)\in E\right\} \right|$ for each $v\in V$ as the *in-degree* respectively *out-degree* of *v* in *G*. A directed graph *G* is *phylogenetic*, if it does not contain a vertex *v* with ${{\,\textrm{outdeg}\,}}_G(v)=1$ and ${{\,\textrm{indeg}\,}}_G(v)\le 1$.

A directed graph *G* is *acyclic* if there exist no *directed cycle*, that is, no sequence of $k\ge 2$ distinct vertices $v_1,v_2,\ldots , v_k\in V$ such that $(v_1,v_2),(v_2,v_3),\ldots ,(v_{k-1},v_{k}),(v_k,v_1)\in E$. A directed acyclic graph is called *DAG*. An edge $e=(u,w)$ in a DAG *G* is a *shortcut* if there is a directed *uw*-path that does not contain the edge *e* (Linz and Semple 2020; Döcker et al. 2019). A DAG without shortcuts is *shortcut-free*.

Let *G* be a DAG with an associated partial order $\preceq _G$ on its vertex set *V*(*G*) defined by $v\preceq _G w$ if and only if there is a directed path (possibly of length zero) from *w* to *v*. In this case, we say that *w* is an ancestor of *v* and *v* is a descendant of *w*. If $v\preceq _G w$ and $v\ne w$, we write $v\prec _G w$. Two vertices $u,v\in V(G)$ are $\preceq _{G}$-*incomparable* if neither $u\preceq _G v$ nor $v\preceq _G u$ is true. We denote by $L(G)\subseteq V(G)$ the $\preceq _G$-minimal vertices of *G* and we call $x\in L(G)$ a leaf of *G*. Note that ${{\,\textrm{outdeg}\,}}_G(x)=0$ for all $x\in L(G)$. It easy to verify that $\preceq _G$-minimal vertices must exist in any DAG *G*: take a longest directed *uv*-path *P* in *G*, i.e., *P* has a maximum number of edges among all paths in *G*. In this case *v* must be a leaf as, otherwise, there is an edge (*v*, *w*) such that either $w\notin P$ in which case *P* was not a longest directed path or $w\in P$ in which case *G* contains a cycle; both cases leading to a contradiction. Thus, $L(G)\ne \emptyset$ for all DAGs *G*. A vertex of *G* that is not contained in *L*(*G*) is called an *inner vertex*. Moreover, if (*u*, *v*) is an edge of *G*, then *u* is a *parent* of *v*, while *v* is a *child* of *u*. We let ${{\,\textrm{child}\,}}_G(v)$ denote the set of all children of a vertex *v*.

If $L(G)=X$, then *G* is a *DAG on X*. A vertex $v\in V(G)$ of a DAG *G* that is $\preceq _G$-maximal is called a *root* and the set of roots of *G* is denoted by *R*(*G*). Note that ${{\,\textrm{indeg}\,}}_G(r)=0$ for all $r\in R(G)$. By similar arguments as for leaves, $R(G)\ne \emptyset$ for all DAGs *G*. For every $v\in V(G)$ in a DAG *G*, the set of its descendant leaves

$$\begin{aligned} {{\,\mathrm{\texttt{C}}\,}}_G(v):=\{ x\in L(G)\mid x \preceq _G v\} \end{aligned}$$

is a *cluster* of *G*. We write $\mathfrak {C}_G:=\{{{\,\mathrm{\texttt{C}}\,}}_G(v)\mid v\in V(G)\}$ for the set of all clusters in *G*. By construction, ${{\,\mathrm{\texttt{C}}\,}}_G(x)=\{x\}$ for all $x\in L(G)$. Moreover, for all $v\in V(G)$, there are vertices *x* with $x \preceq _G v$ that are $\preceq _G$-minimal and, thus contained in *L*(*G*). Hence, ${{\,\mathrm{\texttt{C}}\,}}_G(v)\ne \emptyset$ for all $v\in V(G)$, in particular $\emptyset \notin \mathfrak {C}_G$ holds. Therefore, $\mathfrak {C}_G$ is a grounded set system on *L*(*G*) for every DAG *G*. For later reference we provide

### Lemma 2.1

(Shanavas et al. (2024), Lem. 1) For all DAGs *G* and all $u,v\in V(G)$ it holds that $u\preceq _G v$ implies ${{\,\mathrm{\texttt{C}}\,}}_G(u)\subseteq {{\,\mathrm{\texttt{C}}\,}}_G(v)$.

**Fig. 2:**
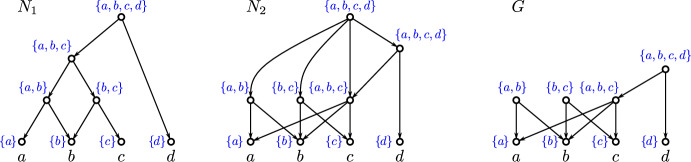
Shown are two phylogenetic networks $N_1$ and $N_2$ and a phylogenetic DAG *G* such that $\mathfrak {C}_{N_1} = \mathfrak {C}_{N_2} = \mathfrak {C}_G$. The clusters ${{\,\mathrm{\texttt{C}}\,}}(v)$ are drawn next to each individual vertex *v* and highlighted by blue text. Out of the shown DAGs, only $N_1$ satisfies (PCC), is regular and has the strong-(CL) property (i.e., $v = \operatorname {lca}_{N_1}({{\,\mathrm{\texttt{C}}\,}}_{N_1}(v))$ for all *v* in $N_1$; cf. Def. 4.1). Moreover, only $N_1$ is $\operatorname {lca}$-Rel and $\operatorname {LCA}$-Rel (cf. Def. 3.7). Here, *G* is $\operatorname {LCA}$-Rel but $N_2$ is not (Color figure online)

The converse of Lemma 2.1 is, in general, not satisfied. By way of example, consider the DAG *G* in Fig. 2, where the three roots $r_1$, $r_2$ and $r_3$, read from left to right, are pairwisely $\preceq _G$-incomparable but satisfy ${{\,\mathrm{\texttt{C}}\,}}_G(r_1), {{\,\mathrm{\texttt{C}}\,}}_G(r_2)\subsetneq {{\,\mathrm{\texttt{C}}\,}}_G(r_3)$.

The following simple result shows that the cluster associated with a vertex can be expressed as the union of the clusters associated to its children.

### Lemma 2.2

Let $G=(V,E)$ be a DAG on *X* and $A\subseteq X$ nonempty. For all inner vertices $v\in V$, it holds that

$$\begin{aligned} {{\,\mathrm{\texttt{C}}\,}}_G(v) = \bigcup _{u\in {{\,\textrm{child}\,}}_G(v)} {{\,\mathrm{\texttt{C}}\,}}_G(u) \quad \text {and}\quad A\setminus {{\,\mathrm{\texttt{C}}\,}}_G(v)=\bigcap _{u\in {{\,\textrm{child}\,}}_G(v)}\left( A\setminus {{\,\mathrm{\texttt{C}}\,}}_G(u)\right) . \end{aligned}$$

### Proof

Let *v* be an inner vertex of *G*. Lemma 2.1 implies that $\cup _{u\in {{\,\textrm{child}\,}}_G(v)} {{\,\mathrm{\texttt{C}}\,}}_G(u)\subseteq {{\,\mathrm{\texttt{C}}\,}}_G(v)$. Now, let $x\in {{\,\mathrm{\texttt{C}}\,}}_G(v)$. Since $x\prec v$, there is a directed path from *v* to *x* and consequently a child *u* of *v* with $x\preceq u$ and thus, $x\in {{\,\mathrm{\texttt{C}}\,}}_G(u)$. Hence, ${{\,\mathrm{\texttt{C}}\,}}_G(v) = \cup _{u\in {{\,\textrm{child}\,}}_G(v)} {{\,\mathrm{\texttt{C}}\,}}_G(u)$. Therefore, $A{\setminus }{{\,\mathrm{\texttt{C}}\,}}_G(v)=A{\setminus } (\cup _{u\in {{\,\textrm{child}\,}}_G(v)}{{\,\mathrm{\texttt{C}}\,}}_G(u) )=\cap _{u\in {{\,\textrm{child}\,}}_G(v)} (A{\setminus }{{\,\mathrm{\texttt{C}}\,}}_G(u) )$. $\square$

A *(rooted) network* *N* is a DAG for which $|R(N)|=1$, i.e., *N* has a unique root $\rho \in V(N)$. In a network *N*, we have $v\preceq _N \rho$ for all $v\in V(N)$ and, thus, in particular, ${{\,\mathrm{\texttt{C}}\,}}_N(\rho )=X$, i.e., $X\in \mathfrak {C}_N$. Hence, $\mathfrak {C}_N$ is a clustering system (cf. (Hellmuth et al. (2023), Lem. 14)). The converse, however, is in general not satisfied, see Fig. 2 for an example where *G* is not a network but $\mathfrak {C}_G$ is a clustering system. A network *N* is a *tree*, if there is no vertex *v* with ${{\,\textrm{indeg}\,}}(v)>1$.

Lemma 2.1 shows that if two vertices are $\preceq _G$-comparable, then their respective clusters are comparable with respect to inclusion. The following property ensures the converse, namely, $\preceq _G$-comparability of vertices *u* and *v* based on subset-relations between the underlying clusters ${{\,\mathrm{\texttt{C}}\,}}_G(u)$ and ${{\,\mathrm{\texttt{C}}\,}}_G(v)$.

### Definition 2.3

A DAG *G* has the path-cluster-comparability (PCC) property if it satisfies, for all $u, v \in V(G)$: *u* and *v* are $\preceq _G$-comparable if and only if ${{\,\mathrm{\texttt{C}}\,}}_G(u) \subseteq {{\,\mathrm{\texttt{C}}\,}}_G(v)$ or ${{\,\mathrm{\texttt{C}}\,}}_G(v) \subseteq {{\,\mathrm{\texttt{C}}\,}}_G(u)$.

By Hellmuth et al. (2023, Lem. 24), for every clustering system $\mathfrak {C}$ there is a network *N* with $\mathfrak {C}_N = \mathfrak {C}$ that satisfies (PCC). This result builds on the concepts of Hasse diagrams and regular networks. Before delving into the properties of these specific types of DAGs, we first demonstrate that the $\preceq _G$-ancestor relationship and, consequently (PCC), is preserved under the removal of shortcuts.

### Definition 2.4

We denote with $G^-$ the DAG obtained from the DAG *G* by removal of all shortcuts.

### Lemma 2.5

Let $G = (V,E)$ be a DAG on *X* with $\ell >0$ shortcuts and let *e* be a shortcut in *G*. Then, $G':=(V,E\setminus \{e\})$ is a DAG on *X* with $\ell -1$ shortcuts. In particular, $G^-$ is uniquely determined for all DAGs *G*. Moreover, for all $u,v\in V$, it holds that $u\prec _G v$ if and only if $u\prec _{G'} v$ and, for all $v\in V$, it holds that ${{\,\mathrm{\texttt{C}}\,}}_G(v)= {{\,\mathrm{\texttt{C}}\,}}_{G'}(v)$. Furthermore, *G* satisfies (PCC) if and only if $G'$ satisfies (PCC).

### Proof

Let $G = (V,E)$ be a DAG on *X* with $\ell >0$ shortcuts and let *e* be a shortcut in *G*. Put $G' :=(V,E\setminus \{e\})$. Reusing exactly the same argument as used in the proof of (Hellmuth et al. (2023), Lem. 1), where an analogous result was provided for networks, shows that $G'$ is a DAG on *X* such that, for all $u,v\in V$, it holds that $v\prec _G u$ if and only if $v\prec _{G'} u$ and, for all $v\in V$, it holds that ${{\,\mathrm{\texttt{C}}\,}}_G(v)= {{\,\mathrm{\texttt{C}}\,}}_{G'}(v)$. Since $e = (a,b)$ is a shortcut, there is a directed *ab*-path $P_{ab}$ that does not contain *e*. Consider now an arbitrary edge $f=(u,v)\ne e$ of *G*. If *f* is a shortcut of *G*, there is a directed *uv*-path $P_{uv}$ in *G* that does not contain *f*. There are two cases: *e* is not an edge in $P_{uv}$, or it is. In the first case put $P:=P_{uv}$. In the latter case, replace the edge *e* in $P_{uv}$ by the path $P_{ab}$ and denote the resulting subgraph by *P*. Since *G* is a DAG, the edge *f* is not contained in $P_{ab}$ and thus *P* is a directed path that does not contain *f*. Clearly, *P* remains a directed *uv*-path in $G'$ that does not contain *f*, so *f* is a shortcut of $G'$. If, instead, *f* is not a shortcut of *G*, then any *uv*-path in *G* must coincide with the edge *f*. Clearly, any *uv*-path must coincide with the edge *f* in $G'$. In summary, an edge distinct from *e* is a shortcut of *G* if and only if it is a shortcut of $G'$. Consequently, $G'$ has $\ell -1$ shortcuts. The latter arguments directly imply that $G^-$ is uniquely determined.

Finally, suppose that *G* satisfies (PCC). Hence, ${{\,\mathrm{\texttt{C}}\,}}_G(u)\subseteq {{\,\mathrm{\texttt{C}}\,}}_G(v)$ precisely if *u* and *v* are $\prec _G$-comparable. Since $v\prec _G u$ if and only if $v\prec _{G'} u$ for all $u,v\in V$ and ${{\,\mathrm{\texttt{C}}\,}}_G(w)= {{\,\mathrm{\texttt{C}}\,}}_{G'}(w)$ for all $w\in V$, it immediately follows that $G'$ satisfies (PCC). By similar arguments, if $G'$ satisfies (PCC), then *G* satisfies (PCC). $\square$

***Hasse Diagrams and Regular DAGs.***

The *Hasse diagram* $\mathscr {H}(\mathfrak {C})$ of a set system $\mathfrak {C}\subseteq 2^X$ is the DAG with vertex set $\mathfrak {C}$ and directed edges from $A\in \mathfrak {C}$ to $B\in \mathfrak {C}$ if (i) $B\subsetneq A$ and (ii) there is no $C\in \mathfrak {C}$ with $B\subsetneq C\subsetneq A$. We note that $\mathscr {H}(\mathfrak {C})$ is also known as the *cover digraph* of $\mathfrak {C}$ Baroni et al. (2005). The Hasse diagram $\mathscr {H}(\mathfrak {C})$ is not necessarily phylogenetic. By way of example, for $\mathfrak {C} = \{\{x,y\},\{x\}\}$, $\mathscr {H}(\mathfrak {C})$ is a non-phylogenetic network since its unique root $\{x,y\}$ has out-degree 1 and in-degree 0. Nevertheless, if $\mathfrak {C}$ is a grounded set system, then the underlying Hasse diagram is phylogenetic, as we will show in Lemma 4.7.

In general, we are interested in DAGs *G* with certain properties and that satisfy $\mathfrak {C}_G=\mathfrak {C}$ for a given grounded set system $\mathfrak {C}$. Structural properties of $\mathscr {H}(\mathfrak {C})$ are, in this context, often helpful. However, $\mathfrak {C}_{\mathscr {H}(\mathfrak {C})}\ne \mathfrak {C}$ holds as the leaves of $\mathscr {H}(\mathfrak {C})$ are labeled with the inclusion-minimal elements in $\mathfrak {C}$, i.e., as sets. To circumvent this, we write

$$\begin{aligned} G\doteq \mathscr {H}(\mathfrak {C}) \end{aligned}$$

for the directed graph that is obtained from $\mathscr {H}(\mathfrak {C})$ by relabeling all vertices $\{x\}$ in $\mathscr {H}(\mathfrak {C})$ by *x*. Thus, for $G\doteq \mathscr {H}(\mathfrak {C})$ it holds that $\mathfrak {C}_G=\mathfrak {C}$ provided that $\mathfrak {C}$ is a grounded set system on *X*.

### Definition 2.6

(Baroni et al. 2005) A DAG $G=(V,E)$ is *regular* if the map $\varphi :V\rightarrow V(\mathscr {H}(\mathfrak {C}_G))$ defined by $v\mapsto {{\,\mathrm{\texttt{C}}\,}}_G(v)$ is an isomorphism between *G* and $\mathscr {H}(\mathfrak {C}_G)$.

We emphasize that not every DAG $G\doteq \mathscr {H}(\mathfrak {C})$ is regular. By way of example, consider the set system $\mathfrak {C} = \{\{x\},\{x,y\}\}$ where $G\doteq \mathscr {H}(\mathfrak {C})$ consists of a single edge and where each $v\in V(G)$ satisfies ${{\,\mathrm{\texttt{C}}\,}}_{G}(v) = \{x\}$, i.e., $\varphi :G\rightarrow V(\mathscr {H}(\mathfrak {C}_G))$ via $v\mapsto {{\,\mathrm{\texttt{C}}\,}}_{G}(v)$ will map both of the vertices of *G* to $\{x\}$ and thus, does not yield an isomorphism between *G* and $\mathscr {H}(\mathfrak {C})$. However, as we will see in Lemma 4.7, $\mathscr {H}(\mathfrak {C})$ is regular whenever $\mathfrak {C}$ is grounded.

## Least Common Ancestors and lca- & LCA-Relevant DAGs

For a given a DAG *G* and a subset $A\subseteq L(G)$, a vertex $v\in V(G)$ is a *common ancestor of A* if *v* is an ancestor of every vertex in *A*. Moreover, *v* is a *least common ancestor* (LCA) of *A* if *v* is a $\preceq _G$-minimal vertex that is an ancestor of all vertices in *A*. The set $\operatorname {LCA}_G(A)$ comprises all LCAs of *A* in *G*. In general, not every set $A\subseteq L(G)$ has a least common ancestor in a DAG: consider the DAG with three leaves $\{x,y,z\}$ and two $\prec _G$-maximal vertices *p*, *q* such that ${{\,\mathrm{\texttt{C}}\,}}_G(p)=\{x,y\}$ respectively ${{\,\mathrm{\texttt{C}}\,}}_G(q)=\{x,z\}$, in which case *x* and *y* have no common ancestor at all and, therefore, $\operatorname {LCA}_G(\{y,z\})=\emptyset$. In a network *N*, the unique root is a common ancestor for all $A\subseteq L(N)$ and, therefore, $\operatorname {LCA}_N(A)\ne \emptyset$. We now provide a simple characterization of vertices that belong to $\operatorname {LCA}(A)$, generalizing (Hellmuth et al. (2023), Lem. 35, Obs. 11) from networks to all DAGs.

### Lemma 3.1

For all DAGs $G=(V,E)$ on *X*, all nonempty subsets $A\subseteq X$ and vertices $v\in V$ the following statements are equivalent.

1. $$v\in \operatorname {LCA}_G(A)$$.
2. $A\subseteq {{\,\mathrm{\texttt{C}}\,}}_G(v)$ and $A\not \subseteq {{\,\mathrm{\texttt{C}}\,}}_G(u)$ for all $u\in {{\,\textrm{child}\,}}_G(v)$.
3. $A\subseteq {{\,\mathrm{\texttt{C}}\,}}_G(v)$ and $A\not \subseteq {{\,\mathrm{\texttt{C}}\,}}_G(u)$ for all $u\in V$ with $u\prec _G v$.

In particular, if $v\in \operatorname {LCA}_G(A)$ for some $\emptyset \ne A\subseteq X$, then ${{\,\mathrm{\texttt{C}}\,}}_G(u)\ne {{\,\mathrm{\texttt{C}}\,}}_G(v)$ for all $u\in {{\,\textrm{child}\,}}_G(v)$.

### Proof

Let *G* be a DAG on *X* and $A\subseteq X$ nonempty. By definition, if $v\in \operatorname {LCA}_G(A)$, then *v* is an ancestor of every vertex in *A* i.e. $A\subseteq {{\,\mathrm{\texttt{C}}\,}}_G(v)$ and no descendant of *v* is an ancestor of every vertex in *A*. Hence, for all $u\in V$ with $u\prec _G v$ at least one vertex in *A* is not contained in ${{\,\mathrm{\texttt{C}}\,}}_G(u)$, which implies that $A\not \subseteq {{\,\mathrm{\texttt{C}}\,}}_G(u)$. Hence, (1) implies (3). Trivially, (3) implies (2). Now, suppose that Statement (2) is satisfied. Since *A* is not empty and $A\subseteq {{\,\mathrm{\texttt{C}}\,}}_G(v)$, *v* is a common ancestor of every vertex in *A*. Moreover, $A\not \subseteq {{\,\mathrm{\texttt{C}}\,}}_G(u)$ for all $u\in {{\,\textrm{child}\,}}_G(v)$ implies together with Lemma 2.1 that $A\not \subseteq {{\,\mathrm{\texttt{C}}\,}}_G(w)$ for all descendants *w* of *v*. Hence, *v* is a least common ancestor of the vertices in *A*, i.e., $v\in \operatorname {LCA}_G(A)$. Thus, (2) implies (1).

Suppose now that $v\in \operatorname {LCA}_G(A)$ for some non-empty $A\subseteq X$. Thus, $A\subseteq {{\,\mathrm{\texttt{C}}\,}}_G(v)$. If *v* has no children, then the statement is vacuously true. Hence, assume that *v* is an inner vertex. By statement (2), $A\not \subseteq {{\,\mathrm{\texttt{C}}\,}}_G(u)$ and, therefore, ${{\,\mathrm{\texttt{C}}\,}}_G(u)\ne {{\,\mathrm{\texttt{C}}\,}}_G(v)$ for all $u\in {{\,\textrm{child}\,}}_G(v)$. $\square$

We will, in particular, be interested in situations where the LCA of certain sets of leaves is uniquely defined. More precisely, we are interested in DAGs where $|\operatorname {LCA}_G(A)|=1$ holds for certain subsets $A\subseteq X$. For simplicity, we will write $\operatorname {lca}_G(A)=v$ in case that $\operatorname {LCA}_G(A)=\{v\}$ and say that $\operatorname {lca}_G(A)$* is well-defined*; otherwise, we leave $\operatorname {lca}_G(A)$ *undefined*.

### Definition 3.2

(${{\,\mathrm{\textit{k}-LCA}\,}}$ and ${{\,\mathrm{\textit{k}-lca}\,}}$ vertices) Let *G* be a DAG on *X*, $k\ge 1$ be an integer and $v\in V(G)$.

1. The vertex *v* is a ${{\,\mathrm{\textit{k}-LCA}\,}}$* vertex* if $v\in \operatorname {LCA}_G(A)$ for some subset $A\subseteq X$ of size $|A|=k$.
2. The vertex *v* is a ${{\,\mathrm{\textit{k}-lca}\,}}$* vertex* if $v = \operatorname {lca}_G(A)$ for some subset $A\subseteq X$ of size $|A|=k$.

For a subset $\mathscr {I}\subseteq \{1,\dots ,|X|\}$, the vertex *v* is an $\mathscr {I}$-$\operatorname {LCA}$* vertex* (resp., $\mathscr {I}$-$\operatorname {lca}$ vertex) if it is a ${{\,\mathrm{\textit{k}-LCA}\,}}$ vertex (resp., ${{\,\mathrm{\textit{k}-lca}\,}}$ vertex) for some $k\in \mathscr {I}$.

**Fig. 3:**
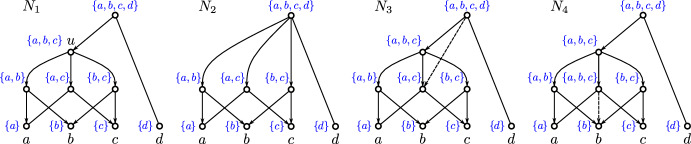
Shown are four phylogenetic networks $N_1$, $N_2$, $N_3$ and $N_4$ with the same set of leaves. Here, $N_1$ and $N_2 =N_1\ominus u$ are regular networks. The networks $N_3$ and $N_4$ only differ from $N_1$ by one edge each, as highlighted by dashed lines. Each inner vertex *v* of these networks with ${{\,\mathrm{\texttt{C}}\,}}_{N_i}(v)\ne \{a,b,c\}$ is a 2-$\operatorname {lca}$-vertex. In $N_1$, the vertex *u* with cluster ${{\,\mathrm{\texttt{C}}\,}}_{N_1}(u)=\{a,b,c\}$ is not a 2-$\operatorname {lca}$ vertex, but a 3-$\operatorname {lca}$ vertex. Consequently, $N_1$ is a $\{1,2,3\}$-$\operatorname {lca}$-Rel network but not $\{1,2\}$-$\operatorname {lca}$-Rel. One may also verify that the same holds for the network $N_3$ but that $N_2$ is $\{1,2\}$-$\operatorname {lca}$-Rel. For $N_4$ we can apply Lemma 3.4 to the edge ($u,u'$) connecting the vertices *u* and $u'$ for which ${{\,\mathrm{\texttt{C}}\,}}_{N_4}(u)=\{a,b,c\}={{\,\mathrm{\texttt{C}}\,}}_{N_4}(u')$ holds and conclude that $N_4$ is not $\operatorname {LCA}$-Rel and, therefore, not $\operatorname {lca}$-Rel. In particular, the vertex *u* in $N_4$ is not the LCA of any subset of leaves (Color figure online)

By Lemma 3.1, a vertex *v* can neither be a *k*-$\operatorname {LCA}$ nor a *k*-$\operatorname {lca}$ vertex whenever $k>|{{\,\mathrm{\texttt{C}}\,}}(v)|$. A less obvious relationship between *k*-$\operatorname {LCA}$ and $\ell$-$\operatorname {LCA}$ vertices (respectively *k*-$\operatorname {lca}$ and $\ell$-$\operatorname {lca}$ vertices) is captured by the following lemma.

### Lemma 3.3

If *v* is a *k*-$\operatorname {LCA}$ vertex of a DAG *G* for some $k\ge 1$, then *v* is an $\ell$-$\operatorname {LCA}$ vertex of *G* for all $\ell$ with $k\le \ell \le |{{\,\mathrm{\texttt{C}}\,}}_G(v)|$. If *v* is a *k*-$\operatorname {lca}$ vertex of a DAG *G* for some $k\ge 1$, then *v* is an $\ell$-$\operatorname {lca}$ vertex of *G* for all $\ell$ with $k\le \ell \le |{{\,\mathrm{\texttt{C}}\,}}_G(v)|$.

### Proof

Let *G* be a DAG on *X*. It is an easy task to verify that a vertex is a 1-$\operatorname {lca}$ vertex if and only if it is a leaf which, in turn, happens if and only if it is a 1-$\operatorname {LCA}$ vertex. Since, for a leaf $x\in X$ we have ${{\,\mathrm{\texttt{C}}\,}}_G(x)=\{x\}$, the two statements are trivial for the case when $k=1$.

Suppose now that *v* is a *k*-$\operatorname {LCA}$ vertex of *G* for some $k\ge 2$. Hence, there is some set $A\subseteq X$ of size $|A|=k\ge 2$ such that $v\in \operatorname {LCA}_G(A)$. By Lemma 3.1, $A\subseteq {{\,\mathrm{\texttt{C}}\,}}_G(v)$ and $A\not \subseteq {{\,\mathrm{\texttt{C}}\,}}_G(u)$ for all children *u* of *v*. Clearly, the latter property remains for all subsets $A'\subseteq {{\,\mathrm{\texttt{C}}\,}}_G(v)$ with $A\subseteq A'$. By Lemma 3.1, $v\in \operatorname {LCA}_G(A')$ for all such $A'\subseteq {{\,\mathrm{\texttt{C}}\,}}_G(v)$ with $A\subseteq A'$. Therefore, *v* is a $\ell$-$\operatorname {LCA}$ vertex of *G*, $k\le \ell \le |{{\,\mathrm{\texttt{C}}\,}}_G(v)|$.

Suppose now that *v* is a *k*-$\operatorname {lca}$ vertex. Hence, there is some some set $A\subseteq X$ of size $|A|=k$ such that $v = \operatorname {lca}_G(A)$ and, therefore, $\operatorname {LCA}_G(A)=\{v\}$. Let $A'\subseteq X$ be such that $A\subseteq A'\subseteq {{\,\mathrm{\texttt{C}}\,}}_G(v)$. We show that $\operatorname {LCA}_G(A')=\{v\}$. Since $A'\subseteq {{\,\mathrm{\texttt{C}}\,}}_G(v)$, *v* is a common ancestor of $A'$. Hence, there is a vertex $w\preceq _G v$ that is a least common ancestor of $A'$. Since $A\subseteq A'$, this vertex *w* is also a common ancestor of *A*. But this implies that $w\prec _G v$ is not possible since $v=\operatorname {lca}_G(A)$. Hence, $w=v$ must hold, i.e., $v\in \operatorname {LCA}_G(A')$. Assume, for contradiction, that there exists some $u\in \operatorname {LCA}_G(A')$ such that $u\ne v$. Note that *u* and *v* must be $\preceq _G$-incomparable. Since $A\subseteq A'$ and $u\in \operatorname {LCA}_G(A')$, the vertex *u* is in particular a common ancestor of the vertices in *A*. This, together with $\operatorname {lca}_G(A)=v$, means $v\preceq _G u$; a contradiction. Consequently, $\operatorname {LCA}_G(A') =\{v\}$. Hence, $v=\operatorname {lca}_G(A')$ must hold for all $A\subseteq A'\subseteq {{\,\mathrm{\texttt{C}}\,}}_G(v)$. In summary, *v* is a $\ell$-$\operatorname {lca}$ vertex of *G*, $k\le \ell \le |{{\,\mathrm{\texttt{C}}\,}}_G(v)|$. $\square$

Generally, the converse of Lemma 3.3 is not satisfied. Consider, for example, the vertex *u* of the network $N_1$ that satisfies ${{\,\mathrm{\texttt{C}}\,}}_{N_1}(u)=\{a,b,c\}$ in Fig. 3, which is a 3-$\operatorname {lca}$ vertex (thus, in particular, a 3-$\operatorname {LCA}$ vertex), but neither a 2-$\operatorname {lca}$ vertex nor a 2-$\operatorname {LCA}$ vertex.

The following two results provide a characterization of vertices that are not $\{1,\dots ,|X|\}$-$\operatorname {LCA}$, resp., not $\{1,\dots ,|X|\}$-$\operatorname {lca}$ vertices. These result will be employed in Sect. 5 to efficiently transform a given DAG *G* into a DAG $G'$ in which all vertices are ${{\,\mathrm{\textit{k}-LCA}\,}}$ or ${{\,\mathrm{\textit{k}-lca}\,}}$ vertices for at least one $k\in \{1,\dots ,|X|\}$.

### Lemma 3.4

For a DAG *G* on *X* and a vertex $v\in V(G)$, the following statements are equivalent.

1. *v* is not a ${{\,\mathrm{\textit{k}-LCA}\,}}$ vertex for any $k\in \{1,\dots ,|X|\}$.
2. there is a child *u* of *v* in *G* such that ${{\,\mathrm{\texttt{C}}\,}}_G(u)={{\,\mathrm{\texttt{C}}\,}}_G(v)$.
3. $$v\notin \operatorname {LCA}_G({{\,\mathrm{\texttt{C}}\,}}_G(v))$$

### Proof

Let *G* be a DAG on *X*, $v\in V(G)$ and put $C:={{\,\mathrm{\texttt{C}}\,}}_G(v)$. If *v* is not a ${{\,\mathrm{\textit{k}-LCA}\,}}$ vertex for any $k\in \{1,\dots ,|X|\}$, then in particular, $v\notin \operatorname {LCA}_G(C)$. By Lemma 3.1, there must be a child *u* of *v* such that $C\subseteq {{\,\mathrm{\texttt{C}}\,}}_G(u)$. Since $u\prec _G v$, Lemma 2.1 implies ${{\,\mathrm{\texttt{C}}\,}}_G(u)\subseteq {{\,\mathrm{\texttt{C}}\,}}_G(v)=C$ and thus, ${{\,\mathrm{\texttt{C}}\,}}_G(u)=C$. Hence, (1) implies (2). If there is a child *u* of *v* in *G* such that ${{\,\mathrm{\texttt{C}}\,}}_G(u)=C$, then $u\prec _G v$ implies that $v\notin \operatorname {LCA}_G(C)$, i.e., (2) implies (3). Assume now that $v\notin \operatorname {LCA}_G(C)$ and put $\ell :=|C|$. Clearly, *v* is not a ${{\,\mathrm{\textit{k}-LCA}\,}}$ vertex for any $A\subseteq X$ of size $|A|>\ell$ as, in this case, $A\not \subseteq C$. Moreover, since *v* is not an $\ell$-$\operatorname {LCA}$ vertex, contraposition of Lemma 3.3 implies that *v* is not a ${{\,\mathrm{\textit{k}-lca}\,}}$ vertex for any $k\in \{1,\dots ,\ell \}$. Thus, (3) implies (1). $\square$

### Lemma 3.5

For a DAG *G* on *X* and a vertex $v\in V(G)$, the following statements are equivalent.

1. *v* is not a ${{\,\mathrm{\textit{k}-lca}\,}}$ vertex for any $k\in \{1,\dots ,|X|\}$.
2. there is a child *u* of *v* in *G* such that ${{\,\mathrm{\texttt{C}}\,}}_G(u)={{\,\mathrm{\texttt{C}}\,}}_G(v)$ or $|\operatorname {LCA}_G({{\,\mathrm{\texttt{C}}\,}}_G(v))|\ge 2$.
3. $$v\ne \operatorname {lca}_G({{\,\mathrm{\texttt{C}}\,}}_G(v))$$.

### Proof

Let *G* be a DAG on *X*, $v\in V(G)$ and put $C:={{\,\mathrm{\texttt{C}}\,}}_G(v)$. We start with showing that (1) implies (2). Suppose that *v* is not a ${{\,\mathrm{\textit{k}-lca}\,}}$ vertex for any $k\in \{1,\dots ,|X|\}$, then in particular, $v\ne \operatorname {lca}_G(C)$. Thus, if $v\in \operatorname {LCA}_G(C)$, then $|\operatorname {LCA}_G(C)|\ge 2$. If $v\not \in \operatorname {LCA}_G(C)$, then Lemma 3.1 implies that there must be a child *u* of *v* such that $C\subseteq {{\,\mathrm{\texttt{C}}\,}}_G(u)$. Since $u\prec _G v$, Lemma 2.1 implies ${{\,\mathrm{\texttt{C}}\,}}_G(u)\subseteq C$ and thus, ${{\,\mathrm{\texttt{C}}\,}}_G(u)=C$. Thus, (1) implies (2). Assume now that statement (2) holds. If $|\operatorname {LCA}_G(C)|\ge 2$, then in particular $v\ne \operatorname {lca}_G(C)$. If there is a child *u* of *v* in *G* such that ${{\,\mathrm{\texttt{C}}\,}}_G(u)=C$, then Lemma 3.4 implies that $v\notin \operatorname {LCA}_G(C)$ and thus, $v\ne \operatorname {lca}_G(C)$. Hence, (2) implies (3). Finally, suppose that $v\ne \operatorname {lca}_G(C)$. Hence, *v* is not an $\ell$-$\operatorname {lca}$ vertex for $\ell =|C|$. Contraposition of Lemma 3.3 implies that *v* is not a ${{\,\mathrm{\textit{k}-lca}\,}}$ vertex for any $k\in \{1,\dots ,\ell \}$. Clearly, *v* is not an ${{\,\mathrm{\textit{k}-lca}\,}}$ vertex for any $A\subseteq X$ of size $|A|>\ell$ as, in this case, $A\not \subseteq C$. Consequently, (3) implies (1). $\square$

Lemma 3.4 and 3.5 will be useful in both Sect. 4 and Sect. 5. The contrapositive of statements (1) and (3) in these lemmas together with the fact that *v* can only be a ${{\,\mathrm{\textit{k}-lca}\,}}$ or ${{\,\mathrm{\textit{k}-LCA}\,}}$ vertex if $k\le |{{\,\mathrm{\texttt{C}}\,}}_G(v)|$ imply

### Corollary 3.6

Let *G* be a DAG on *X*, $v\in V(G)$. Then, *v* is a ${{\,\mathrm{\textit{k}-LCA}\,}}$ vertex in *G* for some *k* if and only if $v\in \operatorname {LCA}_G({{\,\mathrm{\texttt{C}}\,}}_G(v))$. Moreover, *v* is a ${{\,\mathrm{\textit{k}-lca}\,}}$ vertex in *G* for some *k* if and only if $v=\operatorname {lca}_G({{\,\mathrm{\texttt{C}}\,}}_G(v))$. In both cases, $k\le |{{\,\mathrm{\texttt{C}}\,}}_G(v)|$.

In what follows, we consider DAGs for which each vertex *v* satisfies $v\in \operatorname {LCA}(A)$ or $v = \operatorname {lca}(A)$ for some set *A* whose size $k=|A|$ is contained in a specified set $\mathscr {I}$ of integers.

### Definition 3.7

Let *G* be a DAG on *X*, $v\in V(G)$ and $\mathscr {I}$ be a set of integers.

1. *G* is $\mathscr {I}$-$\operatorname {lca}$-*relevant* (in short $\mathscr {I}$-$\operatorname {lca}$-$\textsc {Rel}$) if all vertices in *V*(*G*) are $\mathscr {I}$-$\operatorname {lca}$ vertices. DAGs that are $\{1,2,\dots ,|X|\}$-$\operatorname {lca}$-Rel are simply called $\operatorname {lca}$-Rel.
2. *G* is $\mathscr {I}$-$\operatorname {LCA}$-*relevant* (in short $\mathscr {I}$-$\operatorname {LCA}$-$\textsc {Rel}$) if all vertices in *V*(*G*) are $\mathscr {I}$-$\operatorname {LCA}$ vertices. DAGs that are $\{1,2,\dots ,|X|\}$-$\operatorname {LCA}$-Rel are simply called $\operatorname {LCA}$-Rel.

Thus, *G* is $\operatorname {LCA}$-Rel if each vertex *v* in *G* is a least common ancestor for at least some set $A\subseteq X$. Similarity, *G* is $\operatorname {lca}$-Rel if, for all $v\in V(G)$, there is some set $A\subseteq X$ such that $v= \operatorname {lca}_G(A)$.

### Observation 3.8

Every $\mathscr {I}$-$\operatorname {lca}$-$\textsc {Rel}$ DAG is $\operatorname {lca}$-Rel and $\operatorname {LCA}$-Rel. Every $\mathscr {I}$-$\operatorname {LCA}$-$\textsc {Rel}$ DAG is $\operatorname {LCA}$-Rel.

There are DAGs and even networks that are not $\mathscr {I}$-$\operatorname {lca}$-Rel for any $\mathscr {I}\subseteq \{1,\dots , |X|\}$, see the network $N_4$ in Fig. 3. In contrast, for every set $\mathscr {I} \subseteq \{1,\dots ,n\}$ with $1\in \mathscr {I}$, there is an $\mathscr {I}$-$\operatorname {lca}$-$\textsc {Rel}$ DAG *G*. To see this, let $\mathscr {I} = \{i_1,\dots , i_\ell \}$ with $1=i_1<i_2<\dots <i_\ell$ and take the Hasse diagram of the clustering system $\{\{j\}\mid 1\le j\le i_\ell \} \cup \{\{1,\dots ,j\} \mid j\in \{i_2,\dots ,i_\ell \}\}$ which is $\mathscr {I}$-$\operatorname {lca}$-$\textsc {Rel}$.

The requirement that $1\in \mathscr {I}$ is indispensable for $\mathscr {I}$-$\operatorname {lca}$-$\textsc {Rel}$ DAGs. To see this, observe that every leaf $x \in L(G)$ of a DAG *G* satisfies $\operatorname {LCA}_G(\{x\}) = \{x\}$ and that *x* cannot be an ancestor of any vertex $y\ne x$. Hence, a leaf $x \in L(G)$ is always a 1-$\operatorname {lca}$ vertex but never a *k*-$\operatorname {lca}$ vertex for $k>1$. Since $L(G)\ne \emptyset$ for all DAGs *G*, every DAG contains at least one 1-$\operatorname {lca}$ vertex and thus, at least one 1-$\operatorname {LCA}$ vertex. Hence, if *all* vertices are $\mathscr {I}$-$\operatorname {LCA}$ or $\mathscr {I}$-$\operatorname {lca}$ vertices, then $1\in \mathscr {I}$ must hold. The latter is captured by the following

### Definition 3.9

For a given DAG *G*, the set $\mathscr {I}^{1}$ always denotes a subset of $\{1,\dots , |L(G)|\}$ that satisfies $1\in \mathscr {I}^{1}$.

A useful structural property of $\operatorname {LCA}$-$\textsc {Rel}$ and $\operatorname {lca}$-$\textsc {Rel}$ DAGs is provided next.

### Lemma 3.10

An $\operatorname {LCA}$-$\textsc {Rel}$ or $\operatorname {lca}$-$\textsc {Rel}$ DAG does not contain vertices *w* with ${{\,\textrm{outdeg}\,}}_G(w)=1$ and is, thus, phylogenetic.

### Proof

Let *G* be an $\operatorname {LCA}$-$\textsc {Rel}$ DAG. Contraposition of Lemma 3.4 shows that *G* cannot contain vertices *w* that have a child *u* such that ${{\,\mathrm{\texttt{C}}\,}}_G(w) = {{\,\mathrm{\texttt{C}}\,}}_G(u)$. Hence, *G* can, in particular, not have any vertex with a single child, i.e., ${{\,\textrm{outdeg}\,}}_G(w)\ne 1$ for all $w\in V(G)$. Thus, *G* is phylogenetic. Since every $\operatorname {lca}$-$\textsc {Rel}$ DAG is, in particular, $\operatorname {LCA}$-$\textsc {Rel}$ the statement holds for $\operatorname {lca}$-$\textsc {Rel}$ DAGs as well. $\square$

We finally show that the set $\operatorname {LCA}_G(A)$ in a DAG $G = (V, E)$ can be determined in $O((|V|+|E|)|A|)$ time. Assuming that the size of *A* is treated as constant, i.e., $|A|\in O(1)$, this result implies that $\operatorname {LCA}_G(A)$ can be determined in linear time. To achieve this goal, we use a *topological order* $\ll$ on *V*, i.e., a total order on the vertices in *G* such that $(u,v) \in E$ implies $v\ll u$. Since we consider DAGs, such an order always exists (Cormen et al. 2022). The pseudocode of the underlying algorithm is provided in Algorithm 1. The main idea of this algorithm is as follows: we determine the set $A\setminus {{\,\mathrm{\texttt{C}}\,}}_G(v_i)$, which is stored in *C*[*i*], for each $v_i\in V$. We then employ Lemma 3.1 which states that $v_i\in \operatorname {LCA}_G(A)$ if and only if $A\subseteq {{\,\mathrm{\texttt{C}}\,}}_G(v_i)$ and $A\not \subseteq {{\,\mathrm{\texttt{C}}\,}}_G(v_j)$ for all $v_j\in {{\,\textrm{child}\,}}_G(v_i)$ which, in turn, is precisely if $C[i] = \emptyset$ and $C[j] \ne \emptyset$ for all *j* such that $v_j\in {{\,\textrm{child}\,}}_G(v_i)$ (see Line 12 in Algorithm 1).

**Algorithm 1:**
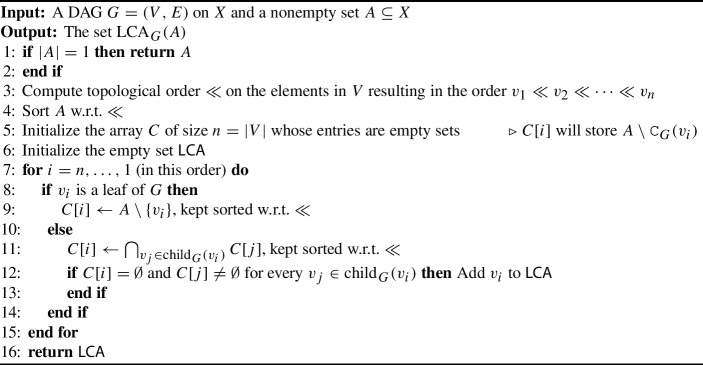
Find_$$\operatorname {LCA}$$_of_set_*A*

### Proposition 3.11

For a given DAG $G=(V,E)$ on *X* and a non-empty set $A\subseteq X$, Algorithm 1 correctly determines $\operatorname {LCA}_G(A)$. Moreover, Algorithm 1 can be implemented to run in $O((|V|+|E|)|A|)$ time.

### Proof

Let the DAG $G=(V,E)$ on *X* and the non-empty set $A\subseteq X$ serve as an input for Algorithm 1. For simplicity, put $\operatorname {LCA}(\cdot ) :=\operatorname {LCA}_G(\cdot )$. We start with proving the correctness of Algorithm 1. In Line 1, we first check if $|A|=1$ and, in the affirmative case, $\operatorname {LCA}(A)=A$ must hold and the algorithm correctly returns *A*. Otherwise, if $|A|>1$, the algorithm continues as follows. In Line 3, the vertices in *V* are topologically ordered resulting in $v_1 \ll v_2 \ll \dots \ll v_n$, where $n:=|V|$. Sorting *A* in Line 4 and maintaining sorted elements in Lines 9 and 11 are primarily used to establish the runtime but do not influence the correctness proof. Thus, we can treat the set *A* and the array *C* as unordered for now. The array *C* and the empty set $\textsf{LCA}$ is initialized in Line 5 and 6, respectively. The entry *C*[*i*] will store the elements of $A\setminus {{\,\mathrm{\texttt{C}}\,}}_G(v_i)$. The main idea of this algorithm is based on Lemma 3.1 which states that $v_i\in \operatorname {LCA}(A)$ precisely if $A\subseteq {{\,\mathrm{\texttt{C}}\,}}_G(v_i)$ and $A\not \subseteq {{\,\mathrm{\texttt{C}}\,}}_G(v_j)$ for all $v_j\in {{\,\textrm{child}\,}}_G(v_i)$. The latter is precisely if $C[i] = \emptyset$ and $C[j] \ne \emptyset$ for all *j* that correspond to indices of the children $v_j$ of $v_i$. It thus suffices to show that *C*[*i*] is correctly determined for all $i\in \{1,\dots , n\}$. In the *for*-loop in Line 7 the vertices are processed in the order $v_n, v_{n-1},\dots v_1$. Based on the topological order, this ensures that, whenever $v_i$ is processed, all its descendants have been processed as they must be located in $v_{i+1},\dots ,v_n$. If $v_i$ is a leaf of *G*, then ${{\,\mathrm{\texttt{C}}\,}}_G(v_i) =\{v_i\}$ holds. Hence, $C[i]=A\setminus {{\,\mathrm{\texttt{C}}\,}}_G(v_i)=A\setminus \{v_i\}$ is correctly determined in Line 9. Otherwise, i.e. if $v_i$ is an inner vertex, then we put $C[i] = \cap _{v_j\in {{\,\textrm{child}\,}}_G(v_i)} C[j]$ in Line 11. By the latter arguments and induction, for each such $v_j$ the set *C*[*j*] has already been correctly determined. This and Lemma 2.2 implies that $C[i]=\cap _{v_j\in {{\,\textrm{child}\,}}_G(v_i)}A\setminus {{\,\mathrm{\texttt{C}}\,}}_G(v_j)=A\setminus {{\,\mathrm{\texttt{C}}\,}}_G(v_i)$ is correctly determined. In Line 12, we simply verify if the conditions of Lemma 3.1 are satisfied and, in the affirmative case, $v_i$ is correctly added to $\textsf{LCA}$. In summary, $\operatorname {LCA}(A)$ is correctly determined.

Let us now consider the runtime of Algorithm 1. To this end, we assume that $n=|V|$, $m=|E|$ and $k=|A|$. Line 1 takes constant time. Determining the topological order $\ll$ of *V* can be done in $O(n+m)$ time and sorting *A* in Line 4 can be done in $O(k\log {k})$ time (Cormen et al. 2022). The tasks in Line 5-6 can be accomplished in *O*(*n*) time. We may assume that the DAG *G* is represented as an adjacency list *L*. In this case, we can traverse all entries of *L* and check whether the entry $L[v_i]$ is empty (resp. non-empty) in which case $v_i$ is a leaf (resp. inner vertex). As a pre-processing step this takes *O*(*n*) time and we can, afterwards, check in constant time as whether $v_i$ is a leaf or not. Thus, the *if*-condition in Line 8 can be evaluated in constant time. In Line 9, we can traverse the sorted set *A*, adding all elements except $v_i$ to the ordered set *C*[*i*] in *O*(*k*) time, keeping the order of the elements. Since this is repeated for the |*X*| leaves of *G*, Line 9 contributes with *O*(*k*|*X*|) over all iterations. In Line 11, we compute the intersection of ordered sets and keep the order. The intersection of two sorted sets *S* and $S'$ resulting in a sorted set can be done in $O(|S|+|S'|)$ time (Aho et al. 1983). Each set *C*[*j*] has *O*(*k*) elements, as they are subsets of *A*. Thus, computing the $\ll$-sorted set *C*[*i*] as the intersection $\bigcap _{v_j\in {{\,\textrm{child}\,}}_G(v_i)} C[j]$ can be done $O(|{{\,\textrm{child}\,}}_G(v_i)|k)$ time. As the latter task is repeated for all inner vertices of *G*, the total runtime, for Line 11 is $O(\sum _{i=1}^n (|{{\,\textrm{child}\,}}_G(v_i)| k)) = O(mk)$ time. In Line 12, we simply make $|{{\,\textrm{child}\,}}_G(v_i)|+1$ constant time look-ups in the array *C* to determine whether the ordered sets are empty or not. Once again summing over all inner vertices, this contributes with *O*(*m*) to the total runtime.

In summary, the total runtime of Algorithm 1 is in $O(n+m+k\log {k}+k|X|+mk)$ time. Since $\log {k}<k\le |X|$ and $|X|\le n$, the terms *k*|*X*| and $k\log {k}$ are both dominated by the term *nk*. Thus, the total runtime simplifies to $O(nk+mk)$. $\square$

Since $\operatorname {LCA}_G(A)$ can be determined for $G=(V,E)$ in $O((|V|+|E|)|A|)$ time and since $|\operatorname {LCA}_G(A)| \in O(|V(G))|$, the additional costs for checking if $v\in \operatorname {LCA}_G(A)$ or $v=\operatorname {lca}_G(A)$ add *O*(|*V*|) to the cost of computing $\operatorname {LCA}_G(A)$. Moreover, by Corollary 3.6, a DAG is $\operatorname {LCA}$-Rel, resp., $\operatorname {lca}$-Rel if and only if, for all of its vertices *v*, it holds that $v\in \operatorname {LCA}_G({{\,\mathrm{\texttt{C}}\,}}_G(v))$, resp., $v=\operatorname {lca}_G({{\,\mathrm{\texttt{C}}\,}}_G(v))$. Summarizing the latter arguments, we obtain

### Corollary 3.12

For a given DAG $G=(V,E)$ on *X*, a non-empty set $A\subseteq X$ and a vertex $v\in V$, it can be determined in $O((|V|+|E|)|A|)$ time if $v\in \operatorname {LCA}_G(A)$ and if $v=\operatorname {lca}_G(A)$. Moreover, it can decided in polynomial time if *G* is $\operatorname {LCA}$-Rel (resp., $\operatorname {lca}$-Rel) or not.

## Characterization of lca- & LCA-Relevant DAGs and Regular DAGs

By Corollary 3.6, every ${{\,\mathrm{\textit{k}-lca}\,}}$ vertex *v* and therefore, every vertex *v* in an $\mathscr {I}^{1}$-$\operatorname {lca}$-Rel DAG, satisfies $v = \operatorname {lca}_G({{\,\mathrm{\texttt{C}}\,}}_G(v))$. To cover such type of DAGs we provide

### Definition 4.1

A DAG *G* satisfies the *cluster-lca (CL)* property if $\operatorname {lca}_G({{\,\mathrm{\texttt{C}}\,}}_G(v))$ is well-defined for all $v\in V(G)$. A DAG *G* has the *strong cluster-lca (strong-(CL))* property if $v = \operatorname {lca}_G({{\,\mathrm{\texttt{C}}\,}}_G(v))$ for all $v\in V(G)$.

**Fig. 4:**
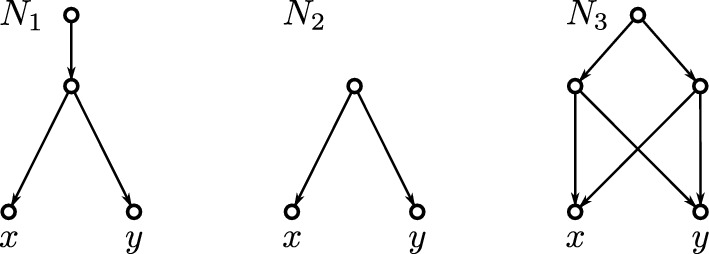
Shown are three networks $N_1$, $N_2$ and $N_3$ having the same clustering system $\mathfrak {C} = \{\{x\}, \{y\}, \{x,y\}\}$. The network $N_1$ has the (CL) but not the strong-(CL) property. The network $N_2$ has the strong-(CL) and, thus, also the (CL) property. The network $N_3$ has neither the strong-(CL) nor the (CL) property

By definition, strong-(CL) implies (CL). However, there are DAGs with the (CL) property but without the strong-(CL) property and DAGs without (CL) property, see Fig. 4 for an example.

### Lemma 4.2

If a DAG *G* satisfies (PCC) then it satisfies (CL). If *G* satisfies (CL), then $\operatorname {lca}_G( {{\,\mathrm{\texttt{C}}\,}}_G (v)) \preceq _G v$ and ${{\,\mathrm{\texttt{C}}\,}}( \operatorname {lca}_G ({{\,\mathrm{\texttt{C}}\,}}_G (v))) = {{\,\mathrm{\texttt{C}}\,}}_G (v)$ for all $v\in V(G)$.

### Proof

We emphasize first that these results have been proven for the case that *G* is a network, cf. (Hellmuth et al. (2023), Lem. 36 & 38). Suppose that *G* is DAG on *X* that is not a network and thus, $|R(G)|>1$. Let *N* be the network obtained from *G* by adding a new root $\rho$ to *G* and edges $(\rho ,r)$ for all $r\in R(G)$. By construction $V(G) = V(N)\setminus \{\rho \}$, ${{\,\mathrm{\texttt{C}}\,}}_N(\rho )=X$ and ${{\,\mathrm{\texttt{C}}\,}}_N(v) = {{\,\mathrm{\texttt{C}}\,}}_G(v)$ for all $v\in V(G)$. Assume first that *G* that satisfies (PCC). It is straightforward to verify that *N* satisfies (PCC). Therefore, *N* satisfies (CL). One easily observes that, for all $v\in V(G)$, we have $\operatorname {lca}_N ({{\,\mathrm{\texttt{C}}\,}}_N (v))=\operatorname {lca}_G ({{\,\mathrm{\texttt{C}}\,}}_G (v))$. Since *N* satisfies (CL), *G* satisfies (CL). Moreover, since the statements are true for *N* and since $\operatorname {lca}_N ({{\,\mathrm{\texttt{C}}\,}}_N (v))=\operatorname {lca}_G ({{\,\mathrm{\texttt{C}}\,}}_G (v))$, we can conclude that the second statement is satisfied for the DAG *G*. $\square$

We provide now a simple characterization of DAGs with (CL) property.

### Proposition 4.3

A DAG $G = (V,E)$ has the (CL) property if and only if, for every vertex $v\in V$, $v = \operatorname {lca}_G({{\,\mathrm{\texttt{C}}\,}}_G(v))$ or *v* has a child *u* such that ${{\,\mathrm{\texttt{C}}\,}}_G(v)={{\,\mathrm{\texttt{C}}\,}}_G(u)$.

### Proof

Let $G = (V,E)$ be DAG and $v\in V$. Suppose that *G* has the (CL) property. If $v = \operatorname {lca}_G({{\,\mathrm{\texttt{C}}\,}}_G(v))$, then we are done. Hence, assume that $v \ne \operatorname {lca}_G({{\,\mathrm{\texttt{C}}\,}}_G(v))$. Then, Lemma 3.5 implies that there either is a child *u* of *v* in *G* with ${{\,\mathrm{\texttt{C}}\,}}_G(v)={{\,\mathrm{\texttt{C}}\,}}_G(u)$, or $|\operatorname {LCA}_G({{\,\mathrm{\texttt{C}}\,}}_G(v))|\ge 2$. However, the latter cannot hold since *G* has the (CL) property, which establishes the *only if*-direction.

Conversely, assume that every vertex $v\in V$ satisfies: (a) $v = \operatorname {lca}_G({{\,\mathrm{\texttt{C}}\,}}_G(v))$ or (b) *v* has a child *u* such that ${{\,\mathrm{\texttt{C}}\,}}_G(v)={{\,\mathrm{\texttt{C}}\,}}_G(u)$. If $v = \operatorname {lca}_G({{\,\mathrm{\texttt{C}}\,}}_G(v))$, then $\operatorname {lca}_G({{\,\mathrm{\texttt{C}}\,}}_G(v))$ is well-defined. Suppose that *v* has a child *u* such that ${{\,\mathrm{\texttt{C}}\,}}_G(v)={{\,\mathrm{\texttt{C}}\,}}_G(u)$. We can now take a $\preceq _G$-minimal vertex *w* that satisfies $w\preceq _G u$ and ${{\,\mathrm{\texttt{C}}\,}}_G(w)={{\,\mathrm{\texttt{C}}\,}}_G(v)$. If *w* is a leaf, then ${{\,\mathrm{\texttt{C}}\,}}_G(w)=\{w\}$ and we have $w=\operatorname {lca}_G({{\,\mathrm{\texttt{C}}\,}}_G(w))$ which implies that $\operatorname {lca}_G({{\,\mathrm{\texttt{C}}\,}}_G(w)) = \operatorname {lca}_G({{\,\mathrm{\texttt{C}}\,}}_G(v))$ is well-defined. Otherwise, *w* is an inner vertex. By choice of *w*, all children $u'$ of *w* must satisfy ${{\,\mathrm{\texttt{C}}\,}}_G(u')\ne {{\,\mathrm{\texttt{C}}\,}}_G(w)$, i.e., *w* does not satisfy (b) and must therefore, satisfy (a) i.e. that $w =\operatorname {lca}_G({{\,\mathrm{\texttt{C}}\,}}_G(w))$. This together with ${{\,\mathrm{\texttt{C}}\,}}_G(w)={{\,\mathrm{\texttt{C}}\,}}_G(v)$ implies that $\operatorname {lca}_G({{\,\mathrm{\texttt{C}}\,}}_G(v))$ is well-defined. In summary, *G* satisfies (CL). $\square$

As we shall see later, there is a close relationship between regular DAGs, DAGs that are $\operatorname {lca}$-$\textsc {Rel}$ and DAGs with the strong-(CL) property. Before considering $\operatorname {lca}$-$\textsc {Rel}$ DAGs, we provide a characterization of $\operatorname {LCA}$-$\textsc {Rel}$ DAGs that is an immediate consequence of Lemma 3.4 applied to all vertices.

### Theorem 4.4

A DAG *G* is $\operatorname {LCA}$-$\textsc {Rel}$ if and only if there are no adjacent vertices *u* and *v* in *G* that satisfy ${{\,\mathrm{\texttt{C}}\,}}_G(u)={{\,\mathrm{\texttt{C}}\,}}_G(v)$.

The more specific property of being $\operatorname {lca}$-$\textsc {Rel}$ imposes more structural constraints on the DAG in question which, in turn, allows us to provide the following characterization.

### Theorem 4.5

The following statements are equivalent for every DAG *G*.

1. *G* is $$\operatorname {lca}$$-Rel
2. *G* has the strong-(CL) property.
3. *G* has the (CL) property and is $\operatorname {LCA}$-Rel.
4. *G* satisfies (PCC) and is $\operatorname {LCA}$-Rel.
5. *G* satisfies (PCC) and $u\ne v$ implies ${{\,\mathrm{\texttt{C}}\,}}_G(u)\ne {{\,\mathrm{\texttt{C}}\,}}_G(v)$ for all $u,v\in V(G)$.
6. ${{\,\mathrm{\texttt{C}}\,}}_G(u)\subseteq {{\,\mathrm{\texttt{C}}\,}}_G(v)$ if and only if $u\preceq _G v$ for all $u,v\in V(G)$.

### Proof

By Corollary 3.6, Statements (1) and (2) are equivalent. Now, assume that Statement (3) holds. Let *v* be a vertex of *G*. Corollary 3.6 together with the fact that *v* is a *k*-$\operatorname {LCA}$ vertex for some *k* implies that $v\in \operatorname {LCA}_G({{\,\mathrm{\texttt{C}}\,}}_G(v))$. Since *G* has the (CL) property, we have $|\operatorname {LCA}_G({{\,\mathrm{\texttt{C}}\,}}_G(v))|=1$. The latter two arguments imply that $v=\operatorname {lca}_G({{\,\mathrm{\texttt{C}}\,}}_G(v))$. Since *v* was chosen arbitrarily, *G* is an $\operatorname {lca}$-Rel DAG, i.e., Statement (1) holds. Since the two equivalent Statements (1) and (2) together immediately imply Statement (3), we conclude that Statements (1), (2) and (3) are equivalent.

Therefore, it suffices to show that the following implications (2) $\Rightarrow$ (4) $\Rightarrow$ (5) $\Rightarrow$ (6) $\Rightarrow$ (2) hold. Assume that Condition (2) holds. Hence, *G* is a DAG that has the strong-(CL) property. To show (PCC), observe that, by Lemma 2.1, $u\preceq _G v$ implies ${{\,\mathrm{\texttt{C}}\,}}_G(u)\subseteq {{\,\mathrm{\texttt{C}}\,}}_G(v)$ for all $u,v\in V(G)$. Suppose now that $u,v\in V(G)$ are such that ${{\,\mathrm{\texttt{C}}\,}}_G(u)\subseteq {{\,\mathrm{\texttt{C}}\,}}_G(v)$. Since *G* satisfies strong-(CL), $u=\operatorname {lca}_G({{\,\mathrm{\texttt{C}}\,}}_G(u))$. This together with ${{\,\mathrm{\texttt{C}}\,}}_G(u)\subseteq {{\,\mathrm{\texttt{C}}\,}}_G(v)$ and the definition of $\operatorname {lca}$s implies that $u=\operatorname {lca}_G({{\,\mathrm{\texttt{C}}\,}}_G(u)) \preceq _G v$. Hence, *G* satisfies (PCC). In addition, *G* is $\operatorname {LCA}$-Rel since (2) and (3) are equivalent. In summary, (2) implies (4).

Assume that Condition (4) holds. Hence, *G* satisfies (PCC) and is $\operatorname {LCA}$-Rel. Since *G* satisfies (PCC), there cannot be any incomparable vertices *u*, *v* in *G* with ${{\,\mathrm{\texttt{C}}\,}}_G(v) = {{\,\mathrm{\texttt{C}}\,}}_G(u)$. Thus, any two incomparable vertices have distinct clusters. By Theorem 4.4, no adjacent vertices *u* and *v* in *G* can satisfy ${{\,\mathrm{\texttt{C}}\,}}_G(u)={{\,\mathrm{\texttt{C}}\,}}_G(v)$. This together with Lemma 2.1 implies that for any two vertices *u*, *v* in *G* with $u\prec _G v$ it holds that ${{\,\mathrm{\texttt{C}}\,}}_G(u)\subsetneq {{\,\mathrm{\texttt{C}}\,}}_G(v)$. In summary, $u\ne v$ implies ${{\,\mathrm{\texttt{C}}\,}}_G(u)\ne {{\,\mathrm{\texttt{C}}\,}}_G(v)$ for all $u,v\in V(G)$. Hence, (4) implies (5).

Assume that Condition (5) holds. Hence, *G* is a DAG that satisfies (PCC) and where $u\ne v$ implies ${{\,\mathrm{\texttt{C}}\,}}_G(u)\ne {{\,\mathrm{\texttt{C}}\,}}_G(v)$ for all $u,v\in V(G)$. Let $u,v\in V(G)$ be chosen arbitrarily. If $u\preceq _G v$, then Lemma 2.1 implies that ${{\,\mathrm{\texttt{C}}\,}}_G(u)\subseteq {{\,\mathrm{\texttt{C}}\,}}_G(v)$. Suppose now that ${{\,\mathrm{\texttt{C}}\,}}_G(u)\subseteq {{\,\mathrm{\texttt{C}}\,}}_G(v)$. Since *G* satisfies (PCC), *u* and *v* must be $\preceq _G$-comparable. However, the case $v\prec _G u$ cannot occur since then $u\ne v$ and, thus, ${{\,\mathrm{\texttt{C}}\,}}_G(u)\ne {{\,\mathrm{\texttt{C}}\,}}_G(v)$ which together with Lemma 2.1 implies that ${{\,\mathrm{\texttt{C}}\,}}_G(v)\subsetneq {{\,\mathrm{\texttt{C}}\,}}_G(u)$; a contradiction to ${{\,\mathrm{\texttt{C}}\,}}_G(u)\subseteq {{\,\mathrm{\texttt{C}}\,}}_G(v)$. Thus, $u\preceq _G v$ holds and (5) implies (6).

Assume that Condition (6) holds. Hence, *G* is a DAG such that ${{\,\mathrm{\texttt{C}}\,}}_G(u)\subseteq {{\,\mathrm{\texttt{C}}\,}}_G(v)$ if and only if $u\preceq _G v$ for all $u,v\in V(G)$. Thus, *G* satisfies (PCC). By Lemma 4.2, *G* satisfies (CL). Thus, $w = \operatorname {lca}_G({{\,\mathrm{\texttt{C}}\,}}_G(v))$ is well-defined for all $v\in V(G)$. Again, by Lemma 4.2, ${{\,\mathrm{\texttt{C}}\,}}_G (w) = {{\,\mathrm{\texttt{C}}\,}}_G (v)$. Thus, we have ${{\,\mathrm{\texttt{C}}\,}}_G (w) \subseteq {{\,\mathrm{\texttt{C}}\,}}_G (v)$ implying $w\preceq _G v$. In addition, ${{\,\mathrm{\texttt{C}}\,}}_G (v) \subseteq {{\,\mathrm{\texttt{C}}\,}}_G (w)$ implies $v\preceq _G w$. Consequently, $w=v$ holds. Therefore, $v = \operatorname {lca}_G({{\,\mathrm{\texttt{C}}\,}}_G(v))$ for all $v\in V(G)$ and *G* has the strong-(CL) property. In summary, (6) implies (2), which completes this proof. $\square$

We continue to establish a few further key results that enable us to show the close connection between regular DAGs and $\operatorname {lca}$-Rel DAGs in Theorem 4.10. Regular networks have very constrained structural properties, as characterized in (Hellmuth et al. (2023), Thm. 2). Here, we generalize these results to arbitrary DAGs.

### Theorem 4.6

The following statements are equivalent for every DAG *G*.

1. *G* is regular
2. *G* does not contain vertices with out-degree 1, is shortcut-free and satisfies (PCC).

In particular, a regular DAG is phylogenetic.

### Proof

If *G* is a network, then we can use (Hellmuth et al. (2023), Thm. 2) which states that (1) and (2) are equivalent for networks. If *G* is a DAG that is not a network, then $|R(G)|>1$. In this case, we can obtain a network $N_G$ from *G* by adding a new root $\rho$ to *G* and edges $(\rho ,r)$ for all $r\in R(G)$. It is now a straightforward task—which we leave to the reader—to verify that *G* satisfies (1) if and only if $N_G$ does and that *G* satisfies (2) if and only if $N_G$ does. Since (1) and (2) are equivalent for networks, the latter arguments show that (1) and (2) are equivalent for DAGs. In particular, since regular DAGs have no vertices of out-degree 1, they must be phylogenetic. $\square$

We note that DAGs that are shortcut-free and satisfy (PCC) are also known as *semi-regular* (Hellmuth et al. 2023). The following result generalizes (Hellmuth et al. (2023), Lem. 22) that has been established for networks.

### Lemma 4.7

For every set system $\mathfrak {C}$, the Hasse diagram $\mathscr {H}(\mathfrak {C})$ is a shortcut-free DAG that satisfies (PCC). Moreover, if $\mathfrak {C}$ is grounded, then $\mathscr {H}(\mathfrak {C})$ is regular and phylogenetic. Furthermore, if $\mathfrak {C}$ is a clustering system, then $\mathscr {H}(\mathfrak {C})$ is a regular network.

### Proof

The first statement is a direct consequence of the definition of the Hasse diagram. Hence, to prove that $\mathscr {H}:=\mathscr {H}(\mathfrak {C})$ is regular for grounded set systems $\mathfrak {C}$, it suffices to show that $\mathscr {H}$ has no vertex of out-degree 1 (cf. Theorem 4.6). For contradiction, assume that $\mathscr {H}$ has a vertex *C* such that ${{\,\textrm{outdeg}\,}}_{\mathscr {H}}(C)=1$. Let $C'$ be the unique child of *C*. Since $C,C'\in \mathfrak {C}$ are distinct clusters with $C'\subsetneq C$ and $C'\ne \emptyset$, there is some element $x\in C\setminus C'$. Since $\mathfrak {C}$ is grounded, $\{x\}\in \mathfrak {C}$. But then the definition of $\mathscr {H}$ together with $\{x\}\subseteq C$ and $\{x\}\not \subseteq C'$ implies $\{x\}\prec _{\mathscr {H}} C$ while $\{x\}$ and $C'$ are $\preceq _{\mathscr {H}}$-incomparable. One easily verifies that this implies that *C* must have at least two children; a contradiction. Thus, $\mathscr {H}$ is regular. By Theorem 4.6, $\mathscr {H}$ is phylogenetic.

Finally, by definition, every clustering system $\mathfrak {C}$ on *X* is grounded and thus, $\mathscr {H}(\mathfrak {C})$ is regular. Since *X* is the unique inclusion-maximal cluster in $\mathfrak {C}$, it follows that *X* is the unique root of $\mathscr {H}(\mathfrak {C})$. Taking the latter arguments together, $\mathscr {H}(\mathfrak {C})$ is a regular network for clustering systems $\mathfrak {C}$. $\square$

Next, we show that, roughly speaking, DAGs with the strong-(CL) property differ from regular DAGs only by the presence of additional shortcuts.

### Theorem 4.8

A DAG *G* has the strong-(CL) property if and only if *G* is isomorphic to the regular DAG $\mathscr {H}(\mathfrak {C}_G)$ to which $\ell \ge 0$ shortcuts have been added.

### Proof

Suppose that *G* is a DAG on *X* with the strong-(CL) property. Let $H\doteq \mathscr {H}(\mathfrak {C}_G)$ in which also every inner vertex *C* obtains a new label *v* for some $v\in V(G)$ with ${{\,\mathrm{\texttt{C}}\,}}_G(v)=C$. By definition, *H* is a DAG on *X*. By Theorem 4.5, ${{\,\mathrm{\texttt{C}}\,}}_G(u)\ne {{\,\mathrm{\texttt{C}}\,}}_G(v)$ for all distinct $u,v\in V(G)$ and thus, $v\in V(G)$ if and only if there is a unique cluster $C\in \mathfrak {C}_G$ such that $C= {{\,\mathrm{\texttt{C}}\,}}_G(v)$. Hence, the aforementioned relabeling of the inner vertex is well-defined and uniquely determined and we have, in particular, $V(H) = V(G)$ and ${{\,\mathrm{\texttt{C}}\,}}_H(v) = {{\,\mathrm{\texttt{C}}\,}}_G(v)$ for all $v\in V(G)$. Note that, since *G* is a DAG, $\mathfrak {C}_G$ is a grounded set system and Lemma 4.7 implies that $\mathscr {H}{(\mathfrak {C}_G)}$ is regular. Since $\mathscr {H}{(\mathfrak {C}_G)}\simeq H$, the DAG *H* is regular. We show now that *H* is a subgraph of *G*. Let $u,v\in V$ be such that ${{\,\mathrm{\texttt{C}}\,}}_G(u)\subsetneq {{\,\mathrm{\texttt{C}}\,}}_G(v)$ and there is no cluster $C \in \mathfrak {C}_G$ such that ${{\,\mathrm{\texttt{C}}\,}}_G(u)\subsetneq C \subsetneq {{\,\mathrm{\texttt{C}}\,}}_G(v)$. Thus, $u\ne v$ and Theorem 4.5 implies that $u\prec _G v$. Hence, there is a directed *vu*-path *P* in *G*. If there would be vertex *w* in *P* such that $u\prec _G w \prec _G v$, then Theorem 4.5 together with ${{\,\mathrm{\texttt{C}}\,}}_G(w) \ne {{\,\mathrm{\texttt{C}}\,}}_G(v)$ and ${{\,\mathrm{\texttt{C}}\,}}_G(w) \ne {{\,\mathrm{\texttt{C}}\,}}_G(u)$ implies that ${{\,\mathrm{\texttt{C}}\,}}_G(u)\subsetneq {{\,\mathrm{\texttt{C}}\,}}_G(w) \subsetneq {{\,\mathrm{\texttt{C}}\,}}_G(v)$; a contradiction. Consequently, *P* just consists of the single edge $(u,v)\in E(G)$. By definition of regular DAGs these type of edges (*u*, *v*) are precisely the edges in *H* and, therefore, $E(H)\subseteq E(G)$, i.e., *H* is a subgraph of *G* with $V(H) = V(G)$.

Now, let $(u,v) = e\in E(G)\setminus E(H)$ and thus, $v\prec _G u$. Since ${{\,\mathrm{\texttt{C}}\,}}_G(u)\ne {{\,\mathrm{\texttt{C}}\,}}_G(v)$, Lemma 2.1 implies ${{\,\mathrm{\texttt{C}}\,}}_G(v)\subsetneq {{\,\mathrm{\texttt{C}}\,}}_G(u)$. As argued above, $u,v\in V(G) = V(H)$ and ${{\,\mathrm{\texttt{C}}\,}}_H(v) = {{\,\mathrm{\texttt{C}}\,}}_G(v)$ and ${{\,\mathrm{\texttt{C}}\,}}_H(u) = {{\,\mathrm{\texttt{C}}\,}}_G(u)$. Thus, ${{\,\mathrm{\texttt{C}}\,}}_H(v)\subsetneq {{\,\mathrm{\texttt{C}}\,}}_H(u)$. Since *H* is regular, Theorem 4.6 implies that *H* satisfies (PCC). Hence, $u\prec _H v$ or $v\prec _H u$ holds. However, $u\prec _H v$ would together with Lemma 2.1 imply that ${{\,\mathrm{\texttt{C}}\,}}_H(u)\subsetneq {{\,\mathrm{\texttt{C}}\,}}_H(v)$; a case that cannot occur. Thus, only $v\prec _H u$ is possible. Hence, there is a directed path from *u* to *v* in *H*. Since $E(H)\subseteq E(G)$, this path exists in *G* and, in particular, avoids the edge (*u*, *v*). Hence, (*u*, *v*) is a shortcut in *G*. As the latter arguments hold for all edges in $E(G)\setminus E(H)$, every edge in $E(G)\setminus E(H)$ is a shortcut in *G*. Thus, *G* is isomorphic to the regular DAG $H\doteq \mathscr {H}(\mathfrak {C}_G)$ to which $\ell \ge 0$ shortcuts have been added.

Let $H\doteq \mathscr {H}(\mathfrak {C}_G)$ and suppose now that *G* is isomorphic to $H'$, where $H'$ is is obtained from *H* by adding $\ell \ge 0$ shortcuts. Since there is a bijection between *V*(*G*) and $V(H')$, we can w.l.o.g. assume that $V:=V(G) = V(H') = V(H)$. This together with stepwise application of Lemma 2.5 implies that ${{\,\mathrm{\texttt{C}}\,}}_H(v)= {{\,\mathrm{\texttt{C}}\,}}_{G}(v)$ for all $v\in V$. By definition of *H*, ${{\,\mathrm{\texttt{C}}\,}}_H(v)\ne {{\,\mathrm{\texttt{C}}\,}}_{H}(u)$ and, therefore, ${{\,\mathrm{\texttt{C}}\,}}_G(v)\ne {{\,\mathrm{\texttt{C}}\,}}_{G}(u)$ for all distinct $u,v \in V$. In addition, Theorem 4.6 implies that *H* satisfies (PCC) implies that *G* satisfies (PCC). This allows us to apply Theorem 4.5 and to conclude that *G* has the strong-(CL) property. $\square$

By Observation 3.8 every $\mathscr {I}^{1}$-$\operatorname {lca}$-$\textsc {Rel}$ DAG is $\operatorname {lca}$-$\textsc {Rel}$ and, by Theorem 4.5, has the strong-(CL) property. This together with Theorem 4.8 implies

### Corollary 4.9

Every $\mathscr {I}^{1}$-$\operatorname {lca}$-$\textsc {Rel}$ DAG *G* from which all shortcuts have been removed is regular.

The converse of Corollary 4.9 is, in general, not satisfied without specifying $\mathscr {I}^{1}$, i.e., not every regular DAG is $\mathscr {I}^{1}$-$\operatorname {lca}$-$\textsc {Rel}$ for arbitrary $\mathscr {I}^{1}$. By way of example, the regular network $N_1$ in Fig. 3 is not $\{1,2\}$-$\operatorname {lca}$-Rel. Nevertheless, we obtain the following new characterization of regular DAGs.

### Theorem 4.10

For every DAG *G*, the following statements are equivalent.

1. *G* is regular.
2. *G* is shortcut-free and has the strong-(CL) property.
3. *G* is shortcut-free and $\operatorname {lca}$-Rel.

### Proof

The equivalence between (1) and (2) is an immediate consequence of Theorem 4.8 and 4.6. The equivalence between (2) and (3) follows from Theorem 4.5. $\square$

Theorem 4.10 together with Lemma 4.7 implies

### Corollary 4.11

For every grounded set system $\mathfrak {C}$, there is a phylogenetic $\operatorname {lca}$-Rel, and thus also $\operatorname {LCA}$-Rel, DAG *G* with $\mathfrak {C}_G = \mathfrak {C}$.

As argued in the example succeeding Def. 2.6, $\mathscr {H}(\mathfrak {C})$ is in general not regular in case $\mathfrak {C}$ is not grounded. Moreover, for every DAG *G*, the set system $\mathfrak {C}_G$ is always grounded. Consequently, the requirement that $\mathfrak {C}$ is grounded cannot be omitted in Corollary 4.11.

In phylogenetic trees, the number of inner vertices and edges is bounded from above by a linear function on the number of its leaves. In general, phylogenetic DAGs and thus, general DAGs, lack this property. For $\operatorname {lca}$-Rel DAGs and thus, also $\mathscr {I}^{1}$-$\operatorname {lca}$-Rel DAGs, we nevertheless obtain the following simple result.

### Lemma 4.12

The number of vertices and edges in $\operatorname {lca}$-Rel DAGs *G* is asymptotically bounded from above by a function depending only on the number of leaves and it holds that $|V(G)| = |\mathfrak {C}_G|$.

### Proof

Let $G=(V,E)$ be an $\operatorname {lca}$-Rel DAGs *G* on *X*. Hence, by the equivalence between Statements (2) and (3) of Theorem 4.5 it holds that $u\ne v$ implies ${{\,\mathrm{\texttt{C}}\,}}_G(u)\ne {{\,\mathrm{\texttt{C}}\,}}_G(v)$, for all $u,v\in V(G)$. Trivially, ${{\,\mathrm{\texttt{C}}\,}}_G(u)\ne {{\,\mathrm{\texttt{C}}\,}}_G(v)$ implies $u\ne v$. Taken the latter two arguments together, $|V(G)| = |\mathfrak {C}_G|$. Clearly $|\mathfrak {C}_G| \in O(2^{|X|})$ and, therefore, the number of vertices in *G* is asymptotically bounded from above by a function on the number of leaves. As the number of edges in any DAG *G* is always bounded from above by a function on the number of vertices in *G*, the number of edges in *G* is asymptotically bounded above by a function on the number of leaves. $\square$

Lemma 4.12 cannot be extended to the case of $\operatorname {LCA}$-Rel DAGs. To see this, consider, for example, the DAG $G_k$ obtained from any $\operatorname {LCA}$-$\textsc {Rel}$ network *N* on *X* by adding *k* additional roots $r_1$,..., $r_k$ connected to the leaves in *X* by edges $(r_i,x)$ for each $x\in X$ and $1\le i\le |X|$. In this case, $G_k$ remains $\operatorname {LCA}$-$\textsc {Rel}$ and $|V(G_k)|=|V(N)|+k$. Since *k* does not depend on |*X*| and can be chosen arbitrarily, no upper bound on $|V(G_k)|$ depending on |*X*| can be found.

## The $\mathbf {\ominus }$-Operator and Computation of lca- & LCA-Relevant DAGs

Not all DAGs are $\mathscr {I}^{1}$-$\operatorname {lca}$-Rel or $\mathscr {I}^{1}$-$\operatorname {LCA}$-Rel. This raises the question of whether it is possible to “transform” a non-$\mathscr {I}^{1}$-$\operatorname {lca}$-Rel resp., non-$\mathscr {I}^{1}$-$\operatorname {LCA}$-Rel DAG *G* into an $\mathscr {I}^{1}$-$\operatorname {lca}$-Rel, resp., $\mathscr {I}^{1}$-$\operatorname {LCA}$-Rel DAG *H* while preserving as many structural properties of *G* as possible. To clarify, for a given DAG *G* on *X*, we aim to maintain the following structural properties in *H*:

(*S*1): *H* remains a DAG on *X*.
(*S*2): $V(H) \subseteq V(G)$, meaning no new vertices are introduced.
(*S*3): *H* preserves the ancestor relationship $\prec _G$, i.e., $u \prec _G w$ if and only if $u \prec _{H} w$ for all $u, w \in V(H)$.
(*S*4): *H* is $\mathscr {I}^{1}$-$\operatorname {lca}_G$-*preserving*, i.e., $\operatorname {lca}_{H}(A) = \operatorname {lca}_G(A)$ for all $A\in X(\mathscr {I}^{1})$ for which $\operatorname {lca}_G(A)$ is well-defined.

In case we are interested in $\mathscr {I}^{1}$-$\operatorname {LCA}$-Rel DAG, we strengthen (S4) to

(*S*5): *H* is $\mathscr {I}^{1}$-$\operatorname {LCA}_G$-*preserving*, i.e., $\operatorname {LCA}_{H}(A) = \operatorname {LCA}_G(A)$ for all $A\in X(\mathscr {I}^{1})$.

Note that (S5) implies (S4). Moreover, Property (S4), resp., (S5) implies that that $\mathscr {I}^{1}$-$\operatorname {lca}$, resp., $\mathscr {I}^{1}$-$\operatorname {LCA}$ vertices in *G* remain $\mathscr {I}^{1}$-$\operatorname {lca}$, resp., $\mathscr {I}^{1}$-$\operatorname {LCA}$ vertices in *H*. This together with (S2) implies that no new vertices that violate the property of being $\mathscr {I}^{1}$-$\operatorname {lca}$, resp., $\mathscr {I}^{1}$-$\operatorname {LCA}$ vertices are introduced.

Moreover, Property (S1) states that $L(H)=L(G)=X$. In addition, for all $C\in \mathfrak {C}_H$, there is a vertex $v\in V(H)$ such that ${{\,\mathrm{\texttt{C}}\,}}_H(v)=C$. By (S2), it holds that $v\in V(G)$ and by (S3) we have ${{\,\mathrm{\texttt{C}}\,}}_H(v)={{\,\mathrm{\texttt{C}}\,}}_G(v)$. Consequently, each $C\in \mathfrak {C}_H$ is contained in $\mathfrak {C}_G$. We summarize the latter into

### Observation 5.1

If *H* satisfies (S1), (S2) and (S3) w.r.t. to a DAG *G*, then the following property is satisfied:

(*S*0): $\mathfrak {C}_{H} \subseteq \mathfrak {C}_G$, meaning no new clusters are introduced.

A powerful tool in this context is the following $\ominus$-operator.

### Definition 5.2

(Shanavas et al. 2024) Let $G=(V,E)$ be a DAG and $v\in V$. Then $G\ominus v=(V',E')$ is the directed graph with vertex set $V'=V\setminus \{v\}$ and edges $(p,q)\in E'$ precisely if $v\ne p$, $v\ne q$ and $(p,q)\in E$, or if $(p,v)\in E$ and $(v,q)\in E$. For a non-empty subset $W = \{w_1,\dots ,w_\ell \} \subsetneq V$, define $G\ominus W :=(\dots ((G \ominus w_1) \ominus w_2) \dots )\ominus w_\ell$.

In simple words, the directed graph $G\ominus v$ is obtained from $G=(V,E)$ by removing *v* and its incident edges and connecting each parent *p* of *v* with each child *q* of *v*. In case *v* is a leaf or a root in *G*, then *v* and its incident edges are simply deleted. The $\ominus$-operator was formally introduced in Shanavas et al. (2024) and is also known as collapse in the Biopython package (Cock et al. 2009; Talevich et al. 2012), or as “suppression” when the vertex in question has both in-degree and out-degree one (Huson and Scornavacca 2011). However, the properties of the $\ominus$-operator seem not to have been studied in the literature so far.

By construction, $G\ominus v$ remains a DAG such that $p\preceq _G q$ if and only if $p\preceq _{G\ominus v} q$ for all $p,q\ne v$. This preservation of the partial order implies that also the clusters remain unchanged as long as the deleted vertex is not a leaf. Moreover, if $v\in V\setminus X$, then the latter arguments imply that leaves of *G* remain leaves in $G\ominus v$, i.e., $G\ominus v$ is a DAG on *X*. Finally, it is an easy task to verify that for distinct $u,v\in V$ it holds that $(G\ominus u)\ominus v=(G\ominus v)\ominus u$. Thus, $G\ominus W$ is well-defined. We summarize the latter into

### Observation 5.3

Let *G* be a DAG on *X* and $W\subseteq V(G)\setminus X$ be a non-empty subset. Then, $G\ominus W$ is a DAG on *X* that satisfies (S1), (S2), (S3) and, by Observation 5.1, also (S0). In particular, ${{\,\mathrm{\texttt{C}}\,}}_G(u)={{\,\mathrm{\texttt{C}}\,}}_{G\ominus W}(u)$ for all $u\in V(G\ominus W)$.

We provide now a sufficient condition under which the $\ominus$-operator preserves connectivity.

### Lemma 5.4

Let $G=(V,E)$ be a connected DAG on *X* and $v\in V$. If *v* is not a ${{\,\mathrm{\textit{k}-LCA}\,}}$ or ${{\,\mathrm{\textit{k}-lca}\,}}$ vertex of *G* for any $k\in \{1,\ldots ,|X|\}$, then $G\ominus v$ is connected.

### Proof

Let $G=(V,E)$ be a connected DAG on *X* and $v\in V$ a vertex that is not a ${{\,\mathrm{\textit{k}-LCA}\,}}$ or ${{\,\mathrm{\textit{k}-lca}\,}}$ vertex of *G* for any $k\in \{1,\ldots ,|X|\}$. Assume, for contradiction, that $G\ominus v$ is not connected. Hence, there are distinct vertices *u*, *w* in $G\ominus v$ for which there is no *uw*-path in $G\ominus v$. Since *G* is connected and $u,w\in V(G\ominus v)\subseteq V(G)$, there is an *uw*-path $P_{uw}$ in *G* which, by assumption, is not contained in $G\ominus v$. Hence, $P_{uw}$ must contain the vertex *v* and *u*, *v*, *w* are pairwise distinct. To recall, paths do not contain “repeated” vertices, that is, *v* is contained in exactly two edges contained in $P_{uw}$. Let $u'$, resp., $w'$ be the neighbor of *v* along the subpath of $P_{uw}$ from *v* to *u*, resp., *v* to *w*. Note that $u'=u$ or $w'=w$ is possible. Hence $P_{uw}$ consists of an $uu'$-path $P_{uu'}$, an $u'w'$-path $P_{u'w'}$ that contains *v* and consist of exactly two edges and a $w'w$-path $P_{w'w}$.

Note that any $u'w'$-path $P'$ in $G\ominus v$ would imply the existence of an undirected *uw*-path in $G\ominus v$, by combining $P_{uu'}$, $P'$ and $P_{w'w}$. Since there is no *uw*-path in $G\ominus v$, it follows that there is no $u'w'$-path in $G\ominus v$. Note that in *G* the vertices $u'$ and $w'$ must be children or parents of *v*. Hence, we consider the following three possible cases that can appear in *G*: $\{w',u'\}$ contains (i) one parent and one child of *v*, (ii) two parents of *v* and (iii) two children of *v*.

In Case (i), we can assume without loss of generality that $w'$ is a child of *v* and $u'$ a parent of *v* in *G*. By construction, we have in $G\ominus v$ the edge $(u',w')$ and, therefore, an $u'w'$-path in $G\ominus v$; a contradiction.

In Case (ii), both $u'$ and $w'$ are parents of *v*. Since every leaf is a 1-$\operatorname {lca}$ vertex of *G* and *v* is, in particular, not a 1-$\operatorname {lca}$ vertex, *v* must have some child $v'$ in *G*. By construction, we have in $G\ominus v$ the edges $(u',v')$ and $(w',v')$ which results in an $u'w'$-path in $G\ominus v$; a contradiction.

In Case (iii), both $u'$ and $w'$ are children of *v*. Consider the set $A:={{\,\mathrm{\texttt{C}}\,}}_{G}(u')\cup {{\,\mathrm{\texttt{C}}\,}}_{G}(w')$. Since $u',w'\prec _G v$, Lemma 2.1 ensures that $A\subseteq {{\,\mathrm{\texttt{C}}\,}}_G(v)$ and hence, *v* is a common ancestor of the elements of *A*. Therefore, $\operatorname {LCA}_G(A)\ne \emptyset$. If *v* is not a ${{\,\mathrm{\textit{k}-LCA}\,}}$ vertex, then $v\notin \operatorname {LCA}_G(A)$. If *v* is not a ${{\,\mathrm{\textit{k}-lca}\,}}$ vertex, then $v\notin \operatorname {LCA}_G(A)$ or $v\in \operatorname {LCA}_G(A)$ and $|\operatorname {LCA}_G(A)|>1$. In either case, we may choose $z\in \operatorname {LCA}_G(A)$ such that $z\ne v$. Thus $z\in V(G\ominus v)$. By Observation 5.3 we have ${{\,\mathrm{\texttt{C}}\,}}_{G\ominus v}(u')={{\,\mathrm{\texttt{C}}\,}}_G(u')$, ${{\,\mathrm{\texttt{C}}\,}}_{G\ominus v}(w')={{\,\mathrm{\texttt{C}}\,}}_G(w')$ and ${{\,\mathrm{\texttt{C}}\,}}_{G\ominus v}(z)={{\,\mathrm{\texttt{C}}\,}}_G(z)$. Clearly there are paths in $G\ominus v$ from $u'$ to every element in ${{\,\mathrm{\texttt{C}}\,}}_{G\ominus v}(u')$ respectively from $w'$ to every element in ${{\,\mathrm{\texttt{C}}\,}}_{G\ominus v}(w')$. Moreover, there are paths from *z* to every element in ${{\,\mathrm{\texttt{C}}\,}}_{G\ominus v}(z)$. Since ${{\,\mathrm{\texttt{C}}\,}}_{G\ominus v}(z)={{\,\mathrm{\texttt{C}}\,}}_G(z)$ contains every element of *A*, there is a $u'w'$-path in $G\ominus v$; a contradiction.

In summary, all three possible Cases (i), (ii) and (iii) yield a contradiction. Consequently, $G\ominus v$ must be connected. $\square$

**Fig. 5:**
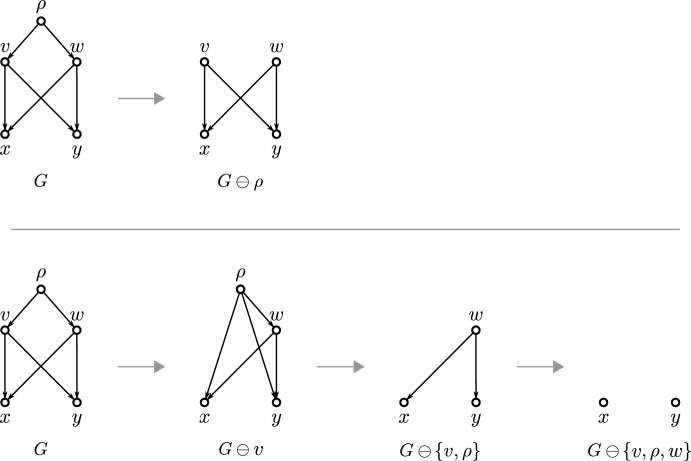
The network *G* is neither $\operatorname {lca}$-Rel nor $\operatorname {LCA}$-Rel, since none of the vertices *v*, *w* and $\rho$ in *G* are $\{1,2\}$-$\operatorname {lca}$ vertices. Since $\rho$ is the only vertex that is not a $\{1,2\}$-$\operatorname {LCA}$, $G\ominus \rho$ is $\operatorname {LCA}$-Rel. The set $W=\{\rho ,v,w\}$ is the set of all vertices that are not $\{1,2\}$-$\operatorname {lca}$ vertices. The stepwise computation of $G\ominus v$, $(G\ominus v)\ominus \rho$ and $G\ominus W$ is shown in the lower part and results in a disconnected DAG. However, Algorithm 3 determines whether a vertex is a $\{1,2\}$-$\operatorname {lca}$ vertex in the updated DAG. Hence, if we start with *v* to obtain $G\ominus v$, there is only one vertex left that is not an $\{1,2\}$-$\operatorname {lca}$ vertex, namely $\rho$. In $(G\ominus v)\ominus \rho$ each vertex is a $\{1,2\}$-$\operatorname {lca}$ vertex and the algorithm terminates

In contrast to Lemma 5.4, $G\ominus v$ may be disconnected if *v* is a ${{\,\mathrm{\textit{k}-lca}\,}}$ vertex or a ${{\,\mathrm{\textit{k}-LCA}\,}}$ vertex, even if *G* is connected The possibly simplest example here is the DAG *H* consisting of a single root *w* with leaf-children *x* and *y*; in *H*, the root satisfy $w=\operatorname {lca}_G(\{x,y\})$ and $G\ominus w$ is the disconnected DAG $(\{x,y\},\emptyset )$, see also Fig. 5.

### Theorem 5.5

Let *G* be a DAG on *X* and $W\subseteq V(G)$ be a non-empty subset of vertices that are not $\mathscr {I}^{1}$-$\operatorname {lca}$ (resp., not $\mathscr {I}^{1}$-$\operatorname {LCA}$) vertices in *G*. Then, $G\ominus W$ is a DAG on *X* that satisfies (S0)- (S4) (resp., (S0)–(S5)) w.r.t. *G*.

In particular, if *W* contains every vertex of *G* that is not an $\mathscr {I}^{1}$-$\operatorname {lca}$ vertex (resp. not an $\mathscr {I}^{1}$-$\operatorname {LCA}$ vertex) of *G*, then $G\ominus W$ is $\mathscr {I}^{1}$-$\operatorname {lca}$-$\textsc {Rel}$ (resp. $\mathscr {I}^{1}$-$\operatorname {LCA}$-$\textsc {Rel}$).

### Proof

Let $G=(V,E)$ be a DAG on *X* and $W\subseteq V$ be a non-empty subset of vertices that are not $\mathscr {I}^{1}$-$\operatorname {lca}$ (resp., not $\mathscr {I}^{1}$-$\operatorname {LCA}$) vertices. Observe first that for all $x\in X$ we have, by definition, $\operatorname {LCA}_G(x)=\{x\}$ and thus, $W\subseteq V{\setminus } X$. This together with Observation 5.3 implies that $G\ominus W$ is a DAG on *X* that satisfies (S0), (S1), (S2) and (S3) and ${{\,\mathrm{\texttt{C}}\,}}_G(w)={{\,\mathrm{\texttt{C}}\,}}_{G\ominus W}(w)$ for all $w\in V(G\ominus W)$.

We show first that $u\in \operatorname {LCA}_{G}(A)$ and $u\ne v$ implies that $u\in \operatorname {LCA}_{G\ominus v}(A)$ for any vertex $v\in V\setminus X$ and $A\subseteq X$. By (S2), $u\in V(G\ominus v)$. By Lemma 3.1, $u\in \operatorname {LCA}_G(A)$ only if $A\subseteq {{\,\mathrm{\texttt{C}}\,}}_G(u)$ and $A\not \subseteq {{\,\mathrm{\texttt{C}}\,}}_G(u')$ for all $u'\in V(G)$ with $u'\prec _G u$. Since ${{\,\mathrm{\texttt{C}}\,}}_G(u)={{\,\mathrm{\texttt{C}}\,}}_{G\ominus v}(u)$, we have $A\subseteq {{\,\mathrm{\texttt{C}}\,}}_{G\ominus v}(u)$. Since $G\ominus v$ satisfies (S3) and since ${{\,\mathrm{\texttt{C}}\,}}_G(w)={{\,\mathrm{\texttt{C}}\,}}_{G\ominus v}(w)$ for all $w\in V(G\ominus v)$, we can conclude that $A\not \subseteq {{\,\mathrm{\texttt{C}}\,}}_{G\ominus v}(u')$ for all $u'\in V(G\ominus v)$ with $u'\prec _{G\ominus v} u$. Application of Lemma 3.1 now shows that $u\in \operatorname {LCA}_{G\ominus v}(A)$ must hold.

Suppose now that *W* is a subset of non-$\mathscr {I}^{1}$-$\operatorname {LCA}$ vertices in *G* and let $v\in W$. We show now that $\operatorname {LCA}_{G\ominus v}(A) = \operatorname {LCA}_G(A)$ for all $A\in X(\mathscr {I}^{1})$. Let $A\in X(\mathscr {I}^{1})$ and assume first that $\operatorname {LCA}_{G}(A) = \emptyset$. This in particular implies that there is no vertex $w\in V$ such that $A\subseteq {{\,\mathrm{\texttt{C}}\,}}_G(w)$. Since ${{\,\mathrm{\texttt{C}}\,}}_G(w)={{\,\mathrm{\texttt{C}}\,}}_{G\ominus v}(w)$ for all $w\in V(G\ominus v)$ it follows that there is no vertex $w\in V(G\ominus v)$ such that $A\subseteq {{\,\mathrm{\texttt{C}}\,}}_{G\ominus v}(w)$. Hence, $\operatorname {LCA}_{G\ominus v}(A) = \emptyset$. Assume now that $\operatorname {LCA}_{G}(A)\ne \emptyset$. Hence, there is some vertex $u\in \operatorname {LCA}_{G}(A)$. Since *v* is not an $\mathscr {I}^{1}$-$\operatorname {LCA}$ vertex, $u\ne v$ and $v\notin \operatorname {LCA}_G(A)$ must hold. This together with the result of the preceding paragraph implies that $\operatorname {LCA}_G(A)\subseteq \operatorname {LCA}_{G\ominus v}(A)$. Conversely, assume that $u\in \operatorname {LCA}_{G\ominus v}(A)$. By Lemma 3.1, $A\subseteq {{\,\mathrm{\texttt{C}}\,}}_{G\ominus v}(u)$ and $A\not \subseteq {{\,\mathrm{\texttt{C}}\,}}_{G\ominus v}(u')$ for all $u'\in V(G\ominus v)$ with $u'\prec _{G\ominus v} u$ must hold. If $v\not \prec _{G} u$, then Observation 5.3 together with (S2) implies that $A\subseteq {{\,\mathrm{\texttt{C}}\,}}_{G }(u)$ and $A\not \subseteq {{\,\mathrm{\texttt{C}}\,}}_{G}(u')$ for all $u'\in V(G)$ with $u'\prec _{G} u$, in which case, $u\in \operatorname {LCA}_{G}(A)$. Assume that $v\prec _{G} u$. Then either $A\not \subseteq {{\,\mathrm{\texttt{C}}\,}}_{G}(v)$ or $A\subseteq {{\,\mathrm{\texttt{C}}\,}}_{G}(v)$. In the first case, Lemma 3.1 implies that $u\in \operatorname {LCA}_{G}(A)$. In the last case, Lemma 3.1 together with the assumption that *v* is not an $\mathscr {I}^{1}$-$\operatorname {LCA}$ vertex in *G* implies that there must be a child *w* of *v* such that $A\subseteq {{\,\mathrm{\texttt{C}}\,}}_{G}(w)$. But then, $w\prec _G u$ and $w\in V{\setminus } \{v\}$ must hold. Again, Observation 5.3 together with (S2) implies that $w\prec _{G\ominus v} u$ and $A\subseteq {{\,\mathrm{\texttt{C}}\,}}_{G\ominus v}(w)$ which together with Lemma 3.1 implies that $u\notin \operatorname {LCA}_{G\ominus v}(A)$; a contradiction. Hence, $u\in \operatorname {LCA}_{G}(A)$ must hold. In summary, $\operatorname {LCA}_{G\ominus v}(A) = \operatorname {LCA}_G(A)$ for all $A\in X(\mathscr {I}^{1})$. Thus, $G\ominus v$ satisfies (S5) and, thus, in particular (S4). We can now repeat the latter arguments on $G\ominus v$ and an element in $v'\in W{\setminus } \{v\}$ to conclude that $(G\ominus v)\ominus v'$ is a DAG on *X* that satisfies $\operatorname {LCA}_{(G\ominus v)\ominus v'}(A) = \operatorname {LCA}_{G\ominus v}(A)= \operatorname {LCA}_{G}(A)$ for all $A\in X(\mathscr {I}^{1})$ and thus, that $(G\ominus v)\ominus v'$ satisfies (S4) and (S5). By induction, $G\ominus W$ is a DAG on *X* that satisfies (S4) and (S5).

Assume now that $v\in W$ is not an $\mathscr {I}^{1}$-$\operatorname {lca}$ vertex in *G*. Let $A\in X(\mathscr {I}^{1})$ and suppose that $u = \operatorname {lca}_G(A)$ is well-defined. Hence, $u\ne v$ and, since $G\ominus v$ satisfies (S2), $u\in V(G\ominus v)$. Moreover, by the arguments in the second paragraph of this proof, $u\in \operatorname {LCA}_{G\ominus v}(A)$. Assume, for contradiction, that $u\ne \operatorname {lca}_{G\ominus v}(A)$ and, thus, $|\operatorname {LCA}_{G\ominus v}(A)|>1$. Thus, there is a vertex $w\in \operatorname {LCA}_{G\ominus v}(A)$ such that *u* and *w* are $\preceq _{G\ominus v}$-incomparable. By (S2), *w* is $\preceq _G$-incomparable to *u*. By Observation 5.3 and Lemma 3.1, $A\subseteq {{\,\mathrm{\texttt{C}}\,}}_{G\ominus v}(w)={{\,\mathrm{\texttt{C}}\,}}_G(w)$. Hence, *w* is ancestor of all vertices $x\in A$ in *G*. Therefore, there is a vertex $w'\preceq _G w$ such that $w'\in \operatorname {LCA}_G(A)$. Since by assumption $\operatorname {LCA}_G(A) = \{u\}$ it follows that $w'=u$ must hold. But then *w* and *u* are not $\preceq _G$-incomparable; a contradiction. Thus, $u = \operatorname {lca}_{G\ominus v}(A)$ must hold and $G\ominus v$ satisfies (S4). Again, by induction, $G\ominus W$ satisfies (S4).

For the last statement, note that if *W* contains every vertex of *G* that is not an $\mathscr {I}^{1}$-$\operatorname {lca}$ vertex (resp., not an $\mathscr {I}^{1}$-$\operatorname {LCA}$ vertex), then $v\in V(G\ominus W)$ if and only if *v* is an $\mathscr {I}^{1}$-$\operatorname {lca}$ vertex (resp., an $\mathscr {I}^{1}$-$\operatorname {LCA}$ vertex) of *G*. Hence, $G\ominus W$ contains precisely all $\mathscr {I}^{1}$-$\operatorname {lca}$ vertices (resp., $\mathscr {I}^{1}$-$\operatorname {LCA}$ vertices) of *G*. As $G\ominus W$ satisfies (S4) (resp., (S5)) it follows that every vertex in $G\ominus W$ is an $\mathscr {I}^{1}$-$\operatorname {lca}$ (resp., $\mathscr {I}^{1}$-$\operatorname {LCA}$) vertex. Thus, $G\ominus W$ is $\mathscr {I}^{1}$-$\operatorname {lca}$-Rel (resp., $\mathscr {I}^{1}$-$\operatorname {LCA}$-Rel). $\square$

As we shall see in Sect. 7, it is in NP-hard to determine as whether a given DAG is $\mathscr {I}^{1}$-$\operatorname {lca}$-Rel or $\mathscr {I}^{1}$-$\operatorname {LCA}$-Rel for general $\mathscr {I}^{1}$. However, for the special case that $\mathscr {I}^{1}= \{1,2,\dots ,|X|\}$, we deal with $\operatorname {lca}$-Rel or $\operatorname {LCA}$-Rel DAGs. In fact, simplifying *G* into an $\operatorname {lca}$-Rel or $\operatorname {LCA}$-Rel DAG using the $\ominus$-operator is tractable and we provide here polynomial-time algorithms to achieve these transformations.

We start with Algorithm 2 to compute an $\operatorname {LCA}$-Rel version *H* of an input DAG *G* that satisfies (S0)–(S5). To recall, *G* is not $\operatorname {LCA}$-Rel if there is a vertex *v* in *G* such that *v* is not a least common ancestor for any non-empty $A\subseteq X$. By Lemma 3.4, the latter is precisely the case if $v\notin \operatorname {LCA}_G({{\,\mathrm{\texttt{C}}\,}}_G(v))$ which is the only condition that needs to be checked in Algorithm 2 (Line 3).
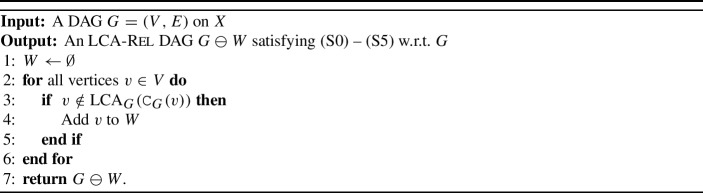

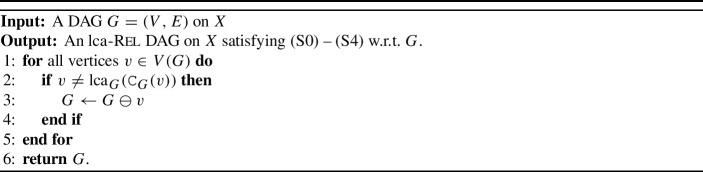


### Proposition 5.6

Let *G* be a DAG on *X* and *W* be the set of all vertices that are not $\{1,\ldots ,|X|\}$-$\operatorname {LCA}$ vertices of *G*. Then, Algorithm 2 with input *G* returns the DAG $G\ominus W$ on *X* that is $\operatorname {LCA}$-$\textsc {Rel}$, phylogenetic and satisfies Properties (S0)–(S5) w.r.t. *G*. In particular, *W* is the unique and, therefore, smallest subset of *V*(*G*) such that $G\ominus W$ is $\operatorname {LCA}$-$\textsc {Rel}$ and satisfies (S0)–(S5) w.r.t. *G*. Moreover, it holds that $\mathfrak {C}_{G\ominus W}=\mathfrak {C}_G$ and, if *G* is connected, then $G\ominus W$ is connected. Finally, Algorithm 2 can be implemented to run in polynomial time.

### Proof

Let $G=(V,E)$ be a DAG on *X* that serves as input for Algorithm 2. By construction in Line 3 and 4, the set *W* contains a vertex *w* if and only if $w\notin \operatorname {LCA}_G({{\,\mathrm{\texttt{C}}\,}}_G(w))$. By Lemma 3.4, the latter is precisely if *w* is not a $\{1,\ldots ,|X|\}$-$\operatorname {LCA}$ vertex of *G*. In other words, after the last iteration of the *for*-loop of Algorithm 2 the set *W* comprises all vertices of *G* that are not $\{1,\ldots ,|X|\}$-$\operatorname {LCA}$ vertices of *G*. By Theorem 5.5, the output DAG $H:=G\ominus W$ thus satisfies Properties (S0)–(S5) w.r.t. *G* and *H* is $\operatorname {LCA}$-$\textsc {Rel}$. Moreover, *H* is phylogenetic due to Lemma 3.10.

We continue with showing that *W* is the unique subset of *V* such that $H = G\ominus W$ is $\operatorname {LCA}$-$\textsc {Rel}$ and satisfies (S0)–(S5). Let $W^*$ be some subset of *V* such that $H^* :=G\ominus W^*$ is $\operatorname {LCA}$-$\textsc {Rel}$ and satisfies (S0)–(S5). Observe first that, since *H* and $H^*$ satisfy (S5), $\operatorname {LCA}_G(A) = \operatorname {LCA}_H(A) = \operatorname {LCA}_{H^*}(A)$ for all $A\subseteq X$. This together with *H* and $H^*$ being $\operatorname {LCA}$-$\textsc {Rel}$ implies that each vertex of *H* and $H^*$ is contained in $\operatorname {LCA}_G(A)$ for some $A\subseteq X$ and, in particular, $V(H^*) = \cup _{A\subseteq X} \operatorname {LCA}_G(A) = V(H)$. Consequently, $W^* = V\setminus V(H^*) = V\setminus V(H) = W$.

We show now that $\mathfrak {C}_G =\mathfrak {C}_H$. Since *H* satisfies Property (S0) w.r.t. *G*, it holds that $\mathfrak {C}_H\subseteq \mathfrak {C}_G$. To show that $\mathfrak {C}_G\subseteq \mathfrak {C}_H$, let $C\in \mathfrak {C}_G$ be a cluster of *G* and *v* a vertex of *G* such that ${{\,\mathrm{\texttt{C}}\,}}_G(v)=C$. Since *v* is a common ancestor of the vertices in *C*, there is some $u\in \operatorname {LCA}_G(C)$ such that $u\preceq _G v$. Note that $u\notin W$. Since $u\in \operatorname {LCA}_G(C)$, we have $C\subseteq {{\,\mathrm{\texttt{C}}\,}}_G(u)$. By Lemma 2.1, ${{\,\mathrm{\texttt{C}}\,}}_G(u)\subseteq {{\,\mathrm{\texttt{C}}\,}}_G(v)=C$. Taken the latter two arguments together, $C={{\,\mathrm{\texttt{C}}\,}}_G(u)$ must hold. Since $u\notin W$ and $V(H)=V(G\ominus W)$, *u* is a vertex of *H*. This together with Observation 5.3 implies that $C = {{\,\mathrm{\texttt{C}}\,}}_G(u) = {{\,\mathrm{\texttt{C}}\,}}_H(u)\in \mathfrak {C}_H$. In summary, $\mathfrak {C}_G =\mathfrak {C}_H$.

Assume now that *G* is connected. If $W =\emptyset$, then $G = G\ominus W$ and there is nothing to show. Hence suppose that $v\in W$. If $W = \{v\}$, then Lemma 5.4 implies that *H* is connected. Suppose that there is some $u\in W{\setminus } \{v\}$. We show that *u* cannot be a ${{\,\mathrm{\textit{k}-LCA}\,}}$ vertex in $G\ominus v$. To see this, observe first that, since $G\ominus v$ satisfies (S5), ${{\,\mathrm{\textit{k}-LCA}\,}}$ vertices of *G* remain ${{\,\mathrm{\textit{k}-LCA}\,}}$ vertices in $G\ominus v$. Hence, the set *U* of all vertices that are not $\{1,\ldots ,|X|\}$-$\operatorname {LCA}$ vertices of $G\ominus v$ is a subset of $W\setminus \{v\}$. Assume, for contradiction, that *u* is a ${{\,\mathrm{\textit{k}-LCA}\,}}$ vertex in $G\ominus v$ and thus, $U\subsetneq W{\setminus } \{v\}$. By Theorem 5.5, $(G\ominus v)\ominus U=G\ominus (U\cup \{v\})$ is $\operatorname {LCA}$-Rel. However, $U\subsetneq W\setminus \{v\}$ implies $U\cup \{v\}\subsetneq W$; the latter two statements yield a contradiction to the uniqueness of *W*. Thus, *u* is not a ${{\,\mathrm{\textit{k}-LCA}\,}}$ in $G\ominus v$. Now we can apply Lemma 5.4 to conclude that $(G\ominus v)\ominus u$ is connected. Repeating the latter arguments until all vertices in *W* have been processed shows that $G\ominus W$ is connected.

Finally, consider the runtime of Algorithm 2. The *if*-condition of the algorithm can be implemented to run in polynomial time, since the cluster ${{\,\mathrm{\texttt{C}}\,}}_G(v)$ can be computed by a simple post-order traversal of *G* and due to Corollary 3.12. Note furthermore that with an adjacency list representation of *G*, computation of $G\ominus v$ can be implemented in polynomial time for a given vertex *v*, as it amounts to adding at most $|{{\,\textrm{child}\,}}_G(v)|$ entries to each list associated to the respective parent of *v* in *G* (and there are at most $|V(G)|-1$ parents of *v*). In extension, $G\ominus W$ can be computed in polynomial time. Since the remaining tasks are clearly possible to perform in constant time, we conclude the overall runtime to be polynomial. $\square$

Although the set *W* of all non-$\{1,\ldots ,|X|\}$-$\operatorname {LCA}$ vertices of *G* (as chosen in Algorithm 2) is the unique minimum-sized set such that $G\ominus W$ is $\operatorname {LCA}$-Rel *and* satisfies (S0)–(S5), it is not necessarily the smallest set transforming *G* to an $\operatorname {LCA}$-Rel DAG, see Fig. 7 for an example.

**Fig. 6:**
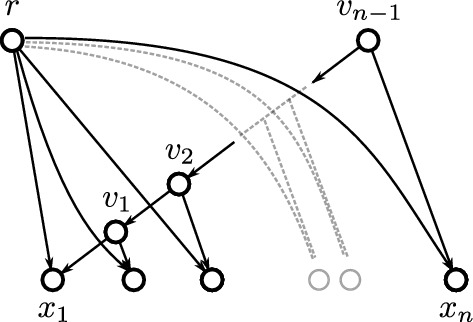
Consider the clustering system $\mathfrak {C}=\{\{x_1,x_2\},\{x_1,x_2,x_3\},\ldots ,X\} \cup \{\{x\} \mid x\in X\}$ on $X=\{x_1,\ldots ,x_n\}$, $n>1$. The DAG *G* on *X* as shown in the figure is obtained from $H\doteq \mathscr {H}(\mathfrak {C})$ by adding a second root *r* that is adjacent to each leaf $x_i\in X$. Here, for every non-empty $A\subseteq X$, we have $\operatorname {LCA}_G(A)=\{r,v_{i-1}\}$ where *i* is the maximal index such that $x_i\in A$. Consequently, *G* is $\operatorname {LCA}$-Rel but not $\operatorname {lca}$-$\textsc {Rel}$. The set *W* of all non-$\operatorname {lca}$ vertices of *G* is the set $V(G)\setminus X$ of all inner vertices of *G*. Here, $G\ominus W$ would be the disconnected DAG with vertex set *X* and no edges. The output of Algorithm 3 applied on *G* is always a connected DAG but heavily depends on the order in which the vertices have been considered. If *r* is processed first, i.e., before any of the $v_i$, then the output will be the $\operatorname {lca}$-Rel DAG $G\ominus r\simeq H$. In contrast, if *r* is processed last, i.e., after each $v_i$, then the $\operatorname {lca}$-Rel DAG $G\ominus \{v_1,\ldots ,v_{n-1}\}$ is returned. Here, $\mathfrak {C}_{G\ominus r}=\mathfrak {C}_G=\mathfrak {C}$ while $\mathfrak {C}_{G\ominus \{v_1,\ldots ,v_{n-1}\}}=\{X,\{x_1\},\ldots ,\{x_n\}\} \subsetneq \mathfrak {C}$

We next show that one can simplify a given DAG *G* to an $\operatorname {lca}$-Rel DAG *H* satisfying (S0)–(S4) in polynomial time. Recall that *H* is $\operatorname {lca}$-Rel if every vertex *v* in *H* is the unique least common ancestor for at least some set $A \subseteq X$. Unsurprisingly, a similar approach to that used in Algorithm 2 can be applied in the context of $\operatorname {lca}$-Rel DAGs as well. The reader may verify that by modifying the *if*-condition in Algorithm 2 to “*check if *$v \ne \operatorname {lca}_G({{\,\mathrm{\texttt{C}}\,}}_G(v))$”, one obtains an algorithm that, due to Lemma 3.5 and Theorem 5.5, outputs an $\operatorname {lca}$-Rel DAG that satisfies Properties (S1)–(S4). However, the output of this algorithm may be a disconnected DAG even if the initial input was connected, see Fig. 5 for an example. In particular, the set *W* of all non-$\operatorname {lca}$ vertices in *G* is not necessarily of minimum-size, that is, there are cases where $G\ominus W'$ is $\operatorname {lca}$-Rel for $W'\subsetneq W$, see Fig. 6 for an example. Informally, the approach in Algorithm 2 can be overly destructive: it removes *all* non-$\{1,\ldots ,|X|\}$-$\operatorname {lca}$ vertices, including those that are $\{1,\ldots ,|X|\}$-$\operatorname {LCA}$ vertices. To address this issue, we propose Algorithm 3 that, instead of taking *all* non-$\{1,\ldots ,|X|\}$-$\operatorname {lca}$, repeats the process of removing vertices only until we end up with an $\operatorname {lca}$-Rel DAG.

### Proposition 5.7

For a given input DAG *G* on *X*, Algorithm 3 returns a DAG *H* on *X* that is $\operatorname {lca}$-$\textsc {Rel}$, phylogenetic and satisfies Properties (S0)–(S4) w.r.t. *G*. Moreover, if *G* is connected, then *H* is connected. If *G* satisfies (PCC) or (CL), then $\mathfrak {C}_{H}=\mathfrak {C}_G$. In addition, Algorithm 3 can be implemented to run in polynomial time.

### Proof

To keep track of the original DAG in this proof, we put $G_{\textrm{orig}}:=G$ for the DAG $G=(V,E)$ on *X* that serves as input for Algorithm 3. We show, by induction on the number of calls of Line 3, that each updated DAG *G* satisfies (S0)–(S4) w.r.t. $G_{\textrm{orig}}$. As base case, if no calls appear, *G* trivially satisfies (S0)–(S4) w.r.t. $G_{\textrm{orig}}$. Suppose the statement is true prior to the current call of Line 3. Since Line 3 is called, $v\ne \operatorname {lca}_G({{\,\mathrm{\texttt{C}}\,}}_G(v))$ and Lemma 3.5 implies that *v* is not an $\mathscr {I}^{1}$-$\operatorname {lca}$ vertex in *G* for any $\mathscr {I}^{1}$. By Theorem 5.5, $G\ominus v$ satisfies (S0)–(S4) w.r.t. $G_{\textrm{orig}}$. The algorithm terminates after all vertices in *V* have been processed. Let *H* denote the final DAG that is returned by Algorithm 3. By the latter arguments and induction, *H* satisfies (S0)–(S4) w.r.t. $G_{\textrm{orig}}$.

We show now that *H* is $\mathscr {I}^{1}$-$\operatorname {lca}$-$\textsc {Rel}$ for $\mathscr {I}^{1}=\{1,2,\ldots ,|X|\}$ and hence, that *H* is $\operatorname {lca}$-$\textsc {Rel}$. Let *v* be the last vertex in the *for*-loop for which Line 3 is called and let *W* be the subset of all vertices in $V\setminus \{v\}$ for which Line 3 was called. By definition, $H = (G_{\textrm{orig}}\ominus W)\ominus v$. Assume, for contradiction, that *H* contains a vertex *u* that is not an $\mathscr {I}^{1}$-$\operatorname {lca}$ vertex. By Theorem 5.5, *H* satisfies (S4) w.r.t. $G_{\textrm{orig}}\ominus W$ and thus, if *w* is an $\mathscr {I}^{1}$-$\operatorname {lca}$ vertex in $G_{\textrm{orig}}\ominus W$ so it is in *H*. Contraposition of the latter statement implies that *u* is not an $\mathscr {I}^{1}$-$\operatorname {lca}$ vertex in $G_{\textrm{orig}}\ominus W$. Even more, *u* is not an $\mathscr {I}^{1}$-$\operatorname {lca}$ vertex in $G_{\textrm{orig}}\ominus W'$ for any subset $W'\subseteq W$. Hence, if *u* comes before *v* in the *for*-loop, it would have resulted in a call of Line 3 and so, $u\in W$; a contradiction. Therefore, *u* must come after *v* in the *for*-loop, i.e., *v* is not the last vertex for which Line 3 is called; also a contradiction. Therefore, all vertices in $G_{\textrm{orig}}\ominus (W\cup \{v\}) =H$ are $\{1,2,\ldots ,|X|\}$-$\operatorname {lca}$ vertices of *H* and, thus, *H* is $\operatorname {lca}$-Rel.

By Lemma 3.10, *H* is phylogenetic. Furthermore, if $G_{\textrm{orig}}$ is connected, then induction on the number of calls of Line 3 together with Lemma 5.4 implies that the output DAG *H* is connected.

Suppose now that $G_{\textrm{orig}}$ is a DAG that satisfies (CL). Since *H* satisfies (S0) w.r.t $G_{\textrm{orig}}$, we have $\mathfrak {C}_H\subseteq \mathfrak {C}_{G_{\textrm{orig}}}$. To see that $\mathfrak {C}_{G_{\textrm{orig}}}\subseteq \mathfrak {C}_H$, let $C\in \mathfrak {C}_{G_{\textrm{orig}}}$. Since $G_{\textrm{orig}}$ satisfies (CL), $\operatorname {lca}_{G_{\textrm{orig}}}(C)$ is well-defined, i.e. $u=\operatorname {lca}_{G_{\textrm{orig}}}(C)$ for some $u\in V(G_{\textrm{orig}})$. In particular, *u* is a |*C*|-$\operatorname {lca}$ vertex of $G_{\textrm{orig}}$. Since *H* satisfy (S4) w.r.t. $G_{\textrm{orig}}$, *u* is thus also a vertex of *H*. By Observation 5.3, ${{\,\mathrm{\texttt{C}}\,}}_H(u)={{\,\mathrm{\texttt{C}}\,}}_{G_{\textrm{orig}}}(u)$ and by Lemma 4.2 we have ${{\,\mathrm{\texttt{C}}\,}}_{G_{\textrm{orig}}}(u)={{\,\mathrm{\texttt{C}}\,}}_{G_{\textrm{orig}}}(\operatorname {lca}_{G_{\textrm{orig}}}(C))=C$. Thus ${{\,\mathrm{\texttt{C}}\,}}_H(u)=C$ and $C\in \mathfrak {C}_H$. In conclusion, $\mathfrak {C}_{G_{\textrm{orig}}}=\mathfrak {C}_H$ must hold. By Lemma 4.2, (PCC) implies (CL). This together with the latter arguments implies that $\mathfrak {C}_{G_{\textrm{orig}}}=\mathfrak {C}_H$ in case that $G_{\textrm{orig}}$ is a DAG that satisfies (PCC).

For the runtime of Algorithm 3, note that with an adjacency list representation of *G*, computation of $G\ominus v$ can be implemented in polynomial time for a given vertex *v*, as it amounts to adding at most $|{{\,\textrm{child}\,}}_G(v)|$ entries to each list associated to the respective parent of *v* in *G* (and there are at most $|V(G)|-1$ parents of *v*). Moreover, the *if*-condition of the algorithm can be implemented to run in polynomial time, since the cluster ${{\,\mathrm{\texttt{C}}\,}}_G(v)$ can be computed by a simple post-order traversal of *G* and due to Corollary 3.12. Hence every step of the *for*-loop of Algorithm 3 takes polynomial time, concluding the overall runtime to be polynomial. $\square$

While the set *W* of vertices used to transform a DAG *G* to an $\operatorname {lca}$-Rel DAG $G\ominus W$ is, in general, not uniquely determined (cf. Fig. 5 and 6), this situation changes whenever *G* satisfies (CL) or (PCC).

### Theorem 5.8

Let *G* be a DAG that satisfies (PCC) or (CL) and $W\subseteq V(G)$ be the set of all vertices that are not $\{1,\dots ,|X|\}$-$\operatorname {lca}$ vertices of *G*. Then, *W* is precisely the set of all vertices that are not $\{1,\dots ,|X|\}$-$\operatorname {LCA}$ vertices of *G*. Moreover, *W* is the unique and, therefore, smallest subset of *V*(*G*) such that $H:=G\ominus W$ is $\operatorname {lca}$-$\textsc {Rel}$ and satisfies (S0)–(S4) w.r.t. *G*.

Furthermore, it holds that $\mathfrak {C}_G=\mathfrak {C}_{H}$ and $H^-\simeq \mathscr {H}(\mathfrak {C}_G)$. In particular, *H* coincides with the DAG returned by Algorithm 3 with input *G*.

### Proof

Let *G* be a DAG on *X* that satisfies (PCC) or (CL) and $W\subseteq V(G)$ be the set of all vertices that are not $\{1,\dots ,|X|\}$-$\operatorname {lca}$ vertices of *G*. By Theorem 5.5, $H:=G\ominus W$ is $\operatorname {lca}$-$\textsc {Rel}$ and satisfies (S0)–(S4). By Theorem 4.5, *H* satisfies strong-(CL) and, thus, (CL). Hence, we can apply the same arguments as used in the proof of Proposition 5.7 to show that “$\mathfrak {C}_{G_{\textrm{orig}}}=\mathfrak {C}_H$” to conclude that $\mathfrak {C}_G=\mathfrak {C}_{H}$ holds. Since *H* satisfies strong-(CL) and $\mathfrak {C}_G=\mathfrak {C}_{H}$, we can apply Theorem 4.8 which implies that $H^-\simeq \mathscr {H}(\mathfrak {C}_G)$.

Now, let $W'$ be the set of all vertices that are not $\{1,\dots ,|X|\}$-$\operatorname {LCA}$ vertices of *G*. We show that $W=W'$. By definition, $W'\subseteq W$. Assume, for contradiction, that there is a vertex $w\in W\setminus W'$. Hence, *w* is a *k*-$\operatorname {LCA}$ vertex for some *k* and Lemma 3.4 together with Lemma 2.1 implies ${{\,\mathrm{\texttt{C}}\,}}_G(v)\subsetneq {{\,\mathrm{\texttt{C}}\,}}_G(w)$ for all children *v* of *w*. By Lemma 3.1, $w\in \operatorname {LCA}_G({{\,\mathrm{\texttt{C}}\,}}_G(w))$. By assumption, *G* satisfies (CL) or (PCC), where in the latter case, Lemma 4.2 implies that *G* satisfies (CL). Thus, $|\operatorname {LCA}_G({{\,\mathrm{\texttt{C}}\,}}_G(w))|=1$ and, therefore, $w = \operatorname {lca}_G({{\,\mathrm{\texttt{C}}\,}}_G(w))$; a contradiction to $w\in W$. Consequently, $W'= W$ holds.

We continue with showing that *W* is the uniquely determined set such that *H* is $\operatorname {lca}$-$\textsc {Rel}$ and satisfies (S0) – (S4). To this end, assume that there is some set $W''\subseteq V(G)$ such that $G\ominus W''$ is $\operatorname {lca}$-Rel and satisfies (S0) – (S4). Since $G\ominus W''$ satisfies (S4) w.r.t. *G*, the set $W''$ cannot contain any vertex that is *k*-$\operatorname {lca}$ vertex in *G* for some *k*, that is, $W''\subseteq W$. Assume, for contradiction, that $W''\subsetneq W$. Since $W''\subsetneq W=W'$, the set $W''$ is also a proper subset of vertices that are not $\{1,\dots ,|X|\}$-$\operatorname {LCA}$ vertices of *G*. By Theorem 5.5, $G\ominus W''$ satisfies (S0)–(S5). Moreover, since $G\ominus W''$ is $\operatorname {lca}$-Rel, it is, in particular, $\operatorname {LCA}$-Rel. However, this contradicts Proposition 5.6 which states that $W=W'$ is the unique and minimum-sized set such that $G\ominus W'$ is $\operatorname {LCA}$-Rel and satisfies (S0)–(S5), enforcing $W''=W$. Hence, *W* is the unique and, therefore, smallest subset of *V*(*G*) such that *H* is $\operatorname {lca}$-$\textsc {Rel}$ and satisfies (S0)–(S4). It is now straightforward to verify that *H* coincides with the DAG returned by Algorithm 3 with input *G*. $\square$

**Fig. 7:**
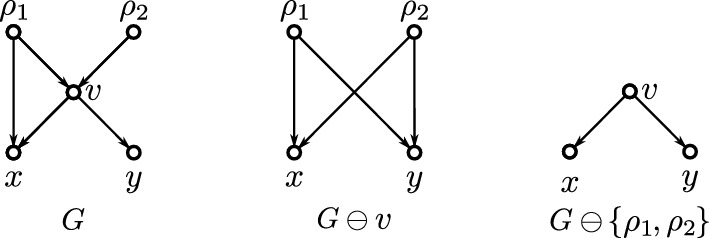
Shown are DAGs *G*, $G\ominus \{v\}$ and $G\ominus \{\rho _1,\rho _2\}$. The vertices $\rho _1$ and $\rho _2$ are not LCAs of any subset of leaves in *G*, while $v = \operatorname {lca}_G(\{x,y\})$. According to Proposition 5.6, $W = \{\rho _1,\rho _2\}$ is the unique and smallest set of vertices such that $G\ominus W$ is $\operatorname {LCA}$-Rel and satisfies (S0)–(S5). Nevertheless, the set $W'=\{v\}$ is the smallest set of vertices such that $G\ominus W'$ is $\operatorname {LCA}$-Rel. However, $G\ominus W'$ violates (S5) since $\operatorname {LCA}_G(\{x,y\})=\{v\} \ne \operatorname {LCA}_{G\ominus W'}(\{x,y\})=\{\rho _1,\rho _2\}$

Although the set *W* of all non-$\{1,\ldots ,|X|\}$-$\operatorname {lca}$ vertices of DAGs *G* with (PCC) or (CL) property is the unique and minimum-sized set such that $G\ominus W$ is $\operatorname {lca}$-Rel *and* satisfies (S0)–(S4), it is not necessarily a unique set transforming *G* to an $\operatorname {lca}$-Rel DAG. By way of example, consider the DAG *G* in Fig. 7. Here, $G\ominus \{r_1,v\} \simeq G\ominus \{r_1,r_2\}$ is $\operatorname {lca}$-Rel, but since $v = \operatorname {lca}_G(\{x,y\})\ne \operatorname {lca}_{G\ominus \{r_1,v\}}(\{x,y\})$, the DAG $G\ominus \{r_1,v\}$ does not satisfy (S4).

By Proposition 5.6, Algorithm 2 always outputs a DAG *H* with the same set system as the input DAG *G*, i.e., $\mathfrak {C}_G=\mathfrak {C}_{H}$. By Theorem 5.8, this property is also guaranteed whenever *G* satisfies (PCC) or (CL) when using Algorithm 3. In general, however, Algorithm 3 may return a DAG *H* with $\mathfrak {C}_H\subsetneq \mathfrak {C}_{G}$ depending on the order in which the vertices are traversed; see Fig. 6 for an illustrative example.

## The $\ominus$-Operator as Transformation to Simplify Networks

In a recent work, Heiss et al. (2025) proposed a general framework that every transformation $\varphi (N)$ that “simplifies” a network *N* should satisfy, stated as three axioms. To be more precise, let $\mathbb {N}(X)$ be the set of all networks on *X* and $\mathbb {N}'(X)\subseteq \mathbb {N}(X)$ be some subset of networks that is closed under permuting the leaves, i.e., if $N\in \mathbb {N}'(X)$ then $N^\sigma \in \mathbb {N}'(X)$, where $N^\sigma$ is the network obtained from *N* by relabeling the leaves in *X* according to some permutation $\sigma \in \Sigma ^X$ in the group $\Sigma ^X$ of permutations on *X*. Let *N*|*Y* be a restriction of *N* to a subset of leaves $Y\subseteq X$ that can be defined in different ways (Pardi and Scornavacca 2015; Heiss et al. 2025; Francis et al. 2021) (we will come to this point later again). A transformation is then a map

$$\begin{aligned} \varphi :\mathbb {N}(X) \rightarrow \mathbb {N}'(X)\subseteq \mathbb {N}(X) \end{aligned}$$

that assigns to each $N\in \mathbb {N}(X)$ a network $\varphi (N)\in \mathbb {N}'(X)$. Following Dress et al. (2010), Heiss et al. (2025) proposed three axioms that are desirable for such transformations, namely

1. $$N\in \mathbb {N}'(X)$$ $$\implies $$ $$\varphi (N)=N$$, and
2. $$\sigma \in \Sigma ^X$$, $$N\in \mathbb {N}(X)$$ $$\implies $$ $$\varphi (N^\sigma )\simeq \varphi (N)^\sigma $$, and
3. $$\emptyset \ne Y\subseteq X$$, $$N\in \mathbb {N}(X)$$ $$\implies $$ $$\varphi (N|Y)\simeq \varphi (N)|Y$$.

Property (P1) ensures that any transformation applied on $N\in \mathbb {N}'(X)$ always yields *N* unchanged. This is justified by the fact that one usually wants to transform or simplify a network to some network with specific properties encoded by the subclass $\mathbb {N}'(X)$. If our network *N* is contained in $\mathbb {N}'(X)$, then it has the required properties and thus, no further transformation is required. Property (P2) ensures that transformations are invariant under permutation of leaf labels: transforming a network with permuted leaf labels results in the same network as when one transforms the original network first and then relabel the leaves. In other words, the transformation is not dependent on the leaf labels. Finally, Property (P3) ensures that transformations are invariant under restrictions: taking a restricted network *N*|*Y* on a subset of leaves *Y* and transforming it results in the same network as that obtained by applying the transformation $\varphi (N)$ first on *N* and then taking the restriction $\varphi (N)|Y$. The latter two properties are mathematically sound but are also motivated from a biological point of view, see Dress et al. (2010), Heiss et al. (2025) for further details. What we have not yet defined is the concept of the restriction *N*|*Y*. Due to the lack of an axiomatic framework for “restriction”, several approaches to defining *N*|*Y* are possible. In Heiss et al. (2025), the authors defined one such restriction in terms of subnetworks induced by so-called $\textrm{LSA}$ vertices of *N* and their descendants. Using this definition, they demonstrated that the transformation $\varphi _{\textrm{LSA}}$ of phylogenetic networks to a specific tree, called the ${\textrm{LSA}}$-tree, satisfies properties (P1), (P2) and (P3). In particular, this type of restriction enforces (P3), ensuring that if additional species are added to a phylogenetic network (without otherwise altering the original network), transforming the enlarged network into an ${\textrm{LSA}}$-tree induces the same ${\textrm{LSA}}$-tree on the original species set as transforming the original network. However, there are examples that show that such ${\textrm{LSA}}$-trees lack our desired property (S3). To see this observe first that ${\textrm{LSA}}$-trees do, in general, not satisfy (S0), that is, the ${\textrm{LSA}}$-tree may contain clusters that are not contained in the original network *N*. As an example, the ${\textrm{LSA}}$-tree $\varphi _{\textrm{LSA}}(N)$ for the network *N*, shown in Fig. 9, includes a cluster containing *Rubellium*, *Chilenium* and *Erpetion* but not *Tridens*. This suggests that the first three taxa are more closely related evolutionarily compared to *Tridens*. However, such a cluster does not appear in $\mathfrak {C}_N$; instead, $\mathfrak {C}_N$ includes the cluster $\{\textit{Chilenium}, \textit{Erpetion}, \textit{Tridens}\}$. As a consequence of Observation 5.1 and since ${\textrm{LSA}}$-trees satisfy (S1) and (S2), ${\textrm{LSA}}$-trees violate, in general, Condition (S3).

The ${\textrm{LSA}}$ of a subset of leaves has different properties than the $\operatorname {LCA}$ or $\operatorname {lca}$ as defined here. For example the ${\textrm{LSA}}$ of a leaf $x\in X$ with in-degree one is its parent (Huson et al. 2011), whereas $\operatorname {lca}_G(x) = x$ and $\operatorname {LCA}_G(x) = \{x\}$. This makes their type of restriction not applicable to our developed methods. In particular, we want to show that the transformation of a network *N* with set *W* of non-$\operatorname {LCA}$ vertices into the network $N\ominus W$ from which all shortcuts have been removed has all three desired properties. However, it can be shown that this transformation does not satisfy (P3) when using a restriction defined by $\textrm{LSA}$s.

**Fig. 8:**
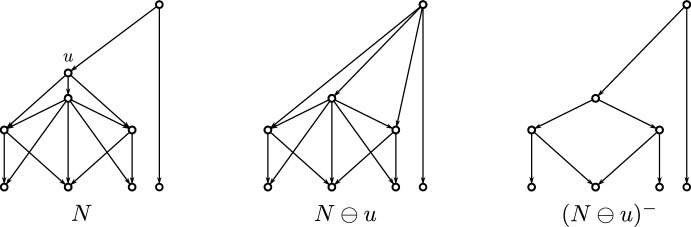
Shown are three phylogenetic networks *N*, $N\ominus u$ and $(N\ominus u)^-$ having the same clustering system $\mathfrak {C}_{N}=\mathfrak {C}_{N\ominus u} = \mathfrak {C}_{(N\ominus u)^-}=:\mathfrak {C}$. Here $(N\ominus u)^-\simeq N_1$ with $N_1$ being the network as shown in Fig. 2. In *N*, the vertex *u* is neither a *k*-$\operatorname {lca}$ nor a *k*-$\operatorname {LCA}$ vertex, for any *k*. In particular, *u* is the only vertex in *N* with this property. According to Proposition 5.6, $N\ominus u$ is $\operatorname {LCA}$-Rel and satisfies (S0)–(S5). In addition, Proposition 5.7 implies that $N\ominus u$ is $\operatorname {lca}$-Rel. Removal of all shortcuts in $N\ominus u$ yields $(N\ominus u)^-$ which is, by Corollary 4.11, regular and thus, isomorphic to the Hasse diagram $\mathscr {H}(\mathfrak {C})$. Still, $(N\ominus u)^-$ is $\operatorname {lca}$-Rel and satisfies (S0)–(S4). Note that, in this example, $\varphi _{\operatorname {LCA}}(N) = \varphi _{\operatorname {lca}}(N) = (N\ominus u)^- \simeq \mathscr {H}(\mathfrak {C}_{N})$, all satisfying (P1), (P2) and (P3)

Hence, we will consider a different type of restriction that is solely defined in terms of clusters of the DAGs under investigation. To be more precise, we define for a given DAG *G* on *X* and a subset $Y\subseteq X$ the *cluster-restriction*

$$\begin{aligned} G \wr Y:=\mathscr {H}(\mathfrak {C}_G\cap Y), \text { where } \mathfrak {C}_G\cap Y:=\{C\cap Y\mid C\in \mathfrak {C}_G, C\cap Y\ne \emptyset \}. \end{aligned}$$

In other words, $G\wr Y$ is the restriction of *G* to the Hasse diagram of all clusters $C\cap Y$ where *C* has at least one vertex in *Y*. From a phylogenetic point of view, $G \wr Y$ does not make further assumption on the structure than what is provided by the clusters in $\mathfrak {C}_G\cap Y$.

In what follows, let $\mathbb {G}(X)$ be the set of all DAGs on *X* and $\mathbb {R}(X)$ be the set of all shortcut-free $\operatorname {LCA}$-Rel DAGs on *X*. Note that all $\operatorname {lca}$-Rel DAGs are $\operatorname {LCA}$-Rel and, therefore, Theorem 4.10 implies that the class of regular DAGs is a proper subset of $\mathbb {R}(X)$. Denote with *W*(*G*) the set of all non-$\operatorname {LCA}$ vertices in the DAG *G*, i.e., the set of all vertices $v\in V(G)$ with $v\notin \operatorname {LCA}_G(A)$ for all $A\subseteq X$. To recall, $G^-$ denotes the DAG obtained from $G\in \mathbb {G}(X)$ by removal of all shortcuts. We will show that the map

$$\begin{aligned} \varphi _{\operatorname {LCA}} :\mathbb {G}(X) \rightarrow \mathbb {R}(X) \text { defined by } \varphi _{\operatorname {LCA}}(G) = (G\ominus W(G))^- \end{aligned}$$

satisfies (P1), (P2) and (P3) when considering the cluster-restriction $G\wr Y$. Using the cluster-restriction is motivated by the fact that

$$\begin{aligned} \mathfrak {C}_G = \mathfrak {C}_{(G \ominus W(G))^-} \text { and } \varphi _{\operatorname {LCA}}(G) \simeq \mathscr {H}(\mathfrak {C}_G) \end{aligned}$$

for all DAGs satisfying (PCC) or (CL), cf. Theorem 5.8. Note that if $G \in \mathbb {R}(X)$, then *G* is a shortcut-free DAG on *X* for which $W(G) = \emptyset$. Hence, each $G \in \mathbb {R}(X)$ is already in its “simplified form” and thus satisfies $\varphi _{\operatorname {LCA}}(G) = G$. In particular, if *G* is regular, then $G = G \wr X$. In general, however, it may happen that $G \not \simeq G \wr X$. This occurs for example if *G* is not shortcut-free or if *G* contains non-LCA vertices, and thus allows for further simplifications.

**Fig. 9:**
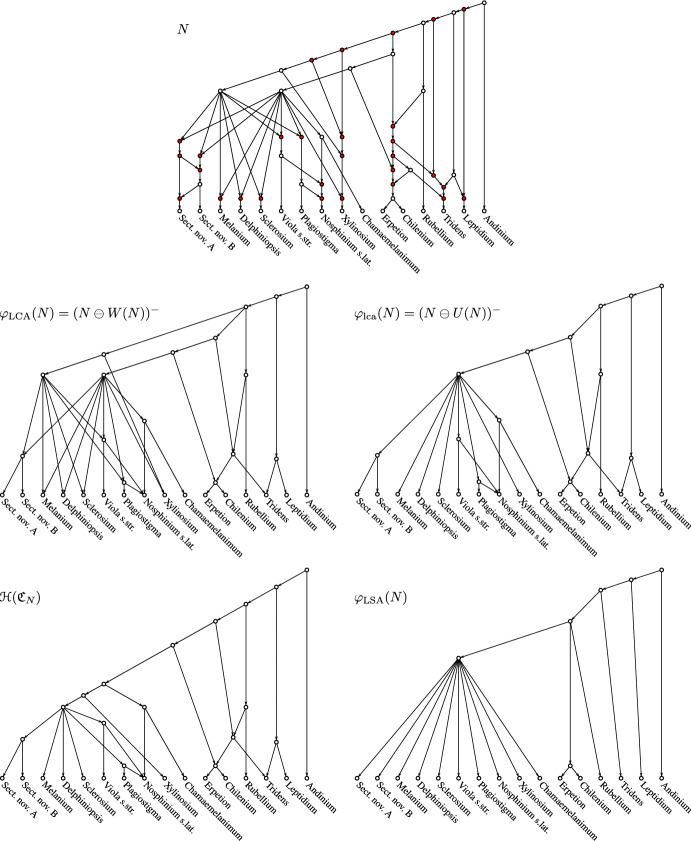
Shown is a network *N* based on a study from Marcussen et al. (2014) and adapted from Jetten and van Iersel (2018), Huson et al. (2011) together with several simplified versions: $\varphi _{\operatorname {LCA}}(N)$, $\varphi _{\operatorname {lca}}(N)$, the Hasse diagram $\mathscr {H}(\mathfrak {C}_N)$ and the ${\textrm{LSA}}$-tree $\varphi _{\textrm{LSA}}(N)$. The ${\textrm{LSA}}$-tree is adapted from (Huson et al. (2011), Fig. 3.1). Non-$\operatorname {LCA}$ vertices in *N* are highlighted in red and are comprised in the set *W*(*N*). The set *U*(*N*) comprises all non-$\operatorname {lca}$ vertices in *N*. Here, $\varphi _{\operatorname {LCA}}(N)$ satisfies (P1), (P2) and (P3). Since *N* does not satisfy (CL), $\varphi _{\operatorname {lca}}(N)$ satisfies only (P1) and (P2) and we have, therefore, $\varphi _{\operatorname {lca}}(N) \ne \mathscr {H}(\mathfrak {C}_N)$ (Color figure online)

### Proposition 6.1

The transformation $\varphi _{\operatorname {LCA}}$ satisfies (P1), (P2) and (P3) under the cluster-restriction for all DAGs. To be more precise, it holds that

1. $$G\in \mathbb {R}(X)$$ $$\implies $$ $$\varphi _{\operatorname {LCA}}(G)=G$$, and
2. $$\sigma \in \Sigma ^X$$, $$G\in \mathbb {G}(X)$$ $$\implies $$ $$\varphi _{\operatorname {LCA}}(G^\sigma )\simeq \varphi _{\operatorname {LCA}}(G)^\sigma $$, and
3. $$\emptyset \ne Y\subseteq X$$, $$G\in \mathbb {G}(X)$$ $$\implies $$ $$\varphi _{\operatorname {LCA}}(G\wr Y)\simeq \varphi _{\operatorname {LCA}}(G)\wr Y$$.

### Proof

If $G\in \mathbb {R}(X)$, then *G* is shortcut-free and $\operatorname {LCA}$-$\textsc {Rel}$ and thus, $W(G) = \emptyset$. Hence, $\varphi _{\operatorname {LCA}}(G)=(G\ominus \emptyset )^-=G^- = G$ and (P1) holds. It is a straightforward but tedious task to verify that also (P2) is satisfied, which we leave to the reader. We continue with showing (P3). Let $G\in \mathbb {G}(X)$ and $\emptyset \ne Y\subseteq X$. Since $\mathfrak {C}_G$ is grounded and *Y* is nonempty, $\mathfrak {C}_G\cap Y$ is grounded. Lemma 4.7 implies that $G\wr Y =\mathscr {H}(\mathfrak {C}_G\cap Y)$ is regular. Theorem 4.10 implies that $G\wr Y$ is shortcut-free and that $G\wr Y$ is $\operatorname {lca}$-rel and thus, in particular, $\operatorname {LCA}$-Rel which implies that $W(G\wr Y) = \emptyset$. Hence,

$$\begin{aligned} \varphi _{\operatorname {LCA}}(G\wr Y) = (G\wr Y \ominus W(G\wr Y))^- = (G\wr Y \ominus \emptyset )^- = (G\wr Y)^- = G\wr Y. \end{aligned}$$

By repeated application of Lemma 2.5 to all shortcuts of *H*, it follows that $\mathfrak {C}_H = \mathfrak {C}_{H^-}$ for every DAG *H*. This and Proposition 5.6 implies that $\mathfrak {C}_G = \mathfrak {C}_{G\ominus W(G)} = \mathfrak {C}_{(G\ominus W(G))^-}$ and, therefore, $\mathfrak {C}_{(G\ominus W(G))^-}\cap Y = \mathfrak {C}_G \cap Y$. Consequently,

$$\begin{aligned} \varphi _{\operatorname {LCA}}(G)\wr Y= &  (G\ominus W(G))^-\wr Y =\mathscr {H}(\mathfrak {C}_{(G\ominus W(G))^-}\cap Y) = \mathscr {H}(\mathfrak {C}_G \cap Y) \\ = &  G\wr Y = \varphi _{\operatorname {LCA}}(G\wr Y). \end{aligned}$$

Thus, $$\varphi _{\operatorname {LCA}}$$ satisfies (P3). $$\square $$

We now propose a second transformation which, considering the cluster-restriction, also satisfy (P1)–(P3). To this end, let $\mathbb {G}^*(X)\subseteq \mathbb {G}(X)$ be the set of all DAGs that satisfy (CL). Note that $\mathbb {G}^*(X)$ contains, in particular, all DAGs with (PCC) (cf. Lemma 4.2). For $G\in \mathbb {G}^*(X)$, let *U*(*G*) be the set of all non-$\operatorname {lca}$ vertices in *G*. Consider now the map

$$\begin{aligned} \varphi _{\operatorname {lca}} :\mathbb {G}^*(X) \rightarrow \mathbb {R}(X) \text { defined by } \varphi _{\operatorname {lca}}(G) = (G\ominus U(G))^-. \end{aligned}$$

Since all DAGs in $\mathbb {G}^*(X)$ satisfy (CL), Theorem 5.8 implies that $U(G) = W(G)$, i.e., $\varphi _{\operatorname {lca}}(G) = \varphi _{\operatorname {LCA}}(G)$. Hence, we obtain

### Proposition 6.2

The transformation $\varphi _{\operatorname {lca}}$ satisfies (P1), (P2) and (P3) under the cluster-restriction, for all DAGs with (CL) or (PCC) property. To be more precise it holds that

1. $$G\in \mathbb {R}(X)$$ $$\implies $$ $$\varphi _{\operatorname {lca}}(G)=G$$, and
2. $$\sigma \in \Sigma ^X$$, $$G\in \mathbb {G}^*(X)$$ $$\implies $$ $$\varphi _{\operatorname {lca}}(G^\sigma )\simeq \varphi _{\operatorname {lca}}(G)^\sigma $$, and
3. $$\emptyset \ne Y\subseteq X$$, $$G\in \mathbb {G}^*(X)$$ $$\implies $$ $$\varphi _{\operatorname {lca}}(G\wr Y)\simeq \varphi _{\operatorname {lca}}(G)\wr Y$$.

The transformations $\varphi _{\operatorname {LCA}}$ and $\varphi _{\operatorname {lca}}$, in particular, behave very well for DAGs in $\mathbb {G}^*(X)$ whose set of clusters exhibit a “tree-like” structure.

### Corollary 6.3

Suppose that $G\in \mathbb {G}^*(X)$ is DAG on *X* for which $\mathfrak {C}_G$ is a hierarchy, i.e., $\mathfrak {C}_G$ is a clustering system that does not contain any overlapping clusters. Then, both $\varphi _{\operatorname {LCA}}(G)$ and $\varphi _{\operatorname {lca}}(G)$ are isomorphic to the phylogenetic tree *T* on *X* with clustering system is $\mathfrak {C}_T = \mathfrak {C}_G$. In particular, the DAG *H* returned by Algorithm 3 satisfies $H^-\simeq T$.

### Proof

Let $G\in \mathbb {G}^*(X)$ be a DAG on *X* for which $\mathfrak {C}_G$ is a hierarchy. Put $H:=\varphi _{\operatorname {lca}}(G)$. Since *G* satisfies (CL), Theorem 5.8 implies that *H* is an $\operatorname {lca}$-$\textsc {Rel}$ DAG on *X*, that $\mathfrak {C}_H = \mathfrak {C}_G$ and that $H\simeq \mathscr {H}(\mathfrak {C}_H)$. Even more, $H = \varphi _{\operatorname {LCA}}(G)$ holds. Since *G* is $\operatorname {lca}$-$\textsc {Rel}$, Lemma 3.10 implies that *H* is phylogenetic. It is well-known that *T* is phylogenetic tree if and only if $\mathfrak {C}_T$ is a hierarchy and $T\doteq \mathscr {H}(\mathfrak {C}_T)$ (Semple and Steel 2003). Taking the latter arguments together, *H* is a phylogenetic tree on *X* with clustering system is $\mathfrak {C}_H = \mathfrak {C}_G$. Theorem 5.8 implies that the DAG *H* returned by Algorithm 3 satisfies $H^-\simeq T$. $\square$

We conjecture that similar results to Corollary 6.3 apply to other classes of phylogenetic networks that are characterized by properties of their clustering systems, see Hellmuth et al. (2023) for an overview. Furthermore, it would be of interest to explore in greater detail which classes of DAGs or networks $\mathbb {N}^*(X)$ ensure that $\varphi _{\operatorname {LCA}}(G)$ or $\varphi _{\operatorname {lca}}(G)$ remain in $\mathbb {N}^*(X)$ whenever $G\in \mathbb {N}^*(X)$.

A simple example of the application of $\varphi _{\operatorname {LCA}}$ and $\varphi _{\operatorname {lca}}$ to a network is shown in Fig. 8. While the transformation $\varphi _{\operatorname {LCA}}$ applied to any DAG satisfies (P1), (P2) and (P3), it is ensured that the transformation $\varphi _{\operatorname {lca}}$ satisfies (P1), (P2) and (P3) for DAGs in $\mathbb {G}^*(X)$ and thus, only for DAGs with the (CL) property. In general, $\varphi _{\operatorname {lca}}$ does not satisfy (P3). By way of example, consider the network *G* on $X=\{x_1,\ldots ,x_n\}$ in Fig. 6 for some $n\ge 2$, where $U(G)=V(G){\setminus } X$. Consequently, $G':=(G\ominus U(G))^-$ is the DAG $(X,\emptyset )$ with no edges or inner vertices. Restricting the DAG $G'$ to, say, $Y=\{x_1,x_2\}$ thus also yield a DAG $G'\wr Y=\mathscr {H}(\mathfrak {C}_{G'}\cap Y)=\mathscr {H}(\{\{x_1\},\{x_2\}\})$ without edges. In contrast, we have

$$\begin{aligned}\varphi _{\operatorname {lca}}(G\wr Y)=\varphi _{\operatorname {lca}}(\mathscr {H}(\mathfrak {C}_G\cap Y))=\mathscr {H}(\{\{x_1\},\{x_2\},\{x_1,x_2\}\}),\end{aligned}$$

thus $\varphi _{\operatorname {lca}}(G)\wr Y\ne \varphi _{\operatorname {lca}}(G\wr Y)$. Nevertheless, the application of $\varphi _{\operatorname {lca}}$ to DAGs in $\mathbb {G}(X)\setminus \mathbb {G}^*(X)$ can reveal meaningful insights, cf. $\varphi _{\operatorname {lca}}(N)$ in Fig. 9 which is distinct from $\varphi _{\operatorname {lca}}(N\wr L(N)) = \mathscr {H}(\mathfrak {C}_N)$.

## Computational Complexity Results for General and (N3O) DAGs

In Sect. 5, we have shown that it is possible to compute $\mathscr {I}^{1}$-$\operatorname {lca}$-Rel and $\mathscr {I}^{1}$-$\operatorname {LCA}$-Rel DAGs in polynomial time by stepwise removal of certain vertices using the $\ominus$-operator, given that $\mathscr {I}^{1}= \{1, \dots , |X|\}$. However, this situation becomes more challenging when $\mathscr {I}^{1}\subsetneq \{1, \dots , |X|\}$. As we shall see, determining as whether a vertex is a ${{\,\mathrm{\textit{k}-lca}\,}}$ or ${{\,\mathrm{\textit{k}-LCA}\,}}$ or verifying that a DAG or network is $\mathscr {I}^{1}$-$\operatorname {lca}$-Rel or $\mathscr {I}^{1}$-$\operatorname {LCA}$-Rel are, in general, NP-hard tasks. Nevertheless we provide polynomial time algorithms for the latter tasks for DAGs *G* whose set system $\mathfrak {C}_G$ satisfies (N3O), i.e., $\mathfrak {C}_G$ does not contain three distinct pairwise overlapping clusters.

### General DAGs

For the NP-hardness proofs, we use reductions from the well-known “Vertex Cover Problem”, which is based on undirected graphs *H* where the edge set consists—unlike in directed graphs—of two-element subsets of *V*(*H*).

#### Problem

(*Vertex Cover*) 
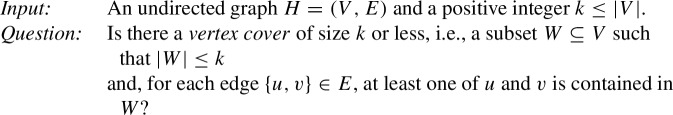


#### Theorem 7.1

(Garey and Johnson 1979) *Vertex Cover* is NP-complete.

For our NP-hardness proofs below we require that the graph $H=(V,E)$ and the integer *k* that serve as input for the problem *Vertex Cover* satisfies certain constrains. To this end, we provide the following simple observation which is a direct consequence of the fact that *W* is a vertex cover of an instance (*H*, *k*) if and only if $W' = W\cup \{v\}$ is a vertex cover of an instance $(H',k')$ obtained from *H* by adding new vertices *u*, *v*, *w* and edges $\{v,u\}$ and $\{v,w\}$ and by putting $k'=k+1 > 1$.

#### Observation 7.2

*Vertex Cover* remains NP-complete if the input is restricted to $k>1$ and undirected graphs $H=(V,E)$ such that $|V|\ge 4$, $|E|\ge 2$ and *H* is not star-graph, i.e., a connected graph which contains a unique vertex that is contained in all edges.

For the upcoming proofs, the following simple result will come in handy.

#### Lemma 7.3

A subset $W\subseteq V$ is a vertex cover of $H = (V,E)$ if and only if $W\not \subseteq V{\setminus } \{u,v\}$ for all $\{u,v\}\in E$.

#### Proof

If $W \subseteq V\setminus \{u,v\}$ for some $\{u,v\}\in E$, then it can clearly be no vertex cover. Conversely, if $W\subseteq V$ is not a vertex cover of *H*, then there is some edge $\{u,v\}\in E$ such that $u,v\notin W$. This together with $W \subseteq V$ implies $W \subseteq V\setminus \{u,v\}$. $\square$

We now formally state the decision problems whose NP-completeness we intend to prove.

#### Problem

(${k}$-*lca* (resp., ${k}$-*LCA*)) 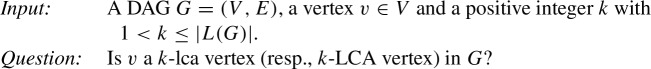


#### Problem

(${\mathscr {I}^{1}}$-*lca*-*Rel* (resp., ${\mathscr {I}^{1}}$-*LCA*-*Rel*)) 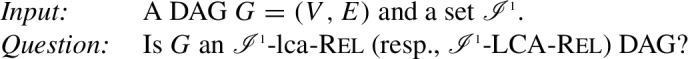


We start with the three problems ${k}$-*lca*, ${k}$-*LCA* and ${\mathscr {I}^{1}}$-*LCA*-*Rel*.

#### Theorem 7.4

The problems ${k}$-*LCA*, ${k}$-*lca* and ${\mathscr {I}^{1}}$-*LCA*-*Rel* are NP-complete, even if the input DAG *G* is a regular network and, thus satisfies (PCC), strong-(CL) and is $\operatorname {lca}$-Rel and shortcut-free.

#### Proof

To see that ${k}$-*LCA* and ${k}$-*lca* are in $\textrm{NP}$, let $A\subseteq X$ of size $k=|A|$ be a given certificate. Now apply Corollary 3.12. To see that ${\mathscr {I}^{1}}$-*LCA*-*Rel* is in $\textrm{NP}$, we assume that as a certificate, we have for each vertex $v \in V(G)$ a subset $A_v \subseteq X$ with $|A_v| \in \mathscr {I}^{1}$. Verifying whether $v \in \operatorname {LCA}_G(A_v)$ can be done in polynomial time due to Corollary 3.12.

To prove NP-hardness, we use a reduction from *Vertex Cover*. Let (*H*, *k*) be an arbitrary instance of *Vertex Cover*. By Observation 7.2, we can assume that $k>1$, $|V(H)|\ge 4$, $|E(H)|\ge 2$ and that *H* is not a star-graph. Consider the following set system

$$\begin{aligned}\mathfrak {C} :=\left( \bigcup _{x\in V(H)}\{\{x\}\}\right) \cup \left( \bigcup _{e\in E(H)} \{V(H)\setminus e\}\right) \cup \{V(H)\}.\end{aligned}$$

**Fig. 10:**
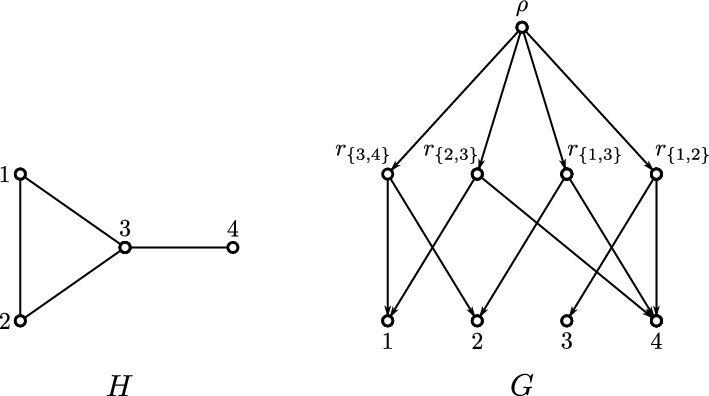
Reduction used in the proof of Theorem 7.4. In this example, *H* and $k=2$ serve as input for *Vertex Cover*. Here, $W=\{1,3\}$ and $W=\{2,3\}$ are the only vertex covers of *H* size $|W|\le 2$. Moreover, $\rho$ is a *k*-$\operatorname {lca}$, resp., *k*-$\operatorname {LCA}$ vertex for $k=2$ precisely for the two sets $W=\{1,3\}$ and $W=\{2,3\}$. In particular, $\rho$ is a *k*-$\operatorname {lca}$, resp., *k*-$\operatorname {LCA}$ in *G* precisely if *H* has a vertex cover of size *k*. We note that, in general, the vertices $r_e$ are not 2-$\operatorname {lca}$ vertices but always 2-$\operatorname {LCA}$ vertices

Since $|V(H)|\ge 4$, we have $|V(H)\setminus e|\ge 2$ for all $e\in E(H)$. Thus, $V(H){\setminus } e$ appears as a non-singleton cluster in $\mathfrak {C}$. It is now easy to verify that $\mathfrak {C}$ is a well-defined clustering system. Let $G\doteq \mathscr {H}(\mathfrak {C})$ be the DAG obtained from the Hasse diagram $\mathscr {H}(\mathfrak {C})$ by relabeling all vertices $\{x\}$ by *x*. Hence, $L(G) = V(H)$ and $\mathfrak {C}_G = \mathfrak {C}$. Since $\mathfrak {C}$ is a clustering system and $G\simeq \mathscr {H}(\mathfrak {C}_G)$, Lemma 4.7 implies that *G* is a regular network. By Theorem 4.10, *G* is shortcut-free, $\operatorname {lca}$-$\textsc {Rel}$ and satisfies the strong-(CL) property. An example of the constructed DAG *G* is shown in Fig. 10.

We show first NP-hardness of ${k}$-*LCA* and ${k}$-*lca*. Let us denote with $\rho$ the unique root of *G* which results in the instance $(G,\rho ,k)$ of ${k}$-*lca*, as well as of ${k}$-*LCA*. By definition, the vertex set of *G* consists of the unique root $\rho$, the leaves in $L(G)=V(H)$ and a vertex $r_e$ that correspond to the cluster $V(H)\setminus e$ for each $e\in E(H)$. Note that $r_e$ does not correspond to a leaf in *G* since $V(H)\setminus e$ is not a singleton cluster in $\mathfrak {C}$ and, since $|E(H)|\ge 2$, at least two such vertices $r_e$ with $e\in E(H)$ exist. Moreover, since *H* is not a star-graph, for each vertex $v\in V(H)$ there is an edge $e\in E(H)$ such $v\notin e$. Hence, each vertex $v\in V(H)$ is contained in at least one set $V(H)\setminus e$ for some $e\in E(H)$. The latter arguments imply that *G* has edges $(\rho ,r_e)$ for all $e\in E(H)$ and edges $(r_e,v)$ for all $e\in E(H)$ and all vertices $v \in V(H){\setminus } e$. No further edges exists. Thus, without explicit construction of the Hasse diagram based on the clustering system $\mathfrak {C}$, we can instead directly construct *G* by adding $1 + |E(H)| + |V(H)|$ vertices to *G* and the prescribed $|E(H)| + |E(H)|(|V(H)|-2)$ edges to *G*. In summary, *G* and thus, the instance $(G,\rho ,k)$ of ${k}$-*lca* and ${k}$-*LCA*, can be constructed in polynomial time.

Suppose that (*H*, *k*) is a yes-instance of *Vertex Cover*. Thus, there is, in particular, a vertex cover *W* of *H* such that $|W|=k>1$. By construction, $\rho$ is a common ancestor of all vertices in $L(G) = V(H)$ and thus, of all vertices in *W*. The vertex $r_e$ is a common ancestor of $A\subseteq L(G) = V(H)$ precisely if $A\subseteq V(H){\setminus } e$ and $|A|> 1$. By Lemma 7.3, $W\not \subseteq V(H)\setminus e$ and thus, $r_e$ is not a common ancestor of *W* for any $e\in E(H)$. Thus, $\rho$ is the unique least common ancestor of *W* and therefore, $\rho = \operatorname {lca}_G(W)$. Since $|W|=k$, the root $\rho$ is a ${{\,\mathrm{\textit{k}-lca}\,}}$ vertex and thus, in particular, a ${{\,\mathrm{\textit{k}-LCA}\,}}$ vertex.

Suppose that $(G,\rho ,k)$ is a yes-instance of ${k}$-*lca* (resp., ${k}$-*LCA*). Hence, $\rho = \operatorname {lca}_G(W)$ (resp., $\rho \in \operatorname {LCA}_G(W)$) for some $W\subseteq L(G) = V(H)$ with $|W|=k>1$. If $W\subseteq V(H)\setminus e$ for some edge $e\in E(H)$, then $r_e$ is the unique least common ancestor of *W*; contradicting $\rho = \operatorname {lca}_G(W)$ (resp. $\rho \in \operatorname {LCA}_G(W)$). Thus, it must hold that $W\not \subseteq V(H){\setminus } e$ for all $e\in E(H)$ and Lemma 7.3 implies that *W* is a vertex cover of *H*. In summary, ${k}$-*lca* and ${k}$-*LCA* are NP-hard and, therefore, NP-complete.

To show NP-hardness of ${\mathscr {I}^{1}}$-*LCA*-*Rel*, we use the same DAG *G* and put $\mathscr {I}^{1}= \{1,2,k\}$ which results in an instance $(G,\mathscr {I}^{1})$ of ${\mathscr {I}^{1}}$-*LCA*-*Rel*. By the arguments above, this reduction can be achieved in polynomial time. As shown above, $\rho$ is a ${{\,\mathrm{\textit{k}-LCA}\,}}$ vertex, that is $\rho \in \operatorname {LCA}_G(W)$ for some $W\subseteq L(G)=V(H)$ with $W=k$ if and only if *W* is a vertex cover of *H*. Moreover, since distinct $r_e$ and $r_f$ are $\preceq _G$-incomparable and since each $r_e$ is adjacent to at least two leaves $x,y\in V(H)\setminus e$, it follows that $r_e \in \operatorname {LCA}_G(\{x,y\})$, i.e., $r_e$ is a 2-$\operatorname {LCA}$ vertex for each $e\in E(H)$. In summary, (*H*, *k*) is a yes-instance of *Vertex Cover* if and only if $(G,\mathscr {I}^{1})$ is a yes-instance of ${\mathscr {I}^{1}}$-*LCA*-*Rel*. Therefore, ${\mathscr {I}^{1}}$-*LCA*-*Rel* is NP-hard and thus, NP-complete. $\square$

The NP-hardness of ${k}$-*lca* and ${\mathscr {I}^{1}}$-*LCA*-*Rel* does not directly imply that ${\mathscr {I}^{1}}$-*lca*-*Rel* is NP-hard as well. In particular, the vertices $r_e$ in the instance *G* constructed in Theorem 7.4 are, in general, not 2-$\operatorname {lca}$ vertices. Moreover, verifying whether *G* is $\mathscr {I}^{1}$-$\operatorname {lca}$-Rel is equivalent to checking that *all* vertices $v \in V(G)$ are $\mathscr {I}^{1}$-$\operatorname {lca}$ vertices, which imposes strong structural constraints on *G*. Nevertheless, it is not at all surprising that we arrive at the following

#### Theorem 7.5

The problem ${\mathscr {I}^{1}}$-*lca*-*Rel* is NP-complete.

#### Proof

**Fig. 11:**
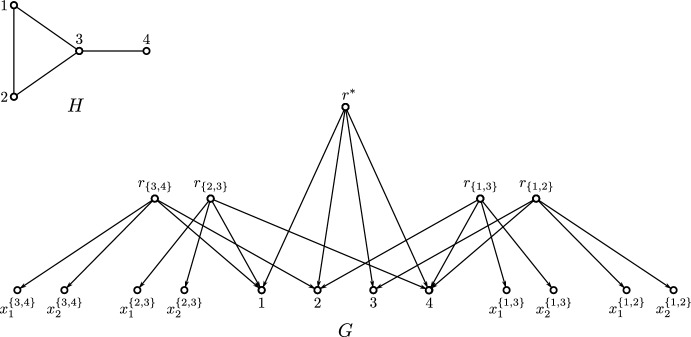
Reduction used in the proof of Theorem 7.5. In this example, *H* and $k=2$ serve as input for *Vertex Cover*. Here, $W=\{1,3\}$ and $W=\{2,3\}$ are the only vertex covers of *H* size $|W|\le 2$. Each vertex $v\ne r^*$ in *G* is, by construction, a $\{1,2\}$-$\operatorname {lca}$ vertex: if *v* is a leaf it is a 1-$\operatorname {lca}$ vertex and, otherwise, $r_e=\operatorname {lca}_G(\{x^e_1, x^e_2\})$ with $e\in E(H)$ is a 2-$\operatorname {lca}$ vertex. Moreover, $r^*$ is a *k*-$\operatorname {lca}$ vertex for $k=2$ precisely for the two sets $W=\{1,3\}$ and $W=\{2,3\}$. In particular, *G* is a $\{1,2,k\}$-$\operatorname {lca}$-Rel precisely if *H* has a vertex cover of size *k*

Showing that ${\mathscr {I}^{1}}$-*lca*-*Rel* is in $\textrm{NP}$ is done in the same way as showing that ${\mathscr {I}^{1}}$-*LCA*-*Rel* is in $\textrm{NP}$. To prove NP-hardness, we use a reduction from *Vertex Cover*. Let (*H*, *k*) be an arbitrary instance of *Vertex Cover*. By Observation 7.2, we can assume that $k>1$, $|V(H)|\ge 4$ and $|E(H)|\ge 2$. We construct now an instance $(G,\mathscr {I}^{1})$ for ${\mathscr {I}^{1}}$-*lca*-*Rel*. First, put $\mathscr {I}^{1}= \{1,2,k\}$. Now construct a DAG *G* as follows. First, initialize *G* as the Hasse diagram $G\doteq \mathscr {H}(\mathfrak {C})$ of the set system $\mathfrak {C} :=(\bigcup _{x\in V(H)}\{\{x\}\}) \cup (\cup _{e\in E(H)} \{V(H){\setminus } e\})$. Here, *G* is equivalently obtained from the DAG constructed in the proof of Theorem 7.4 by removal of the unique root $\rho$. For each $e\in E(H)$, let $r_e$ denote the vertex in *G* that is adjacent to all $x\in V(H){\setminus } e$. We now add to *G*, for all $e\in E(H)$, two new leaves $x^e_1, x^e_2$ and edges $(r_e,x^e_1)$ and $(r_e,x^e_2)$. Let us denote with *Z* the set comprising all leaves $x^e_1$ and $x^e_2$ for all $e\in E(H)$. Finally add to *G* a vertex $r^*$ and edges $(r^*,x)$ for all $x\in V(H)$. By construction, $L(G) = V(H)\cup Z$. This results in the instance $(G,\mathscr {I}^{1})$ for ${\mathscr {I}^{1}}$-*lca*-*Rel*, see Fig. 11 for an illustrative example. By similar arguments as in the proof of Theorem 7.4, the instance $(G,\mathscr {I}^{1})$ can be constructed in polynomial time.

By construction, each $r_e$ in *G* is precisely adjacent to the leaves in $V(H) \setminus e$ and the two leaves $x^e_1$ and $x^e_2$. In particular, $r_e$ is the only ancestor of the leaves $x^e_1$ and $x^e_2$. Hence, $r_e=\operatorname {lca}_G(\{x^e_1,x^e_2\})$ which implies that $r_e$ is a 2-$\operatorname {lca}$ vertex for all $e\in E(H)$. Therefore, it is evident that $(G,\mathscr {I}^{1})$ is a yes-instance of ${\mathscr {I}^{1}}$-*lca*-*Rel* with $\mathscr {I}^{1}= \{1,2,k\}$ if and only if $r^*$ is a $\{2,k\}$-$\operatorname {lca}$ vertex in *G*. Although the DAG *G* constructed here slightly differs from the one in the proof Theorem 7.4 (in particular, $r^*$ is not an ancestor of any $r_e$ with $e\in E(H)$), $r^*$ is still an ancestor of all $x\in V(H) = L(G)\setminus Z$ and of no other vertices.

Suppose that (*H*, *k*) is a yes-instance of *Vertex Cover*. Thus, there is a vertex cover $W\subseteq V(H)$ of *H* such that $|W|=k>1$. By construction, $r^*$ is a common ancestor of all vertices in $V(H) = L(G) \setminus Z$ and thus, of all vertices in *W*, but not of $r_e$ for all $e\in E(H)$. Since $W \subseteq V(H)$, the vertex $r_e$ is a common ancestor of *W* precisely if $W\subseteq V(H)\setminus e$. However, by Lemma 7.3, for all $e\in E(H)$, $W\not \subseteq V(H){\setminus } e$ and thus, $r_e$ is not a common ancestor of *W*. Thus, $r^*$ is the unique least common ancestor of *W* and therefore, $r^* = \operatorname {lca}_G(W)$. Since $|W|=k$, the vertex $r^*$ is a ${{\,\mathrm{\textit{k}-lca}\,}}$ vertex. In summary, $(G,\mathscr {I}^{1})$ is a yes-instance of ${\mathscr {I}^{1}}$-*lca*-*Rel*.

Suppose that $(G,\mathscr {I}^{1})$ is a yes-instance of ${\mathscr {I}^{1}}$-*lca*-*Rel*. Hence, $r^* = \operatorname {lca}_G(W)$ for some $W\subseteq L(G) = V(H)\cup Z$ with $|W|\in \{2,k\}$. Since $r^*$ is not an ancestor of any vertex in *Z*, it follows that $W\subseteq V(H)$. If $W\subseteq V(H){\setminus } e$ for some edge $e\in E(H)$, then $r_e$ is a common ancestor of *W*. Since $r_e$ and $r^*$ are $\preceq _G$-incomparable, this would contradict $r^* = \operatorname {lca}_G(W)$. Thus, it must hold that $W\not \subseteq V(H){\setminus } e$ for all $e\in E(H)$ and Lemma 7.3 implies that *W* is a vertex cover of *H*. Hence, (*H*, *k*) is a yes-instance of *Vertex Cover*. Thus, ${\mathscr {I}^{1}}$-*lca*-*Rel* is NP-hard and, consequently, NP-complete. $\square$

Although the problems ${k}$-*lca* and ${k}$-*LCA* as well as ${\mathscr {I}^{1}}$-*lca*-*Rel* and ${\mathscr {I}^{1}}$-*LCA*-*Rel* are NP-hard, we note in passing that these problems become polynomial-time solvable whenever *k* is treated as a constant, i.e., $k\in O(1)$. To test if a vertex *v* is a ${{\,\mathrm{\textit{k}-lca}\,}}$ or a ${{\,\mathrm{\textit{k}-LCA}\,}}$ vertex in a DAG $G=(V,E)$ can then be done by testing all $O(|V|^k)$ subsets $A \subseteq {{\,\mathrm{\texttt{C}}\,}}_G(v)$ with $|A| = k$ using Algorithm 1. Hence, testing if *v* is ${{\,\mathrm{\textit{k}-lca}\,}}$ or ${{\,\mathrm{\textit{k}-LCA}\,}}$ can be done in polynomial time. Similarly, if in a given set $\mathscr {I}^{1}$ the maximum integer $k\in \mathscr {I}^{1}$ is treated as fixed and constant, we can repeat the latter arguments for all vertices in *G* and all $O(|V|^k)$ subsets of size at most *k* to obtain a polynomial time approach for verifying if *G* is $\mathscr {I}^{1}$-$\operatorname {lca}$-Rel or $\mathscr {I}^{1}$-$\operatorname {LCA}$-Rel or not. This approach is, in particular, feasible when we are interested in the special case that all $v\in V$ satisfy $v = \operatorname {lca}(\{x, y\})$ or $v \in \operatorname {LCA}(\{x, y\})$ for some $x, y \in X$. This discussion together with Proposition 5.6 and 5.7 implies

#### Observation 7.6

It can be tested in polynomial time if a vertex $v\in V$ in a DAG $G=(V,E)$ is a $\{1,2\}$-$\operatorname {lca}$ or $\{1,2\}$-$\operatorname {LCA}$ vertex. Moreover, it can be tested in polynomial time if a DAG is $\{1,2\}$-$\operatorname {lca}$-Rel or $\{1,2\}$-$\operatorname {LCA}$-Rel. Finally, every DAG *G* can be transformed in polynomial-time into a phylogenetic $\{1,2\}$-$\operatorname {lca}$-Rel (resp., $\{1,2\}$-$\operatorname {LCA}$-Rel) DAG satisfying (S0)–(S4) (resp., (S0)–(S5)) w.r.t. *G* using straightforward modifications of Algorithm 3 (resp., Algorithm 2).

We now summarize the complexity results presented in this paper so far. We have shown that it is tractable to decide whether a given vertex is the (unique) LCA of a *specified set* *A* (cf. Corollary 3.12) and whether a given vertex *v* is a *k*-$\operatorname {lca}$ (resp. *k*-$\operatorname {LCA}$) vertex for *some* integer *k* (cf. Corollaries 3.12 and 3.6). However, if *k* is is part of the input and not treated as constant, it is NP-complete to determine whether *v* is a *k*-$\operatorname {lca}$ or a *k*-$\operatorname {LCA}$ vertex. Similarly, deciding whether a DAG is $\mathscr {I}^{1}$-$\operatorname {lca}$-$\textsc {Rel}$ (resp. $\mathscr {I}^{1}$-$\operatorname {LCA}$-$\textsc {Rel}$) is tractable when $\mathscr {I}^{1}=\{1,\ldots ,|L(G)|\}$ (cf. Corollary 3.12) but NP-complete otherwise.

### DAGs with (N3O) property

Although the problems ${\mathscr {I}^{1}}$-*lca*-*Rel* and ${\mathscr {I}^{1}}$-*LCA*-*Rel*, as well as ${k}$-*LCA* and ${k}$-*lca*, are NP-complete, we show that they can be efficiently solved when the input DAGs *G* *satisfy (N3O)*, i.e., $\mathfrak {C}_G$ satisfies (N3O) and thus does not contain three distinct pairwise overlapping clusters. The interest into DAGs that satisfy (N3O) is two-fold. On the one hand, DAGs with (N3O) property include interesting and non-trivial classes of networks such as rooted trees or galled-trees (Hellmuth et al. 2023), i,e., networks in which each connected component *K* is either a single vertex, an edge or *K* is composted of exactly two *uv*-paths that only have *u* and *v* in common. An example of a galled-tree is provided by the network $N_1$ in Fig. 2. In particular, galled-trees form a subclass of level-1 networks, i.e., networks *N* in which each biconnected component contains at most one vertex *v* with ${{\,\textrm{indeg}\,}}_N(v)>1$ (Hellmuth et al. 2023). On the other hand, it can be verified in polynomial time whether a given set system $\mathfrak {C}$ on *X* satisfies (N3O) or not, by checking, for all of the $O(|\mathfrak {C}|^3)$ distinct clusters $C_1,C_2,C_3\in \mathfrak {C}$, if they pairwise overlap or not in $O(|X|^3)$ time. Since $|\mathfrak {C}_G|\le |V|$ and since $\mathfrak {C}_G$ can, together with Lemma 2.2, be determined by a simple post-order traversal in polynomial time for every DAG *G*, this yields a polynomial time approach to test if *G* satisfies (N3O).

#### Observation 7.7

DAGs with (N3O) property can be recognized in polynomial time.

In what follows, we show that the problems ${\mathscr {I}^{1}}$-*lca*-*Rel* and ${\mathscr {I}^{1}}$-*LCA*-*Rel*, as well as ${k}$-*LCA* and ${k}$-*lca* can be solved in polynomial time on DAGs with (N3O) property. To this end, we start with the following results.

#### Lemma 7.8

Let *G* be a DAG on *X* that satisfies (N3O) and let $v\in V(G)$. Then, *v* is a ${{\,\mathrm{\textit{2}-LCA}\,}}$ vertex in *G* if and only if (a) $|{{\,\mathrm{\texttt{C}}\,}}_G(v)|\ge 2$ and (b) ${{\,\mathrm{\texttt{C}}\,}}_G(u)\ne {{\,\mathrm{\texttt{C}}\,}}_G(v)$ for all $u\in {{\,\textrm{child}\,}}_G(v)$. In particular, the following statements are equivalent.

1. *v* is a ${{\,\mathrm{\textit{k}-LCA}\,}}$ vertex in *G* for some $k\ge 2$.
2. *v* is an $\ell$-$\operatorname {LCA}$ vertex in *G* for all $\ell \in \{2,3,\dots ,|{{\,\mathrm{\texttt{C}}\,}}_G(v)|\}$.

Hence, *G* is $\mathscr {I}^{1}$-$\operatorname {LCA}$-Rel for some $\mathscr {I}^{1}$ with $|\mathscr {I}^{1}|>1$ if and only if each inner vertex of *G* is a ${{\,\mathrm{\textit{2}-LCA}\,}}$ vertex.

#### Proof

Let *G* be a DAG on *X* that satisfies (N3O) and let $v\in V(G)$. First suppose that *v* is a ${{\,\mathrm{\textit{2}-LCA}\,}}$ vertex in *G* and thus that $v\in \operatorname {LCA}_G(A)$ for some $A\subseteq X$ with $|A|=2$. Hence, $A\subseteq {{\,\mathrm{\texttt{C}}\,}}_G(v)$ and $|{{\,\mathrm{\texttt{C}}\,}}_G(v)|\ge 2$ must hold. By Lemma 3.1, ${{\,\mathrm{\texttt{C}}\,}}_G(u)\ne {{\,\mathrm{\texttt{C}}\,}}_G(v)$ for all $u\in {{\,\textrm{child}\,}}_G(v)$. Conversely, assume that Condition (a) and (b) are satisfied for *v*. Assume, for contradiction, that *v* is not a ${{\,\mathrm{\textit{2}-LCA}\,}}$ vertex. Let $\mathfrak {C}^*$ be the set of all inclusion-maximal cluster in the set $\{{{\,\mathrm{\texttt{C}}\,}}_G(u)\mid u\in {{\,\textrm{child}\,}}_G(v)\}$ of the clusters associated with the children of *v* in *G*. Since $|{{\,\mathrm{\texttt{C}}\,}}_G(v)|\ge 2$, *v* must be an inner vertex and Lemma 2.2 implies that

I $$\begin{aligned} {{\,\mathrm{\texttt{C}}\,}}_G(v)=\bigcup _{u\in {{\,\textrm{child}\,}}_G(v)}{{\,\mathrm{\texttt{C}}\,}}_G(u)=\bigcup _{C\in \mathfrak {C}^*}C. \end{aligned}$$

Since *v* is not a leaf and not a ${{\,\mathrm{\textit{2}-LCA}\,}}$ vertex, Lemma 3.1 implies that, for all $A\subseteq {{\,\mathrm{\texttt{C}}\,}}_G(v)$ with $|A|=2$, there must be some child $u\in {{\,\textrm{child}\,}}_G(v)$ such that $A\subseteq {{\,\mathrm{\texttt{C}}\,}}_G(u)$. Thus, for every $A\subseteq {{\,\mathrm{\texttt{C}}\,}}_G(v)$ of size $|A|=2$ there is, in particular, some element $C\in \mathfrak {C}^*$ such that $A\subseteq C$. Note that any two clusters in $\mathfrak {C}^*$ are either disjoint or overlap. Condition (b) and Eq. (I) imply that $|\mathfrak {C}^*|\ge 2$. Assume, first that the clusters in $\mathfrak {C}^*$ are pairwise disjoint. Let $C_1,C_2$ be distinct elements in $\mathfrak {C}^*$ and $x\in C_1$ and $y\in C_2$. Since $C_1\cap C_2=\emptyset$, we have $x\ne y$. However, as argued above, there exists a cluster $C_3\in \mathfrak {C}^*$ such that $\{x,y\}\subseteq C_3$ and, thus, $C_1\cap C_3\ne \emptyset$; a contradiction. Hence, the clusters in $\mathfrak {C}^*$ cannot all be pairwise disjoint. Thus, there are clusters $C_1,C_2 \in \mathfrak {C}^*$ that overlap. Therefore, there are $x\in C_1{\setminus } C_2$ and $y\in C_2{\setminus } C_1$ and thus, $x\ne y$. Again, as argued above, there exists a cluster $C_3\in \mathfrak {C}^*$ such that $\{x,y\}\subseteq C_3$ and thus, $C_3\ne C_1,C_2$. Consequently, $C_3$ overlaps with both $C_1$ and $C_2$, i.e., $\mathfrak {C}^*$ contains the three pairwise overlapping clusters $C_1,C_2$ and $C_3$; violating the fact that *G* satisfies (N3O). Hence, this case cannot occur. In summary, *v* must be a ${{\,\mathrm{\textit{2}-LCA}\,}}$ vertex.

We show now that Statements (1) and (2) are equivalent. Clearly (2) implies (1). Hence, assume that *v* is a ${{\,\mathrm{\textit{k}-LCA}\,}}$ vertex in *G* for some $k\ge 2$. One easily observes that $k\le |{{\,\mathrm{\texttt{C}}\,}}_G(v)|$ must hold as, otherwise, there is no $A\subseteq X$ of size $|A|=k$ such $A\subseteq {{\,\mathrm{\texttt{C}}\,}}_G(v)$ and, thus $v\notin \operatorname {LCA}_G(A)$ by Lemma 3.1. Moreover, there cannot be a child *u* of *v* in *G* such that ${{\,\mathrm{\texttt{C}}\,}}_G(u)={{\,\mathrm{\texttt{C}}\,}}_G(v)$ since, otherwise, Lemma 3.1 would imply that *v* is not a *k*-$\operatorname {LCA}$ vertex. Thus, we have shown that (a) $|{{\,\mathrm{\texttt{C}}\,}}_G(v)|\ge k \ge 2$ and (b) ${{\,\mathrm{\texttt{C}}\,}}_G(u)\ne {{\,\mathrm{\texttt{C}}\,}}_G(v)$ for all $u\in {{\,\textrm{child}\,}}_G(v)$ must hold. By the previous statement, *v* is a ${{\,\mathrm{\textit{2}-LCA}\,}}$ vertex. By Lemma 3.3, *v* is an $\ell$-$\operatorname {LCA}$ vertex in *G* for all $\ell$ with $2\le \ell \le |{{\,\mathrm{\texttt{C}}\,}}_G(v)|$.

It is now an easy task to verify that *G* is $\mathscr {I}^{1}$-$\operatorname {LCA}$-Rel for some $\mathscr {I}^{1}$ with $|\mathscr {I}^{1}|>1$ (which implies that $\ell \in \mathscr {I}^{1}$ for some $\ell >1$) if and only if each inner vertex of *G* is a ${{\,\mathrm{\textit{2}-LCA}\,}}$ vertex. $\square$

#### Lemma 7.9

Let *G* be a DAG on *X* that satisfies (N3O) and let $v\in V(G)$. Then, the following statements are equivalent.

1. *v* is a ${{\,\mathrm{\textit{k}-lca}\,}}$ vertex in *G* for some $k\ge 2$.
2. *v* is an $\ell$-$\operatorname {lca}$ vertex in *G* for all $\ell \in \{2,3,\dots ,|{{\,\mathrm{\texttt{C}}\,}}_G(v)|\}$.

Moreover, the following statements are equivalent.

(1): *G* is $\mathscr {I}^{1}$-$\operatorname {lca}$-Rel for some $\mathscr {I}^{1}$ with $|\mathscr {I}^{1}|>1$.
(2): Each inner vertex of *G* is a 2-$\operatorname {lca}$ vertex.
(3): *G* has the strong-(CL) property.

#### Proof

Suppose first that *v* is a ${{\,\mathrm{\textit{k}-lca}\,}}$ vertex in *G* for some $k\ge 3$. We start with showing that *v* is a $(k-1)$-$\operatorname {lca}$ vertex in *G*. Since *v* is a ${{\,\mathrm{\textit{k}-lca}\,}}$ vertex, there is a subset $A \subseteq X$ of size $|A|=k$ such that $\operatorname {lca}_G(A)=v$. Assume, for contradiction, that *v* is not a $(k-1)$-$\operatorname {lca}$ vertex in *G*. Since $|A|\ge 3$ we can choose three distinct vertices $x_1,x_2,x_3\in A$. Put $A_i:=A{\setminus } \{x_i\}$ for all $i\in \{1,2,3\}$. Note that $|A_i|=k-1$ and thus, $v\ne \operatorname {lca}_G(A_i)$ for all $i\in \{1,2,3\}$. Since *v* is a common ancestor of every vertex in $A_i$ but $v\ne \operatorname {lca}_G(A_i)$ it follows that there is a vertex $v_i\prec _G v$ with $v_i\in \operatorname {LCA}_G(A_i)$ for all $i\in \{1,2,3\}$ By construction and since $|A|\ge 3$, $A_i\cap A_j\ne \emptyset$ and thus, ${{\,\mathrm{\texttt{C}}\,}}_G(v_i)\cap {{\,\mathrm{\texttt{C}}\,}}_G(v_j)\ne \emptyset$ for all $i,j\in \{1,2,3\}$. Since $v_i \prec _G v=\operatorname {lca}_G(A)$ and $A_i\subseteq {{\,\mathrm{\texttt{C}}\,}}_G(v_i)$ and $A_i\cup \{x_i\}=A$ it follows that $x_i\notin {{\,\mathrm{\texttt{C}}\,}}_G(v_i)$ for all $i\in \{1,2,3\}$. By construction, $x_i \in {{\,\mathrm{\texttt{C}}\,}}_G(v_j)$ for all distinct $i,j\in \{1,2,3\}$. Hence, ${{\,\mathrm{\texttt{C}}\,}}_G(v_i)\not \subseteq {{\,\mathrm{\texttt{C}}\,}}_G(v_j)$ for all distinct $i,j\in \{1,2,3\}$. Consequently, ${{\,\mathrm{\texttt{C}}\,}}_G(v_1)$, ${{\,\mathrm{\texttt{C}}\,}}_G(v_2)$ and ${{\,\mathrm{\texttt{C}}\,}}_G(v_3)$ are three pairwise overlapping cluster; contradicting the fact that *G* satisfied (N3O). Hence, *v* is a $(k-1)$-$\operatorname {lca}$ vertex in *G*.

We are now in the position to prove the equivalence between (1) and (2). Clearly, Statement (2) implies (1). Suppose that *v* is a ${{\,\mathrm{\textit{k}-lca}\,}}$ vertex in *G* for some $k\ge 2$. If $k=2$, then Lemma 3.3 implies that *v* is an $\ell$-$\operatorname {lca}$ vertex in *G* for all $\ell \in \{2,\dots , |{{\,\mathrm{\texttt{C}}\,}}_G(v)|\}$. If $k\ge 3$, then repeated application of the latter statement, i.e., *v* is a $(k-1)$-$\operatorname {lca}$ shows that *v* is an $\ell$-$\operatorname {lca}$ vertex in *G* for all $\ell \in \{2,3,\dots ,k\}$. In addition, Lemma 3.3 implies that *v* is an $\ell$-$\operatorname {lca}$ vertex in *G* for all $\ell \in \{k,\dots , |{{\,\mathrm{\texttt{C}}\,}}_G(v)|\}$. In summary, *v* is an $\ell$-$\operatorname {lca}$ vertex in *G* for all $\ell \in \{2,\dots , |{{\,\mathrm{\texttt{C}}\,}}_G(v)|\}$.

It is now an easy task to verify that *G* is $\mathscr {I}^{1}$-$\operatorname {lca}$-Rel for some $\mathscr {I}^{1}$ with $|\mathscr {I}^{1}|>1$ (which implies that $\ell \in \mathscr {I}^{1}$ for some $\ell >1$) if and only if each inner vertex of *G* is a 2-$\operatorname {lca}$ vertex. Moreover, by the equivalence between (1) and (2), each inner vertex of *G* is a 2-$\operatorname {lca}$ vertex if and only if each inner vertex of *G* is a $|{{\,\mathrm{\texttt{C}}\,}}_G(v)|$-$\operatorname {lca}$ vertex and thus, satisfies $v=\operatorname {lca}_G({{\,\mathrm{\texttt{C}}\,}}_G(v))$. Consequently, each inner vertex of *G* is a 2-$\operatorname {lca}$ vertex if and only if *G* has the strong-(CL) property. In summary, Statements (3), (4) and (5) are equivalent. $\square$

Based on the latter results, we derive the following simple characterization of $\operatorname {lca}$-Rel trees.

#### Corollary 7.10

A tree is $\operatorname {lca}$-Rel if and only if it is phylogenetic. In particular, every inner vertex *v* in a phylogenetic tree *G* is an $\ell$-$\operatorname {lca}$ vertex for each $\ell \in \{2,\dots ,|{{\,\mathrm{\texttt{C}}\,}}_G(v)|\}$.

#### Proof

By Lemma 3.10, every $\operatorname {lca}$-Rel DAG is phylogenetic. Suppose that *G* is a phylogenetic tree. It is well-known that every phylogenetic tree is isomorphic to the Hasse diagram of its clustering system (Semple and Steel 2003). Hence, *G* is regular. Theorem 4.10 implies that *G* is $\operatorname {lca}$-Rel. Since $\mathfrak {C}_G$ is a hierarchy, i.e., a clustering system without overlapping clusters, we can conclude that *G* satisfies (N3O). By Lemma 7.9, every inner vertex *v* of *G* is an $\ell$-$\operatorname {lca}$ vertex for each $\ell \in \{2,\dots ,|{{\,\mathrm{\texttt{C}}\,}}_G(v)|\}$. $\square$

Note that Corollary 7.10 cannot easily be generalized to other phylogenetic DAGs with the (N3O) property. For example, consider the galled tree *G* on $X=\{x_1,x_2,x_3\}$, which has a root $\rho$ with two children $u_1$ and $u_2$, an additional edge $(u_1, u_2)$, and such that $u_1$ has only $x_1$ as child, while $u_2$ has precisely $x_2$ and $x_3$ as its children. It is easy to verify that *G* is phylogenetic, but $\rho \ne \operatorname {lca}_G(A)$ for any $A \subseteq X$, that is, *G* is not $\operatorname {lca}$-$\textsc {Rel}$. Adding a new leaf $x_4$ and the edge $(\rho , x_4)$ to *G* would result in an $\operatorname {lca}$-$\textsc {Rel}$ galled tree.

Note that the DAG *G* in Fig. 5 is a DAG with (N3O) property that is neither $\mathscr {I}^{1}$-$\operatorname {LCA}$-Rel nor $\mathscr {I}^{1}$-$\operatorname {lca}$-Rel for any $\mathscr {I}^{1}$. However, the results above allow us, together with the $\ominus$-operator, to transform a DAG *G* with (N3O) property in polynomial time into a DAG $G'$ that is $\mathscr {I}^{1}$-$\operatorname {LCA}$-Rel or $\mathscr {I}^{1}$-$\operatorname {lca}$-Rel for arbitrary $\mathscr {I}^{1}$. To this end, observe first that one can easily verify whether a given DAG with (N3O) property is an $\mathscr {I}^{1}$-$\operatorname {lca}$-Rel or $\mathscr {I}^{1}$-$\operatorname {LCA}$-Rel: Lemma 7.9 implies that we only need to verify if each inner vertex is a 2-$\operatorname {lca}$-Rel or 2-$\operatorname {LCA}$-Rel DAG; a task that can be be achieved in polynomial time (cf. Observation 7.6). Even more, we can transform any DAG *G* with (N3O) into an $\mathscr {I}^{1}$-$\operatorname {lca}$-Rel, resp., $\mathscr {I}^{1}$-$\operatorname {LCA}$-Rel DAG that satisfies (N3O) and properties (S0)–(S4), resp., (S0)–(S5) w.r.t. *G* by utilizing the following

#### Lemma 7.11

Let *G* be a DAG and *v* be an inner vertex of *G*. If *G* satisfies (N3O), then $G\ominus v$ satisfies (N3O).

#### Proof

By contraposition, assume that $G\ominus v$ does not satisfy (N3O) for some inner vertex *v* of the DAG *G*. Thus, $\mathfrak {C}_{G\ominus v}$ contains three clusters that are pairwise overlapping. By Observation 5.3 we have $\mathfrak {C}_{G\ominus v}\subseteq \mathfrak {C}_G$. Hence, $\mathfrak {C}_G$ contains three pairwise overlapping clusters and *G* does not satisfy (N3O). $\square$

Thus, whenever we found a vertex *v* that is not an $\mathscr {I}^{1}$-$\operatorname {lca}$ vertex resp., an $\mathscr {I}^{1}$-$\operatorname {LCA}$ vertex in an (N3O) DAG *G*, we can compute $G\ominus v$ in polynomial time, to derive a DAG that, by Lemma 7.11, satisfy (N3O) and, by Theorem 5.5, satisfies (S0)–(S4), resp., (S0)–(S5) w.r.t. *G*. Hence, we can reuse the latter arguments, for checking whether the remaining vertices are $\mathscr {I}^{1}$-$\operatorname {lca}$ or $\mathscr {I}^{1}$-$\operatorname {LCA}$ vertices or not and then repeat this process until no such vertices exist and always obtain a DAG satisfying (N3O) and (S0)–(S4), resp., (S0)–(S5) w.r.t *G*. We summarize the latter discussion into

#### Theorem 7.12

For a given DAG *G* that satisfies (N3O), a vertex $v\in V(G)$ and a set $\mathscr {I} \subseteq \{1,\dots ,|X|\}$, it can be verified in polynomial time if *v* is an $\mathscr {I}$-$\operatorname {lca}$ vertex or an $\mathscr {I}$-$\operatorname {LCA}$ vertex. In particular, every DAG *G* that satisfies (N3O) can be transformed in polynomial time into an $\mathscr {I}^{1}$-$\operatorname {lca}$-Rel, resp., $\mathscr {I}^{1}$-$\operatorname {LCA}$-Rel DAG with (N3O)-property and that satisfies (S0)–(S4) resp., (S0)–(S5) w.r.t. *G*.

## Summary and Outlook

In this paper, we introduced $\mathscr {I}^{1}$-$\operatorname {LCA}$-$\textsc {Rel}$ and $\mathscr {I}^{1}$-$\operatorname {lca}$-$\textsc {Rel}$ DAGs, with focus on the case when $\mathscr {I}^{1}=\{1,2,\ldots ,|L(G)|\}$, resulting in the notion of $\operatorname {LCA}$-Rel and $\operatorname {lca}$-Rel DAGs. In particular, we have shown that one can efficiently transform any given DAG *G* into an $\operatorname {LCA}$-Rel and $\operatorname {lca}$-Rel DAG *H* by stepwise removal of vertices that are not LCAs, resp., unique LCAs of any subset of taxa with the help of the $\ominus$-operator. Importantly, the resulting DAG *H* maintains significant structural features of the original DAG *G* specified by the structural properties (S1)–(S5) which also imply (S0). The simply defined and, in our opinion, rather inconspicuous $\ominus$-operator has been a somewhat surprisingly powerful tool in this paper, and may still prove to be helpful in related contexts. We characterized $\operatorname {LCA}$-$\textsc {Rel}$ and $\operatorname {lca}$-Rel DAGs and showed their close relationship to regular DAGs. Moreover, we showed that our construction indeed “simplifies” a DAG *G*, formalized through three axioms (P1)–(P3). Although we have provided polynomial-time algorithms to recognize $\operatorname {lca}$-Rel or $\operatorname {LCA}$-Rel DAGs and to transform DAGs into $\operatorname {lca}$-Rel or $\operatorname {LCA}$-Rel ones, the problem of determining if a vertex *v* is a *k*-$\operatorname {lca}$ or *k*-$\operatorname {LCA}$ vertex for a given *k* and, recognizing $\mathscr {I}^{1}$-$\operatorname {LCA}$-$\textsc {Rel}$ and $\mathscr {I}^{1}$-$\operatorname {lca}$-$\textsc {Rel}$ DAGs for specified sets $\mathscr {I}^{1}$ is an NP-complete task. The latter problems become tractable for DAGs that do not contain three pairwise overlapping clusters; a class of DAGs which includes rooted phylogenetic trees and galled-trees.

All questions posed in the introduction have been fully addressed. Question 1 is answered by the results in Sect. 3, where we demonstrate that it is possible to determine in polynomial time whether a given vertex is the (unique) LCA of a specific subset $A \subseteq L(G)$. The answer to Question 2 is two-fold. When the size of the unknown set $A \subseteq L(G)$ is unspecified, Corollary 3.6 provides a characterization, allowing Question 2 to be answered in polynomial time. However, when the size |*A*| is specified but the set *A* itself is unknown, verifying if a vertex is the (unique) LCA of a subset of leaves of size |*A*| becomes NP-complete, as shown in Sect. 7. Nevertheless, the latter type of problem becomes tractable for DAGs with N3O property. The answer to Question 3 is provided by Theorems 4.4 and 4.5, while answers to Question 4 are presented in Sects. 5 and 7.

The attentive reader may have noticed that some of the technical details introduced with the set $\mathscr {I}^{1}$ in the definitions of $\mathscr {I}^{1}$-$\operatorname {lca}$-$\textsc {Rel}$ and $\mathscr {I}^{1}$-$\operatorname {LCA}$-$\textsc {Rel}$ DAGs could have been omitted, as we primarily focused on the special case where $\mathscr {I}^{1}= \{1, 2, \ldots , |L(G)|\}$. However, since $\mathscr {I}^{1}$-$\operatorname {LCA}$-$\textsc {Rel}$ and $\mathscr {I}^{1}$-$\operatorname {lca}$-$\textsc {Rel}$ DAGs are, in particular, $\operatorname {LCA}$-Rel and $\operatorname {lca}$-Rel DAGs, respectively, most of the results also hold for the more general $\mathscr {I}^{1}$-$\operatorname {LCA}$-$\textsc {Rel}$ and $\mathscr {I}^{1}$-$\operatorname {lca}$-$\textsc {Rel}$ DAGs. Specifically, Theorem 5.5 implies that the structural properties (S0)–(S5), resp., (S0)–(S4) are preserved under the $\ominus$-operator when applied to non-$\mathscr {I}^{1}$-$\operatorname {LCA}$, resp., non-$\mathscr {I}^{1}$-$\operatorname {lca}$ vertices. Hence, these results naturally generalize those established for so-called $\operatorname {lca}$-networks (Hellmuth et al. 2023) and for the case $\mathscr {I}^{1}= \{1, 2, \dots , k-1, k\}$ (Shanavas et al. 2024). Moreover, the established results also allow for a focus on interesting subcases, such as DAGs that are $\mathscr {I}^{1}$-$\operatorname {LCA}$-$\textsc {Rel}$ for $\mathscr {I}^{1}= \{1, 2\}$ or $\mathscr {I}^{1}= \{1, 3\}$ (Barthélemy and Brucker 2008; Nowak 2009; Huber and Scholz 2018). Such DAGs play an important role in reflecting gene relationships, such as so-called orthology relations (Lafond et al. 2016; Hellmuth et al. 2013; Huber and Scholz 2018) or more general combinatorial objects (Bruckmann et al. 2022; Lindeberg et al. 2025). In particular, it is of interest to explore in greater detail which classes of DAGs and networks allow for a polynomial-time solution to check the properties $\mathscr {I}^{1}$-$\operatorname {lca}$-$\textsc {Rel}$ and $\mathscr {I}^{1}$-$\operatorname {LCA}$-$\textsc {Rel}$, as well as to transform the underlying DAGs into $\mathscr {I}^{1}$-$\operatorname {lca}$-$\textsc {Rel}$ and $\mathscr {I}^{1}$-$\operatorname {LCA}$-$\textsc {Rel}$ ones for specific sets $\mathscr {I}^{1}$. Further questions in this context include: Can we characterize DAGs and networks in which every inner vertex is a ${{\,\mathrm{\textit{k}-lca}\,}}$ vertex for a specific *k*? If such a *k* exists, what is the minimal one? Similarly, if *G* is $\operatorname {lca}$-Rel or $\operatorname {LCA}$-Rel, what is the smallest integer *k* in a subset $\mathscr {I}^{1}\subseteq \{1,2,\ldots ,|L(G)|\}$ such that *G* is $\mathscr {I}^{1}$-$\operatorname {lca}$-$\textsc {Rel}$ or $\mathscr {I}^{1}$-$\operatorname {LCA}$-$\textsc {Rel}$? By Observation 7.6, the latter task can be easily addressed when we consider whether *G* is $\{1,2\}$-$\operatorname {lca}$-Rel or $\{1,2\}$-$\operatorname {LCA}$-Rel.

We have shown that the set *W* of non-$\operatorname {LCA}$ vertices required to transform *G* into an $\operatorname {LCA}$-rel DAG $G \ominus W$, satisfying conditions (S0)–(S5), is uniquely determined. This uniqueness property is preserved for DAGs that satisfy (CL) or (PCC), i.e., for such DAGs *G*, the set *W* of non-$\operatorname {lca}$ vertices in *G* that ensures $G \ominus W$ is $\operatorname {lca}$-rel while satisfying conditions (S0)–(S4) is also unique. In general, however, the set *W* of non-$\operatorname {lca}$ vertices in *G* that makes $G \ominus W$ $\operatorname {lca}$-rel with conditions (S0)–(S4) is not unique. This raises the question of the computational complexity involved in finding a minimum-sized set *W* of non-$\operatorname {lca}$ vertices to ensure the latter.

By Proposition 5.6, if $G \ominus W$ is the $\operatorname {LCA}$-rel version of *G*, we have $\mathfrak {C}_{G \ominus W} = \mathfrak {C}_G$. In contrast, if *W* is the set of all non-$\operatorname {lca}$ vertices of *G*, Observation 5.3 implies only that $\mathfrak {C}_{G \ominus W} \subseteq \mathfrak {C}_G$. In fact, the example in Fig. 6 demonstrates that $\mathfrak {C}_{G \ominus W} \subsetneq \mathfrak {C}_G$ is possible. This raises the question of how the set systems $\mathfrak {C}_G$ and $\mathfrak {C}_{G \ominus W}$ are related, and which clusters, if any, are contained in $\mathfrak {C}_G \setminus \mathfrak {C}_{G \ominus W}$. Moreover, instead of seeking a minimum-sized set *W* of non-$\operatorname {lca}$ vertices that ensures $G \ominus W$ is $\operatorname {lca}$-rel under conditions (S0)–(S4), one might consider finding a set *W* that minimizes the size of the difference $\mathfrak {C}_G {\setminus } \mathfrak {C}_{G \ominus W}$, thereby preserving as many clusters in $\mathfrak {C}_G$ as possible in $G \ominus W$.

A further interesting generalization is as follows. For a DAG *G*, define all leaves as *pertinent*. Recursively, a non-leaf vertex is considered *pertinent* if it serves as a least common ancestor (LCA) for a subset of pertinent vertices. For example, in the DAG *G* illustrated in Fig. 5, the vertices *v* and *w* are pertinent because they are the LCA of the set $\{x,y\}$. Consequently, the non-LCA vertex $\rho$ also becomes pertinent, as it is the unique LCA of the two pertinent vertices *v* and *w*. A characterization of networks and DAGs in which all vertices are pertinent, as well as operations to transform a DAG into such a type of DAG, might be an interesting avenue for future research.

**Supplementary information**

This work is accompanied by a Python tool, SimpliDAG, which computes $\operatorname {LCA}$-Rel or $\operatorname {lca}$-Rel versions for a given DAG and is freely available on GitHub (Lindeberg and Hellmuth 2024).

## Data Availability

The data used to generate the network *N* in Fig. 9 is available at https://github.com/husonlab/phylosketch (Nov 1, 2024).
